# Defining substrate specificities of *N*-acetylglucosamine-6-*O*-sulfotransferases for enzymatic modular assembly of sulfated *O*-glycans

**DOI:** 10.1039/d6qo00226a

**Published:** 2026-04-13

**Authors:** Shuquan Fan, Jinghua Han, Tangliang Shen, MohammadHossein Shabahang, Zhenming Du, Jun Pan, Guitao Bai, Shumin Bao, Avinash Ittuveetil, Lei Li

**Affiliations:** a Department of Chemistry and Center for Diagnostics & Therapeutics, Georgia State University Atlanta GA 30303 USA lli22@gsu.edu

## Abstract

Sulfated *O*-glycans are widely distributed and play key roles in a wide range of physiological and disease processes, yet their synthesis remains challenging due to difficulties in regioselective sulfation. Here, we systematically characterized two human GlcNAc-6-*O*-sulfotransferases, CHST2 and CHST6, revealing their strong preference towards *O*-glycans (including *O*-GalNAc and *O*-mannosyl glycans) over *N*-glycans and poly-LacNAc chains. Both enzymes favored β1-6 branched GlcNAc residues, with CHST6 showing higher activity and broader substrate tolerance than CHST2. Guided by these insights, we established a modular enzymatic assembly platform for efficient synthesis of 32 well-defined sulfated *O*-GalNAc, *O*-mannosyl glycans, and *O*-glycopeptides. This streamlined strategy enables versatile access to sulfated *O*-glycans and provides a general route for constructing other classes of sulfated glycans.

## Abstract 2

The substrate specificity of CHST2 and CHST6 demonstrated a strong preference for sulfating β1-6 linked terminal GlcNAc residues on *O*-glycans, enabling the enzymatic modular construction of diverse sulfated *O*-glycan structures.

## Graphical abstract

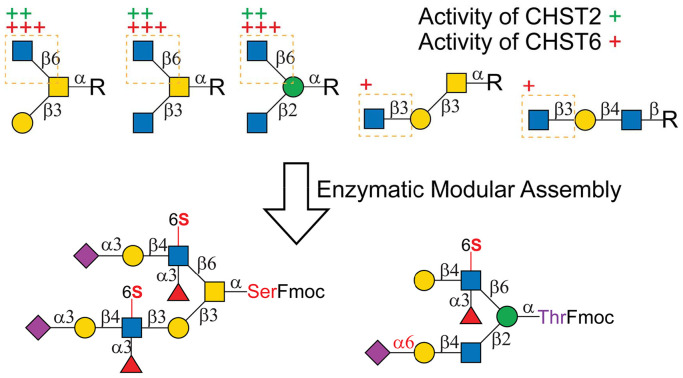

## Introduction

Sulfation is the most abundant glycan modification, which significantly impacts the structure and functions of residing glycans.^1^ The sulfated glycans of the linear glycosaminoglycan (GAG) class, including heparan sulfate (HS), chondroitin sulfate (CS), and keratan sulfate (KS), are found to be ubiquitously expressed on the cell surfaces of all mammals, where they regulate extracellular cell signaling, growth and homeostasis, and provide structural support.^2,3^ Importantly, sulfation is not restricted to linear GAGs, but is also frequently identified on complex *O*-glycans, which play critical roles in a variety of biological processes. For example, peripheral node addressin (PNAd), a set of sialomucins including GlyCAM-1 and CD34, carries *O*-GalNAc glycans that present the 6-sulfated sialyl-Lewis X (6-sulfo-sLe^*x*^) epitope on core 2 and extended core 1 branches.^4–6^ Such *O*-glycans function as ligands for l-selectin, mediating lymphocyte homing and inflammatory responses.^7,8^ These ligands were often identified using a MECA-79 mAb that specifically recognizes a sulfated extended core-1 structure.^4^ In addition, highly sulfated *O*-glycans correlate with various diseases, *e.g.*, abnormally sulfated mucins appear to be central to respiratory infections in patients with cystic fibrosis,^9^ and numerous sulfated *O*-GalNAc glycans have been identified on a variety of tumor cells and tissues,^10–12^ some as tumor biomarker candidates.^13^ A recent study identified 83 *O*-glycan compositions on MUC2, MUC5AC, and MUC5B, among which 51 were mono-sulfated and 20 were di-sulfated,^14^ further underscoring the widespread prevalence of sulfated *O*-glycans.^15–18^ These *O*-glycans, together with non-sulfated ones, coat the colon surface to form a protective barrier against gut microbes.^19^*O*-mannosyl glycans, accounting for over 30% of all *O*-glycans in brain tissues,^20^ have also been found to carry sulfate groups. These structures typically contain the HNK-1 epitope^21^ and play important roles in brain development and remyelination.^22,23^ Notably, recent studies have identified core m1 and core m2 *O*-mannosyl glycans with distinct sulfation patterns in brain tissues^24^ and mucins.^19^

**Fig. 1:**
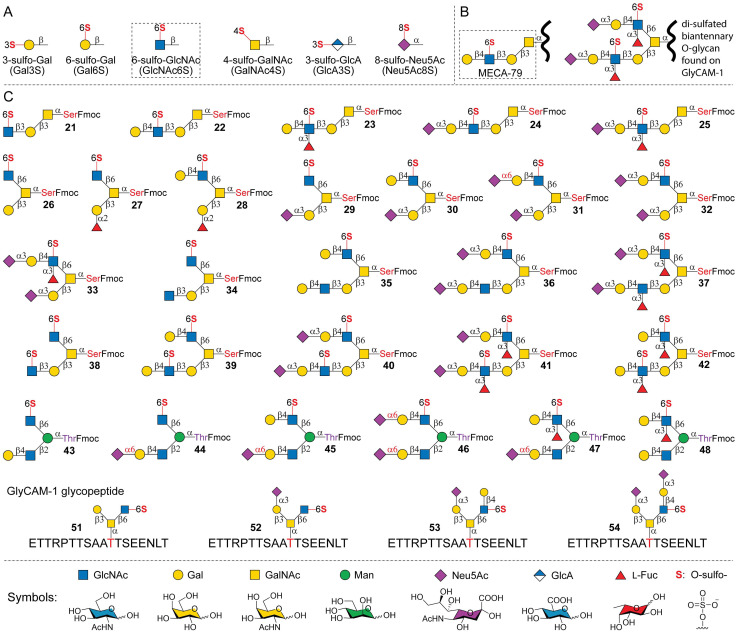
Common non-GAG sulfations and sulfated *O*-glycans synthesized in this work. (A) Typical sulfation motifs found on mammalian *O*-glycans; (B) examples of identified sulfated *O*-glycan epitopes; and (C) sulfated *O*-glycans and glycopeptides prepared in this study. GlcNAc, *N*-acetylglucosamine; Gal, galactose; GalNAc, *N*-acetylgalactosamine; Man, mannose; Neu5Ac, *N*-acetylneuraminic acid; GlcA, glucuronic acid; l-Fuc, l-fucose; Ser, serine; Thr, threonine; and Fmoc, fluorenylmethyloxycarbonyl.

All sulfations on *N*-/*O*-glycans are oxygen-linked (*O*-sulfation), with only a few sulfated motifs being documented (Fig. 1A). Among these, 6-sulfo-GlcNAc, 6-sulfo-Gal, and 3-sulfo-Gal are common to both *O*-glycans and *N*-glycans,^25^ whereas 4-sulfo-GalNAc has been reported almost exclusively on *N*-glycans, capping the 4′-sulfated-LDN epitope.^25,26^ The motif 3-sulfo-GlcA is a characteristic of the HNK-1 epitope often terminating *O*-mannosyl glycans, *N*-glycans, and glycolipids on neural tissues.^27^ Sialic acids can also be *O*-sulfated at the C8 position (8-sulfo-Neu5Ac or 8-sulfo-Neu5Gc), which were found in various vertebrate cells and tissues, even though underlying glycan scaffolds have yet to be defined.^28^ Despite limited sulfation motifs, sulfated *O*-glycans are highly diversified. Such heterogeneity arises from the extensive branching and varied *O*-glycan core structures, offering numerous sulfation sites. Nevertheless, recent advances have highlighted diverse functional roles of sulfated non-GAGs, particularly their interactions with Siglecs and other glycan-binding proteins (GBPs),^29–31^ although detailed structure–function relationships for individual structures remain largely underexplored.

The widespread occurrence and diverse functions of sulfated glycans have spurred significant recent interest in synthesizing these molecules. For instance, a variety of sulfated glycan determinants have been chemoenzymatically synthesized in which sulfates are chemically installed onto primary alcohols (C6-OH) of Gal, GlcNAc, GlcNAc, or Glc residues due to their relatively high reactivity.^32,33^ Chemoenzymatic synthesis of gangliosides with 3-sulfo-Gal, 6-sulfo-GalNAc, or the HNK-1 epitope, core 2 *O*-GalNAc glycan with 6-sulfo-GlcNAc, and HNK-1-presenting *O*-mannosyl glycans was achieved, where sulfates were also chemically installed.^34–38^ Biosynthetically, sulfates are transferred from 3′-phosphoadenosine-5′-phosphosulfate (PAPS) to specific positions of glycans by sulfotransferases (sulfoTs). Recent advances in the expression and application of sulfoTs have enabled the synthesis of complex sulfated glycans. For example, Boons and coworkers prepared an array of KS structures utilizing CHST1 and CHST2.^39,40^ Wang and coworkers applied CHST1, CHST2, and Gal3ST2 for the enzymatic synthesis of sulfated biantennary *N*-glycans.^41,42^ During the preparation of this work, Wen *et al.* reported the *de novo* synthesis of sulfated multi-antennary *N*-glycans using similar sulfoTs as well as CHST6.^43^ In a different approach, Withers and co-workers recently reported an engineered 6-sulfo-GlcNAcase capable of directly transferring 6-sulfo-GlcNAc to generate a few sulfated *N*-glycans and extended core 1 *O*-glycans, including the MECA-79 epitope.^44^ Further application of these engineered glycosidases in the synthesis of sulfated complex glycans remains to be evaluated due to the lack of specificity towards various acceptors. Nevertheless, chemoenzymatic synthesis of well-defined sulfated *O*-glycans is scarce,^38^ especially complex structures such as the di-sulfated biantennary *O*-GalNAc glycan found in GlyCAM-1 (Fig. 1B). We have focused our efforts on developing synthetic strategies to access *O*-glycans and glycopeptides.^45–48^ In this work, we target sulfated *O*-glycans, specifically those bearing the abundant 6-sulfo-GlcNAc motif.

*In vivo*, *N*-acetylglucosamine-6-*O*-sulfotransferase (GlcNAc6ST) catalyzes the 6-*O*-sulfation of non-reducing end GlcNAc residues to generate 6-sulfo-GlcNAc in Golgi.^49–52^ Humans have five GlcNAc6ST isoenzymes, CHST2 (GlcNAc6ST-1), CHST4 (GlcNAc6ST-2), CHST5 (GlcNAc6ST-3), CHST6 (GlcNAc6ST-5), and CHST7 (GlcNAc6ST-4). CHST2 was first identified in human bronchial mucosa, which sulfates the terminal branching GlcNAc on the core 2 *O*-GalNAc glycan.^51^ Substrate specificity studies using radiolabeled PAPS demonstrated similar activity to *O*-mannosyl glycans (GlcNAcβ1-2Man and GlcNAcβ1-6Man), but not to *O*-GalNAc core 3 (GlcNAcβ1-3GalNAc).^50^ Its activity toward a linear keratan pentasaccharide was substantially lower,^50^ although recent studies successfully employed it to synthesize KS.^39,40^ In the context of biantennary *N*-glycans, Wang and coworkers demonstrated that CHST2 preferentially sulfates the GlcNAc residue on the α1-3Man branch, although the α1-6Man branch can also be sulfated with excess PAPS and prolonged incubation.^41,42^ CHST4, also known as HEC-GalNAc6ST, has a similar substrate specificity (except that it also sulfates *O*-GalNAc core 3) but lower activity.^50^ CHST5 and CHST6, also known as I-GlNAc6ST and C-GlcNAc6ST, respectively, showed the highest activity toward *O*-GalNAc core 2 but also recognized multi-branched *N*-glycans and *O*-GalNAc extended core 1.^49^ Lastly, CHST7 not only preferentially acts on mannose-linked GlcNAc but can also act on *O*-GalNAc core 2 and the GalNAc residues of chondroitin, and therefore it is also referred to as C6ST-2.^53,54^ Nevertheless, nearly all prior characterization studies relied on radiolabeled PAPS, which occasionally yielded inconsistent results. A clear understanding of their substrate specificity toward diverse glycan structures is highly demanded to guide synthetic efforts. In this work, we overexpressed human CHST2 and CHST6, explored their activities toward 20 acceptor substrates, and discovered their strong preference toward *O*-glycans, particularly GlcNAcβ1-6 branches. Using both sulfoTs and eight robust glycosyltransferases (GTs), we achieved enzymatic modular assembly of 32 sulfated *O*-glycans and *O*-glycopeptides (Fig. 1C).

## Results and discussion

### CHST2 and CHST6 strongly prefer the GlcNAcβ1-6 branch of *O*-glycans

**Fig. 2:**
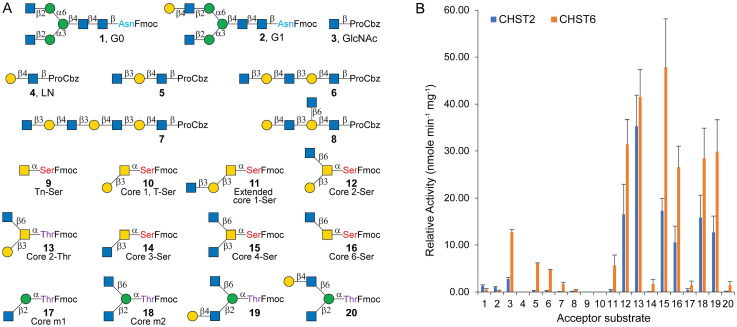
Substrate specificity studies of CHST2 and CHST6. A. Structures of the tested acceptor substrates; B. relative activity of the sulfoTs toward the 20 acceptors. LN, LacNAc; Ser, serine; Thr, threonine; and Fmoc, 9-fluorenylmethoxycarbonyl.

Among the five GlcNAc6STs, CHST2 and CHST6 were selected for expression and activity assay due to their relatively high expression levels.^55^ They were expressed in HEK293 cells (see Methods for details), yielding 65 mg L^−1^ and 15 mg L^−1^ CHST2 and CHST6, respectively, following one-step Ni-NTA affinity purification (Fig. S1). Both enzymes were evaluated for their substrate specificity against a panel of 20 glycan structures (Fig. 2A), including *N*-glycans (1, 2), linear poly-LacNAc oligosaccharides (3–8),^56^ mucin-type *O*-glycan cores (9–16),^46^ and *O*-mannosyl glycans (17–20).^47^ As shown in Fig. 2B and Table S1, neither CHST2 nor CHST6 sulfated LacNAc (4), Tn (9), or T-antigen (10), consistent with their strict recognition of non-reducing terminal GlcNAc residues.^49–52^ CHST2 sulfates both G0 (1) and G1 (2) with low but comparable activities (1.34 and 1.02 nmol min^−1^ mg^−1^). A di-sulfated product was not observed for G0, consistent with previous reports, which suggested a preference to the GlcNAcβ1-2Manα1-3 branch.^41,42^ The activity of CHST2 toward the GlcNAc monosaccharide (3) is approximately two-fold (2.75 nmol min^−1^ mg^−1^) that toward *N*-glycans. However, its activity toward terminal GlcNAc on linear poly-LacNAc chains (5–7) is over 10-fold lower (0.08–0.28 nmol min^−1^ mg^−1^), suggesting that CHST2 is unlikely to be responsible for the synthesis of keratan sulfates.^50^ Surprisingly, CHST2 showed much higher activity toward *O*-GalNAc and *O*-mannosyl glycans bearing GlcNAcβ1-6 branches (12, 15, 16, 18, and 19), reaching 10.54–17.26 nmol min^−1^ mg^−1^, about 8–12 fold that toward *N*-glycan G0 (Fig. 2B). In contrast, CHST2 exhibited negligible activity toward *O*-GalNAc extended core 1 (11), core 3 (14), *O*-mannosyl core m1 (17), and compound 20, all of which lack a terminal unmodified β1,6-linked GlcNAc residue. Notably, its sulfation preference does not extend to all β1-6linked GlcNAc, as the activity toward compound 8, poly-LacNAc presenting an I-branched GlcNAc, was negligible. Another surprising observation was that the attached amino acid affected activity, with Thr-linked core 2 (13) exhibiting twice the activity (35.27 nmol min^−1^ mg^−1^) of Ser-linked core 2 (12, 16.52 nmol min^−1^ mg^−1^). Collectively, these results indicate that CHST2 strongly prefers β1-6linked branching GlcNAc on *O*-glycans.

CHST6 displayed broader substrate specificity and higher activity than CHST2 toward most tested substrates, with only exceptions being *N*-glycans G0 and G1 (Fig. 2B and Table S1). CHST6 showed substantially higher activity than CHST2 toward linear poly-LacNAc chains (5–7). For example, its activity toward trisaccharide 5 was 22-fold higher (6.09 nmol min^−1^ mg^−1^) than that of CHST2. Interestingly, the activity of CHST6 declined progressively with increased lengths of poly-LacNAc chains (relative activities of 12.74, 6.09, 4.73, and 1.72 nmol min^−1^ mg^−1^ towards monosaccharide (3), trisaccharide (5), pentasaccharide (6), and heptasaccharide (7)). This result suggests a sensitivity to the glycan chain length in CHST6 substrate recognition. Similar to CHST2, CHST6 strongly prefers *O*-GalNAc and *O*-mannosyl glycans with β1-6linked branching GlcNAc (12, 15, 16, 18, and 19), and its activities are approximately two-fold those of CHST2, with the highest relative activity observed toward *O*-GalNAc core 4 (15), reaching 47.86 nmol min^−1^ mg^−1^. Notably, CHST6 also showed medium activity (5.63 nmol min^−1^ mg^−1^) toward extended core 1 11, about 15-fold that of CHST2 (0.36 nmol min^−1^ mg^−1^). Meanwhile, CHST6 could sulfate *O*-GalNAc core 3 (14), *O*-mannosyl core m1 (17) and compound 18, although with lower activities (1.39–1.7 nmol min^−1^ mg^−1^), which are comparable to those of *N*-glycans, suggesting its broader substrate specificity.

Taken together, while both CHST2 and CHST6 have been applied for the synthesis of sulfated *N*-glycans,^41–43^ they strongly prefer the β1-6linked GlcNAc branches on *O*-glycans. In comparison, CHST2 shows relatively high activity toward *N*-glycans, whereas CHST6 displays higher activity toward all other tested substrates, particularly less preferred structures such as *O*-GalNAc extended core 1, core 3, *O*-mannosyl core m1, and poly-LacNAc chains, making it a superior synthetic catalyst toward various sulfated *O*-glycans and keratan sulfates.

### Enzymatic modular assembly of *O*-GalNAc glycans with mono- and di-sulfation

**Fig. 3:**
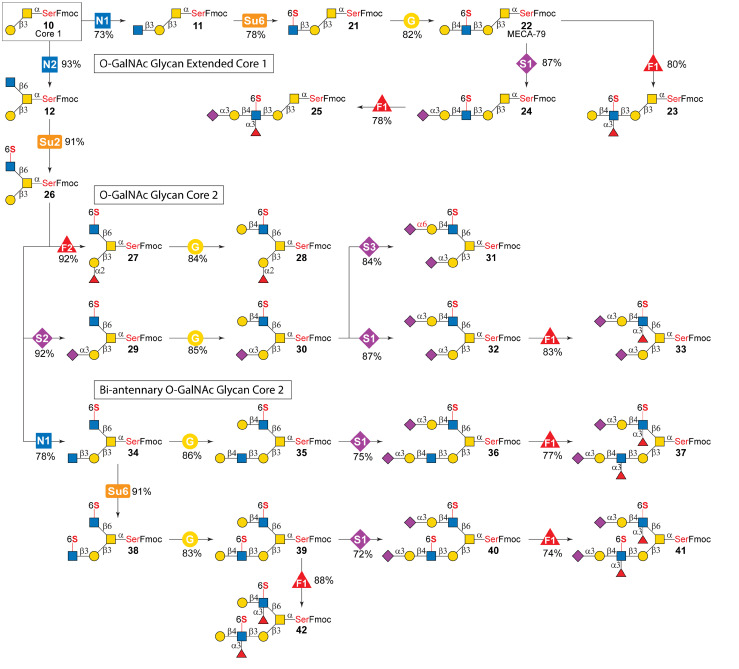
Enzymatic modular assembly of sulfated *O*-GalNAc glycans. Module N1, β1-3GlcNAcylation catalyzed by *H. pylori* β1-3-*N*-acetylglucosaminyltransferase (HpLgtA)^57^ in the presence of uridine-diphosphate-GlcNAc (UDP-GlcNAc); module N2, β1-6GlcNAcylation catalyzed by human GCNT1 in the presence of UDP-GlcNAc; module G, β1-4galactosylation catalyzed by *H. pylori* β1-4galactosyltransferase (HpLgtB)^59^ in the presence of UDP-Gal; module Su2, GlcNAc-6-*O*-sulfation catalyzed by CHST2 in the presence of PAPS; module Su6, GlcNAc-6-*O*-sulfation catalyzed by CHST6 in the presence of PAPS; module F1, α1-3 fucosylation catalyzed by *H. pylori* α1-3/4 fucosyltransferase C-terminal 66 amino acid truncation (Hp3FT)^60^ in the presence of guanosine 5′-diphospho-l-fucose (GDP-Fuc); module F2, α1-2fucosylation catalyzed by *H. mustelae* α1-2fucosyltransferase (Hm2FT)^61^ in the presence of GDP-Fuc; module S1, α2-3sialylation catalyzed by human ST3Gal4 in the presence of cytidine 5′-monophospho-*N*-acetylneuraminic acid (CMP-Neu5Ac); module S2, α2-3sialylation catalyzed by human ST3Gal1 in the presence of CMP-Neu5Ac; module S3, α2-6sialylation catalyzed by *P. damselae* α2-6sialyltransferase (Pd26ST)^60^ in the presence of CMP-Neu5Ac.

Knowing the detailed substrate specificities of CHST2 and CHST6, we devised an enzymatic modular assembly strategy to prepare diverse sulfated *O*-GalNAc glycans (Fig. 3). The enzymatic assembly began with chemically prepared *O*-GalNAc core 1-Ser (Fmoc-protected) (10).^46^ The hydrophobic, UV-detectable Fmoc group facilitated reaction monitoring and enabled reverse-phase (RP) chromatography purification.^56^ To synthesize extended core 1 structures with 6-*O*-sulfation (21–25), we first employed a β1-3GlcNAcylation module (module N1) to produce 11. The module includes *Helicobacter pylori* β1-3-*N*-acetylglucosaminyltransferase (HpLgtA)^57^ and the sugar donor UDP-GlcNAc. Although a previous report achieved 90% yield when core 1 was attached to threonine (Thr),^57^ our initial reactions gave only moderate conversion (44%), suggesting that the activity of HpLgtA may be influenced by attached amino acids or the bulky Fmoc group. A homolog from *Neisseria meningitidis* (NmLgtA)^58^ was also tested, which gave minimum conversions. Nevertheless, by supplying 5 fold sugar donor, and extending the reaction time, a good yield of 73% was achieved using HpLgtA, producing 27 mg of 11 after a one-step RP chromatography purification. With compound 11 in hand, CHST6 was selected to sulfate GlcNAc in the presence of 1.5 equivalents of PAPS (module Su6), given its superior activity, affording 21 in a good yield of 78%. NMR analysis of 21 confirmed the 6-*O*-sulfation on GlcNAc (SI). Subsequent sequential β1-4galactosylation (module G enabled by *H. pylori* β1-4galactosyltransferase HpLgtB^59^), α2-3sialylation (module S1 enabled by human ST3Gal4), and α1-3fucosylation (module F1 enabled by *H. pylori* α1-3fucosyltransferase Hp3FT ^60^) of 21 produced 22 (MECA-79 epitope), 24, and 25, respectively, in satisfactory yields of 78–87%. Finally, module F1 catalyzed fucosylation of 22 afforded 23 in 80% yield. These results indicate that 6-sulfo-GlcNAc is well tolerated by these GTs.

We next aimed to synthesize *O*-GalNAc core 2 and biantennary core 2 glycans with defined sulfation patterns (Fig. 3). Previously, we reported the chemical synthesis of core 2 at a 300 mg scale, which required additional chemical glycosylation steps from core 1.^46^ Alternatively, the enzymatic synthesis of core 2 was attempted in this work, using the branching enzyme GCNT1 expressed in HEK293 cells (18 mg L^−1^ culture) (Fig. S1). This approach (module N2) afforded 145 mg of core 2 (compound 12) in an excellent yield of 93%. Subsequent 6-*O*-sulfation of β1-6GlcNAc to produce 26 was achieved by module Su2 (catalyzed by CHST2) in an excellent yield of 91%. CHST2 was selected, given its much higher expression level (four times that of CHST6). With 26 in hand, mono-sulfated structures (27–33) were prepared through enzymatic modular assembly with well programmed module sequences (Fig. 3). For example, α1-2fucosylation (module F2 catalyzed by *H. mustelae* α1-2fucosyltransferase Hm2FT ^61^) and subsequent β1-4galactosylation (module G) produced 27 and 28 in 92% and 84% yields, respectively. To synthesize 29, human ST3Gal1 (Fig. S1) was employed (module S2) instead of a previously widely used *Pasteurella multocida* α2-3sialyltransferase 1 mutant, M144D, due to its residual hydrolysis activity.^62^ Later β1-4galactosylation (module G), α2-6sialylation (module S3 catalyzed by *Photobacterium damselae* α2-6sialyltransferase Pd26ST),^60^ α2-3sialylation (module S1), and α1-3fucosylation (module F1) of the β1-6GlcNAc branch on 29 afforded 30–33 in very good yields (83–87%).

The synthesis of biantennary core 2 structures was initiated by β1-3GlcNAcylation (module N1) of 26 in the presence of excess amounts of donor, HpLgtA, and elongated incubation, affording 34 in a yield of 78%. Mono-sulfated biantennary *O*-glycans 35, 36, and 37 were then prepared by sequential assembly using modules G, S1, and F1 (Fig. 3) in good to very good yields (75–86%). Finally, to synthesize the di-sulfated biantennary *O*-glycan found on GlyCAM-1 (41), CHST6 was used to sulfate the β1-3linked GlcNAc (module Su6), given its much higher activity, affording the di-sulfated product (38) in an excellent yield of 91%. Subsequent sequential β1-4galactosylation (module G), α2-3sialylation (module S1), and α1-3fucosylation (module F1) then afforded symmetric di-sulfated biantennary *O*-glycans 39, 40, and 41, respectively, in good yields. Additionally, 42 was obtained by α1-3fucosylation (module F1) of both branches of 39 in a nearly excellent yield (88%). Together, the results demonstrate the efficiency of this sequence-controlled enzymatic modular assembly for synthesizing complex sulfated glycans.

### Modular assembly of sulfated *O*-mannosyl glycans

**Fig. 4:**
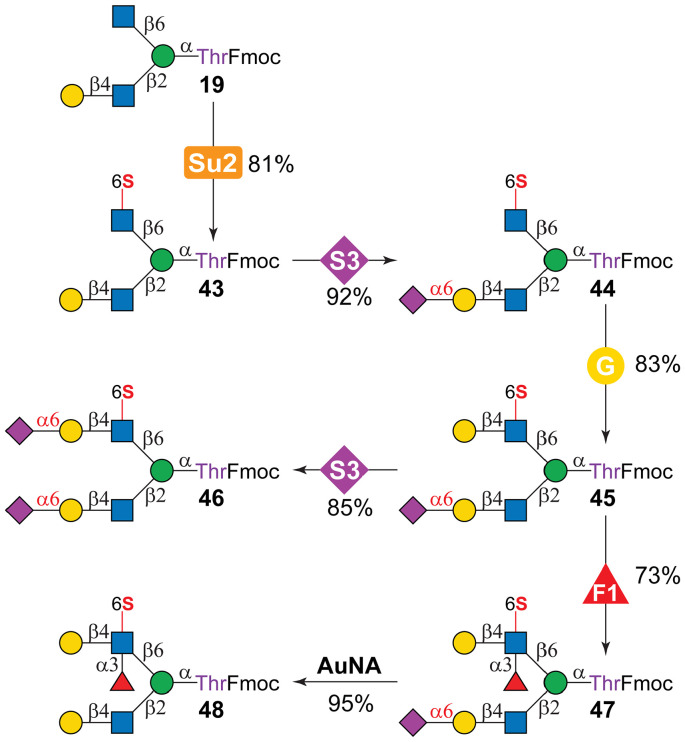
Enzymatic modular assembly of asymmetric sulfated *O*-mannosyl glycans. Module G, β1-4galactosylation catalyzed by HpLgtB in the presence of UDP-Gal; module Su2, GlcNAc-6-*O*-sulfation catalyzed by CHST2 in the presence of PAPS; module F1, α1-3fucosylation catalyzed by Hp3FT in the presence of GDP-Fuc; module S3, α2-6sialylation catalyzed by Pd26ST in the presence of CMP-Neu5Ac; AuNA, *A. ureafaciens* neuraminidase.^56^

Many sulfated *O*-mannosyl glycans have been identified in brain tissues and mucins;^19,24^ however, only those containing the HNK-1 epitope have been synthesized to date.^34^ To showcase the broad applicability of the enzymatic assembly strategy, sulfated asymmetric *O*-mannosyl core m2 glycans were prepared. As illustrated in Fig. 4, a chemically prepared asymmetric core m2 glycan, 19,^47^ which carries a β1-4Gal unit on the β1-2GlcNAc branch, was used as the starting substrate to achieve selective sulfation and glycosylation. Treatment with module S2 selectively sulfated the β1-6GlcNAc of 19 given the restricted recognition of terminal GlcNAc residues by CHST2, affording 43 in 81% yield. Subsequent α2-6sialylation of the β1-2GlcNAc branch (module S3) and β1-4galactosylation of the β1-6GlcNAc branch (module G) then gave 44 and 45 in 92% and 83% yields, respectively. Further treatment of 45 with module S3 yielded 46. In addition, the reaction of 45 with module F1 enabled branch-selective α1-3fucosylation of the β1-6branch in 73% yield, as the α2-6Neu5Ac on the β1-2branch prevented Hp3FT-catalyzed fucosylation as expected.^63^ Finally, desialylation of 47 using *A. ureafaciens* neuraminidase (AuNA) furnished the asymmetrically fucosylated and sulfated *O*-mannosyl glycan 48 in an excellent yield of 95%.

### Enzymatic modular synthesis of a sulfated *O*-glycopeptide

**Fig. 5:**
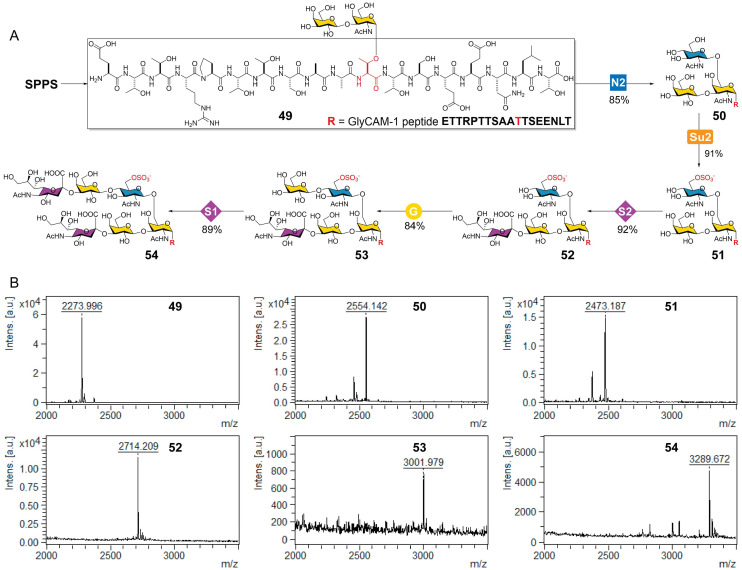
Enzymatic modular synthesis of sulfated *O*-glycopeptides (A) and their MALDI-MS spectra (B). SPPS, solid-phase peptide synthesis; module N2, β1-6GlcNAcylation catalyzed by GCNT1 in the presence of UDP-GlcNAc; module G, β1-4galactosylation catalyzed by HpLgtB in the presence of UDP-Gal; module Su2, GlcNAc-6-O-sulfation catalyzed by CHST2 in the presence of PAPS; module S1, α2-3sialylation catalyzed by human ST3Gal4 in the presence of CMP-Neu5Ac; module S2, α2-3sialylation catalyzed by human ST3Gal1 in the presence of CMP-Neu5Ac.

We next sought to prepare the GlyCAM-1 glycopeptide presenting the di-sulfated biantennary *O*-glycan following the same synthetic route illustrated in Fig. 3 (from compounds 10 to 41). As shown in Fig. 5, the synthesis began with solid-phase peptide synthesis (SSPS) by incorporating a peracetylated core 1-Thr building block^48^ as the eleventh amino acid (Thr91), affording the GlyCAM-1 peptide (E_81_TTRPTTSAAT_91_TSEENLT) bearing *O*-glycan core 1 (49). Subsequent treatment of 49 with module N2 (catalyzed by GCNT1) and module Su2 (catalyzed by CHST2) yielded the core 2 glycopeptide (50) and the 6-*O*-sulfated core 2 glycopeptide (51) in high yields of 85% and 91%, respectively. However, the following β1-3GlcNAc extension catalyzed by HpLgtA (module N1) failed, giving less than 5% conversion even with excess donor, enzyme, and prolonged incubation, further indicating that HpLgtA activity is strongly affected by the surrounding structural context. Alternatively, 51 was elaborated to generate sulfated glycopeptides 52, 53, and 54 through sequential glycosylation with module S2 (catalyzed by ST3Gal1), module G (catalyzed by HpLgtB), and module S1 (catalyzed by ST3Gal4), affording desired products in very high yields of 84–92%. The inability of HpLgtA to act on glycopeptides also agrees with our observations that many bacterial-origin GTs exhibit poor activity on glycoconjugate substrates (unpublished). As an alternative, mammalian core 1-extending enzyme β-1-3-*N*-acetylglucosaminyltransferase 3 (B3GNT3)^4^ can be evaluated. In summary, while most established enzyme modules are readily applicable for the synthesis of glycopeptides or glycoproteins, certain modules empowered by bacterial GTs exhibit limited compatibility. The development of new synthetic modules employing mammalian glyco-related enzymes will further expand the synthetic toolbox for complex glycoconjugate assembly.

## Conclusion

We systematically studied the substrate specificity of two human GlcNAc-6-*O*-sulfotransferases and revealed their pronounced preference toward *O*-glycans, including mucin-type *O*-GalNAc and *O*-mannosyl glycans, over *N*-glycans and linear poly-LacNAc chains (keratan). A secondary but more important feature of their selectivity lies in their strong preference for the β1-6 branching GlcNAc in *O*-glycans, but not I-branched GlcNAc. In comparison, CHST6 displayed higher overall activity toward nearly all tested structures than CHST2, except for the branching GlcNAc residues on *N*-glycans. Furthermore, CHST6 exhibited broader substrate tolerance, acting on various *O*-glycan cores and linear poly-LacNAc chains, whereas CHST2 showed very low or negligible activity toward GlcNAc residues on poly-LacNAc chains and β1-2/3 branching GlcNAc on *O*-glycans. Notably, the activity of CHST6 diminishes in a length-dependent manner toward the poly-LacNAc chain. These findings on substrate specificity and enzymatic activity provide valuable guidelines for the strategic employment of GlcNAc 6-*O*-sulfotransferases in the efficient synthesis of sulfated glycans.

Building on this knowledge, we developed an efficient modular assembly platform for the rapid synthesis of sulfated *O*-glycans and glycopeptides by integrating GT modules. Most enzyme modules demonstrated high efficiency toward both sulfated *O*-glycans and glycopeptides, whereas the bacterial HpLgtA module was incompatible with glycopeptide substrates. Another important observation is that all GTs employed in this study tolerated GlcNAc 6-*O*-sulfation, underscoring the compatibility of sulfation with subsequent enzymatic extensions. Overall, this modular assembly strategy provides a streamlined and versatile platform for synthesizing diverse sulfated *O*-glycans. Coupled with emerging methods for large-scale PAPS synthesis^64^ and regeneration,^65^ our findings open the door to straightforward and scalable access to diverse sulfated glycans and glycoconjugates.

## Author contributions

S. F. and L. L. conceived the project; S. F. performed enzyme assay and modular synthesis; J. H., T. S., and Z. D. helped with data interpretation; J. H., T. S., and A. I. performed chemical synthesis; J. P. G. B., and S. B. helped with enzymatic modular synthesis. L. L. and S. F. wrote the manuscript which was edited and approved by all authors.

## Conflicts of interest

There are no conflicts to declare.

## Supplementary Material

QO-013-D6QO00226A-s001

## Data Availability

The data supporting this article have been included as part of the supplementary information (SI). Supplementary information is available. See DOI: https://doi.org/10.1039/d6qo00226a.
