# Supplementary material for: Defining substrate specificities of N-acetylglucosamine-6-O-sulfotransferases for enzymatic modular assembly of sulfated O-glycans
Source: Org Chem Front. 2026 Apr 13;13(11):3414–24. doi: 10.1039/d6qo00226a (PMC13098632; doi:10.1039/d6qo00226a)
Supplement: QO-013-D6QO00226A-s001 [file QO-013-D6QO00226A-s001.pdf]

This journal is © the Partner Organisations 2026

SUPPORTING INFORMATION

Defining Substrate Specificities of *N*-Acetylglucosamine-6-O-Sulfotransferases for Enzymatic Modular Assembly of Sulfated O-Glycans

Shuquan Fan, Jinghua Han, Tangliang Shen, MohammadHossein Shabahang, Zhenming Du, Jun Pan, Guitao Bai, Shumin Bao, Avinash Ittuveetil, and Lei Li*

Department of Chemistry and Center for Diagnostics and Therapeutics, Georgia State University, Atlanta, GA 30303, USA

*To whom correspondence should be addressed: lli22@gsu.edu

Table of Contents

I. Materials……………………………………………………………………Page S2

II. Methods……………………………………………………………………..Page S2

III. Supplementary Figure and Tables …………………………………..………...Page S5

IV. NMR and Mass Data of Synthesized Compounds.……….………………….......Page S6

V. HPLC Analysis of Purified Compounds.…………..…… ………………..…......Page S21

VI. NMR Spectra ……………..…………………………………………………....Page S33

VII. References…………………………………………………………………...Page S76

I. Materials

All chemicals were purchased from Fisher Scientific (Hampton, NH), unless otherwise stated. Monosaccharides and nucleotides used for the synthesis of sugar nucleotides were obtained from Biosynth (Gardner, MA). Sulfate donor PAPS was purchased from Glycan Therapeutics (Raleigh, NC). All HPLC columns were purchased from Waters Corp. (Milford, MA) unless otherwise stated. Bio-Gel P2 and P4 resins were obtained from Bio-Rad Laboratories (Hercules, CA). PAPS was obtained from Glycan therapeutics (Raleigh, NC).

Expression vectors for N-terminal GFP-tagged human enzymes CHST2, CHST6, GCNT1, ST3Gal1, and ST3Gal4 were obtained from Glyco Expression Technologies, Inc (Athens, GA). *H. pylori* β1-3-*N*-acetylglucosaminyltransferase (HpLgtA),[1] *H. pylori* β1-4galactosyltransferase (HpLgtB),[2] *H. pylori* α1-3/4 fucosyltransferase C-terminal 66 amino acid truncation (Hp3FT),[3] *H. mustelae* α1-2fucosyltransferase (Hm2FT),[4] and *Photobacterium damselae* α2-6sialyltransferase (Pd26ST)[3] were prepared as previously reported. *Arthrobacter ureafaciens* neuraminidase (AuNA) was purchased from Nacalai Teaque, Inc (Japan). Sugar nucleotide donors including cytidine uridine-5'-diphosphate-galactose (UDP-Gal), uridine-5'-diphosphate-N-acetylglucosamine (UDP-GlcNAc), guanosine 5'-diphospho-L-fucose (GDP-Fuc), and 5'-monophospho-*N*-acetylneuraminic acid (CMP-Neu5Ac) were prepared as reported previously[5, 6] with brief P2-chromatography to >70% purity, freeze-dried and stored at -20 °C for long-term use.

II. Methods

<u>Expression and Purification of Human Enzymes</u>

Human CHST2, CHST6, GCNT1, ST3Gal1, and ST3Gal4 with a N-terminal His-tag and a GFP tag were expressed and purified as reported.[7, 8] Briefly, expression plasmids were extracted and transfected into HEK 293-F cell line for expression for 4 days. The culture medium was then collected with brief centrifugation to remove cells, mixed with equal volumes of binding buffer (50 mM Tris-HCl, 300 mM NaCl, and 20 mM imidazole at pH 7.5) and applied directly onto a gravity column with 3 mL of HisPur Ni-NTA Resin (Thermo Fisher) that is preequilibrated with the binding buffer. The flow through was collected and loaded one more time. The resin was washed with 50 mL of binding buffer and then 50 mL of washing buffer (50 mM Tris-HCl, 300 mM NaCl, and 50 mM imidazole at pH 7.5). Finally, target proteins were eluted with 10 mL of elution buffer (50 mM Tris-HCl, 300 mM NaCl, and 250 mM imidazole at pH 7.5), and desalted against storage buffer (50 mM HEPES, pH 7.0, 150 mM NaCl, 20% Glycerol) for long-term storage at -80 °C. Eluted proteins were analyzed by SDS-PAGE (Figure S1) and their concentrations were determined using a Nanodrop spectrophotometer.

<u>Substrate specificity study of CHST2 and CHST6</u>

Compounds **1−20** used for substrate specificity studies were prepared as previously reported.[9-11] Reactions were carried out in 20 μL systems containing 100 mM MES buffer (pH 6.5), $MgCl_2$ (10 mM), 1 mM of ATP, suitable amounts of sulfoTs (0.065 mg/mL of CHST2 or 0.04 mg/mL), and appropriate concentrations of acceptors and PAPS. For acceptors **1**, **2**, and **9**–**20**, 2.4 mM of each compound and 10 mM of PAPS were used; for acceptors 3–8, 100 mM of each compound and 200 mM of PAPS were used. The reactions were incubated at 37 °C for 0.5–4 hours. Aliquots (1 mL) were withdrawn at defined time points when conversion range between 20-40%, quenched with 0.1% trifluoroacetic acid (TFA), and analyzed by HPLC. Analyses were performed on an XBridge Peptide BEH C18 column (130 Å, 5 mm,

4.6 × 250 mm) using solvent A (water with 0.1% TFA) and solvent B (acetonitrile with 0.1% TFA). A linear gradient of 5–35% B over 20 min at 1 mL/min was employed at 40 °C. Reactions using substrates **1**, **2**, and **9**–**20** were monitored by a fluorescence detection (excitation = 267 nm, emission = 330 nm), while those using substrates **3**–**8** were monitored by UV absorbance at 254 nm.

General Enzymatic modular synthesis procedures

Module N1 (β1-6GlcNAcylation by human GCNT1): the reaction system contains MES buffer (100 mM, pH 7.0), an acceptor (2–10 mM), 2 equivalents of donor UDP-GlcNAc, $MgCl_2$ (10 mM), and 0.05 mg/mL of purified GCNT1.

Module N2 (β1-3GlcNAcylation by HpLgtA): the reaction system contains MES buffer (100 mM, pH 7.0), an acceptor (2–10 mM), 2 equivalents of donor UDP-GlcNAc, $MgCl_2$ (10 mM), and 0.2 mg/mL of purified HpLgtA.

Module G (β1-4galactosylation by HpLgtB): the reaction system contains MES buffer (100 mM, pH 7.0), an acceptor (2–10 mM), 2 equivalents of donor UDP-Gal, $MgCl_2$ (10 mM), and 0.4 mg/mL of purified HpLgtB.

Module F1 (α1-3fucosylation by Hp3FT): the reaction system contains MES buffer (100 mM, pH 7.0), an acceptor (2-10 mM), 2 equivalents of donor GDP-Fuc, $MgCl_2$ (10 mM), and 0.2 mg/mL of purified Hp3FT.

Module F2 (α1-2fucosylation by Hm2FT): the reaction system contains MES buffer (100 mM, pH 7.0), an acceptor (2-10 mM), 2 equivalents of donor GDP-Fuc, $MgCl_2$ (10 mM), and 0.2 mg/mL of purified Hm2FT.

Module S1 (α2-3sialylation by ST3Gal4): the reaction system contains Tris-Cl buffer (100 mM, pH 7.5), an acceptor (2-10 mM), 2 equivalents of sugar donor CMP-Neu5Ac, $MgCl_2$ (10 mM), and 0.05 mg/mL of purified ST3Gal4.

Module S2 (α2-3sialylation by ST3Gal1): the reaction system contains Tris-Cl buffer (100 mM, pH 7.5), an acceptor (2-10 mM), 2 equivalents of sugar donor CMP-Neu5Ac, $MgCl_2$ (10 mM), and 0.05 mg/mL of purified ST3Gal1.

Module S3 (α2-6sialylation by Pd26ST): the reaction system contains Tris-Cl buffer (100 mM, pH 7.5), an acceptor (2-10 mM), 2 equivalents of sugar donor CMP-Neu5Ac, $MgCl_2$ (10 mM), and 0.05 mg/mL of purified ST3Gal1.

Module Su2 (6-O-sulfation by CHST2): the reaction system contains MES buffer (100 mM, pH 6.5), an acceptor (5-10 mM), PAPS (20 mM), $MgCl_2$ (10 mM), ATP (1 mM) and 0.1 mg/mL of purified CHST2.

Module Su6 (6-O-sulfation by CHST6): the reaction system contains MES buffer (100 mM, pH 6.5), an acceptor (5-10 mM), PAPS (20 mM), $MgCl_2$ (10 mM), ATP (1 mM) and 0.05 mg/mL of purified CHST6.

Reactions were carried out at 37 °C for 1 h to overnight and monitored by HPLC until conversion exceeded 90%. Reactions were quenched with an equal volume of ice-cold ethanol, stored at −20 °C for 30 min. Precipitates were removed by centrifugation, and the supernatants were concentrated by rotary evaporation. Products were subsequently purified by reverse-phase (RP) chromatography (ODS-SM C18

column, Yamazen) and P2/P4 gel filtration, pooled, and lyophilized for HRMS and NMR analysis (Supporting Information).

Glycopeptide synthesis and purification

Solid-phase peptide synthesis (SPPS) of GlyCAM-1 glycopeptide 49 was performed using a CEM Liberty Blue microwave peptide synthesizer. Fmoc-Thr-Wang resin (0.1 mmol, 0.76 mmol/g) served as the starting material. Fmoc-Thr(Ac_4Galβ1-3Ac_3GalNAc)–OH was incorporated as the eighth amino acid from the C-terminus. The crude peptide after cleavage and global deprotection was dissolved in H_2O (1 mg/mL) containing 5% hydrazine and stirred at room temperature for 3 h to remove O-acetyl protecting groups. The product was purified by RP HPLC and characterized by analytical HPLC and LC–MS. Preparative HPLC was performed on an XBridge Peptide BEH C18 column (130 Å, 5 mm, 10 × 250 mm) using solvent A (water with 0.1% TFA) and solvent B (acetonitrile with 0.1% TFA) with a linear gradient of 5–20% B over 30 min at 4 mL/min.

II. Supplementary Figure and Table

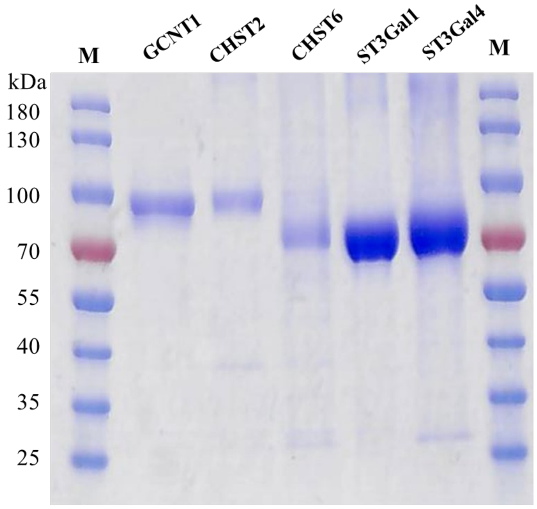

Figure S1. SDS-PAGE of purified human GCNT1 (74.1 kDa); CHST2 (77.8 kDa); CHST6 (69.4 kDa); ST3Gal1 (65.1 kDa); ST3Gal4 (63.9 kDa). The expression level of each enzyme: CHST2 (6.5 mg/100 mL), CHST6 (1.5 mg/100 mL), GCNT1 (1.8 mg/100 mL), ST3Gal1 (8 mg/100 mL), and ST3Gal4 (10 mg/100 mL).

Table S1. Relative activity of CHST2 and CHST6 towards different substrates

ACCEPTOR	RELATIVE ACTIVITY (nmol min^{-1} mg^{-1})			
	CHST2	STDEV	CHST6	STDEV
1	1.34	0.18	0.48	0.28
2	1.02	0.11	0.25	0.15
3	2.75	0.34	12.74	0.54
4	0	0	0	0
5	0.27	0.07	6.09	0.12
6	0.28	0.05	4.73	0.08
7	0.08	0.02	1.72	0.3
8	0.16	0.01	0.49	0.04
9	0	0	0	0
10	0	0	0	0
11	0.36	0.22	5.63	2.25
12	16.52	6.42	31.42	5.27
13	35.27	6.63	41.48	5.91
14	0.06	0.03	1.7	0.98
15	17.26	2.64	47.86	10.28
16	10.54	3.47	26.55	4.49
17	0.44	0.28	1.45	0.84
18	15.83	4.77	28.44	6.43
19	12.72	3.45	29.86	6.82
20	0.11	0.06	1.39	0.8

III. NMR and Mass Data of Synthesized Compounds

^{1}H,^{13}C NMR and 2-D NMR experiments were recorded on Bruker AVANCE 600 (600 MHz) or 900 (900 MHz) spectrometers at 25 °C. All ^{1}H Chemical shifts (in ppm) were assigned according to D_2O (δ= 4.79 ppm); HR-MS analyses were performed on a Waters Xevo G2_XS Mass Spectrometer (Waters Corporate, Milford, MA).

*NMR assignment and MS data of compound **21***

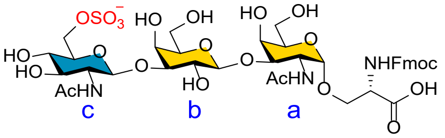

For GlcNAc-6-O-sulfate (c), additional resonances were assigned as c2 δ (3.66, 55.68), c3 δ (3.55, 74.80), and c4 δ (3.46, 69.59). An HMBC correlation between H-4 and C-6 confirmed assignment of the C-6 methylene at δ (4.18 – 4.26, 67.09). For comparison, the C-6 methylene of unsubstituted GlcNAc in compound **12** was observed at δ (3.63–3.69, 60.76), confirming the substantial downfield displacement associated with sulfation at this position.

Structural analysis of compound **21** followed the same strategy. Based on the assignments from compound 2-2, the anomeric signals of the three sugar residues were identified as a1 δ (4.79, 97.89), b1 δ (4.29, 104.59), and c1 δ (4.69, 102.68). Correlations of the glycosidic bonds Gal-1,3-GalNAc (b1–a3) and GlcNAc-1,3-Gal (c1–b3) in the HSQC–HMBC overlay (**Figure S2**) confirmed assignments of a3 δ (3.89, 77.25) and b3 δ (3.57, 82.17), thereby confirming the glycosidic connectivity. Additional resonances for GlcNAc-6-O-sulfate were assigned as c2 δ (3.77, 55.64), c3 δ (3.59, 73.55), and c4 δ (3.52, 69.59). The HMBC correlation between c4 and c6 δ (4.69, 82.02) verified that sulfation occurs at the C-6 position of GlcNAc (c).

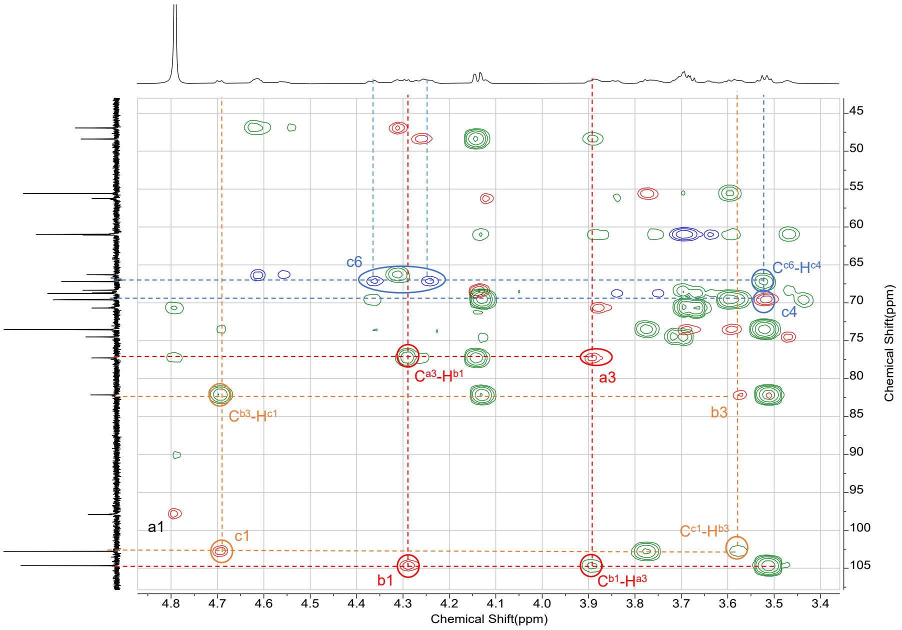

Fig.S2 Overlaid spectra of HSQC and HMBC for Compound **21**.

Chemical Shift (ppm)	Proton						
	1	2	3	4	5	6	NHAc
a – GalNAc	4.80	4.25	3.90	4.15	-[a]	3.91, 3.77	2.05
	97.91	48.42	77.37	68.63	-[a]	60.52	22.11
b – Gal	4.32	3.59	4.11	3.54	-[a]	3.69, 3.65	-[b]
	104.61	73.84	68.26	81.84	-[a]	60.89	-[b]
c – GlcNAc	4.69	3.74	3.49	3.59	-[a]	3.69, 3.65	2.05
	102.75	55.68	69.74	73.84	-[a]	60.89	22.11

[a] Not assigned. [b] Not applicable

	Chemical Shift (ppm)				
	Fmoc (Fluorenyl, 1-6)	Fmoc (Fluorenyl, 7)	Fmoc (CH_2, 8)	Serine (CH, 11)	Serine (CH2, 12)
Proton	7.57 – 7.10 (m, 8H)	4.33	4.61	4.19	3.84, 3.78
Carbon	176.06, 174.53, 174.23, 163.10, 162.87, 157.33, 143.60, 140.83, 127.89, 127.36, 124.91, 120.06	47.12	66.28	55.68	68.52

HRMS, $C_{40}H_{52}N_3O_{23}S$, Calcd for: 974.2712; found $[M-H]^-$ 974.2645.

NMR assignment and MS data of compound ***26***

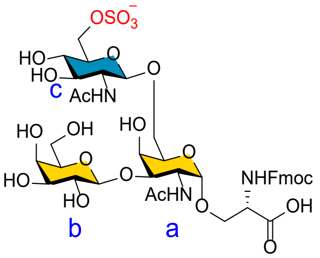

Assignment began with Fmoc-Serine, where the CH_2 (**12**) signals were identified at δ (3.76/3.72, 68.71). The anomeric signal of GalNAc (a1) was assigned at δ (4.76, 97.90) by correlation with Ser CH_2 (**12**) in the HMBC–HSQC overlay (**Figure S3**). Subsequent HSQC-TOCSY experiments enabled systematic assignment of GalNAc resonances at a2 δ (4.26, 48.33), a3 δ (3.90, 77.10), and a4 δ (4.10, 48.66), respectively (**Figure S4**).

Given that Gal (b) is linked to GalNAc through an α1→3 glycosidic bond, correlation between a3 and b1 confirmed the anomeric signal of Gal at δ (4.36, 104.63). Similarly, the anomeric signal of GlcNAc (c1) was assigned at δ (4.48, 101.50) (**Figure S3**). One-dimensional ^1H NMR provided coupling constants ($J_{H,H}$) of 3.0 Hz (GalNAc, a), 9.0 Hz (Gal, b), and 7.8 Hz (GlcNAc, c), consistent with an α-linkage between Ser and GalNAc, and β-linkages for both Gal-1,3-GalNAc and GlcNAc-1,6-GalNAc in compound **26**.

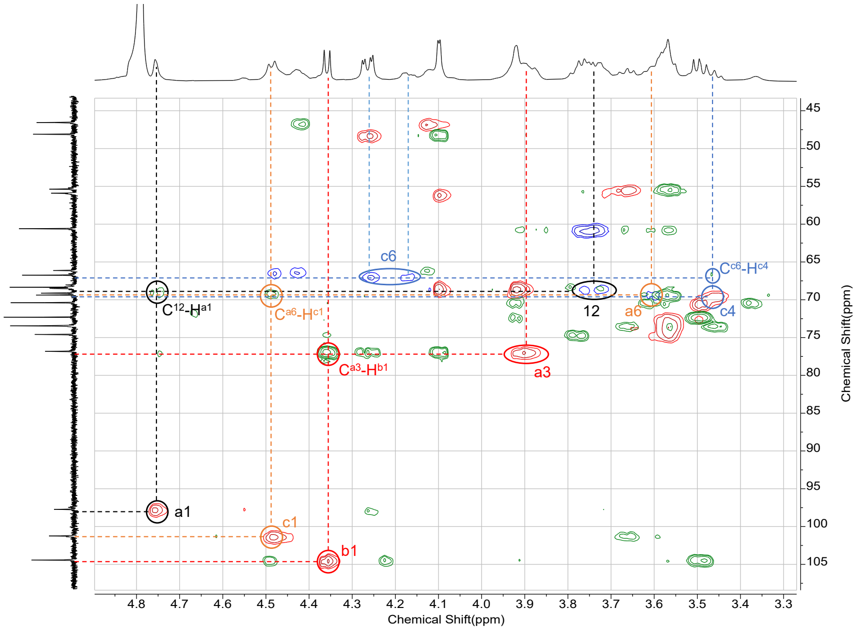

Fig.S3 Overlaid spectra of HSQC and HMBC for compound **26**

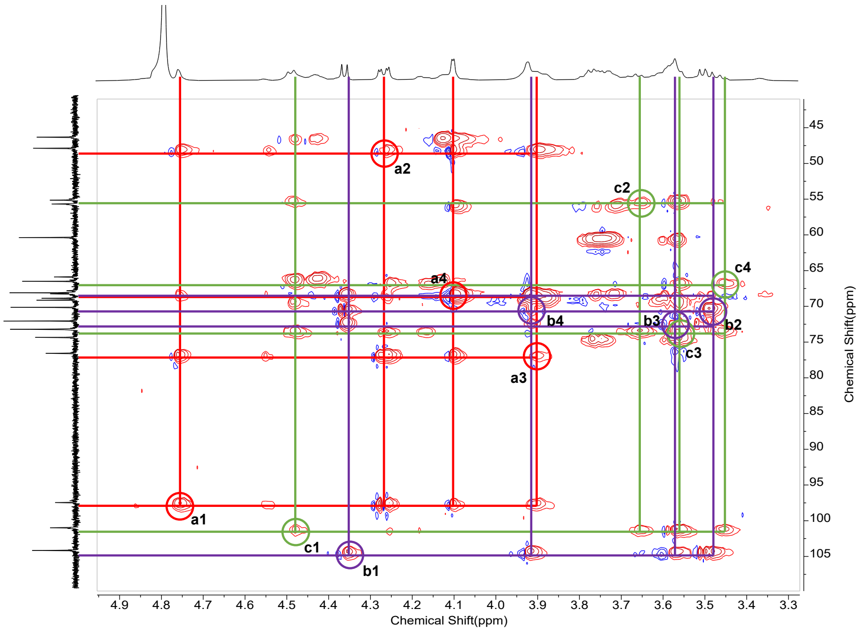

Fig. S4 HSQC-TOCSY of Compound26, showing the cross signals of 1-4 positions of each residue sugar.

Chemical Shift (ppm)	Proton						
	1	2	3	4	5	6	NHAc
a – GalNAc	4.76 (s)	4.21	3.84	4.05	-[a]	3.55	1.97
	97.83	48.36	77.04	68.57	-[a]	69.33	22.19
b – Gal	4.36 (d, J =	3.45	3.44	3.87	-[a]	3.76 – 3.64	-[b]

	7.7 Hz)						
	104.55	70.61	70.61	68.71	-[a]	60.84	-[b]
c – GlcNAc-6-Sulfate	4.48 (d, J = 9.0 Hz)	3.60	3.51	3.40	-[a]	4.23 – 4.09	1.97
	101.43	55.61	74.81	69.59	-[a]	66.96	22.19

[a] Not assigned. [b] Not applicable

	Chemical Shift (ppm)				
	Fmoc (Fluorenyl, 1-6)	Fmoc (Fluorenyl, 7)	Fmoc (CH_2, 8)	Serine (CH, 11)	Serine (CH2, 12)
Proton	7.74 – 7.36 (m, 8H)	4.13	4.48 –4.43	4.10	3.76 – 3.72
Carbon	143.86, 143.73, 140.95, 140.89, 127.99, 127.47, 125.00, 120.14	46.85	66.55	56.23	68.74

HRMS, $C_{40}H_{52}N_3O_{23}S$, Calcd for: 974.2712; found $[M-H]^-$ 974.2728

*NMR assignment and MS data of compound **38***

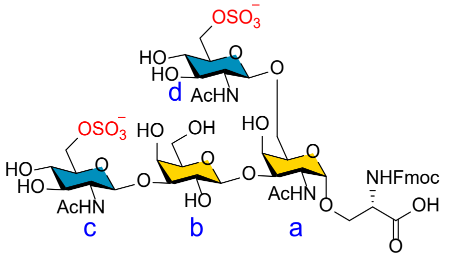

Chemical Shift (ppm)	Proton						
	1	2	3	4	5	6	NHAc
a – GalNAc	4.70	4.21	3.84	4.05	-[a]	3.84 – 3.63	1.91
	97.77	48.36	77.04	68.57	-[a]	70.21	22.19
b – Gal	4.20	3.45	3.44	3.87	-[a]	3.65	-[b]
	104.58	70.61	70.61	68.71	-[a]	60.92	-[b]
c – GlcNAc	4.59	3.75	3.55	3.46	-[a]	4.27 – 4.11	1.91
	102.75	55.68	74.10	69.82	-[a]	67.23	22.19
d – GlcNAc-6-Sulfate	4.43	3.65	3.52	3.43	-[a]	4.27 – 4.11	1.91
	101.76	55.68	74.81	69.81	-[a]	67.23	22.19

[a] Not assigned. [b] Not applicable

^{13}C NMR (151 MHz, D_2O) δ 174.91, 174.28, 173.36, 143.63, 140.89, 127.99, 127.45, 124.97, 120.14, 104.52, 102.71, 101.77, 97.92, 82.22, 77.03, 74.50, 73.77, 73.63, 73.48, 69.68, 69.52, 68.52, 68.26, 67.31, 67.12, 66.42, 60.93, 55.57, 54.08, 48.33, 46.80, 22.16, 22.00.

HRMS, $C_{48}H_{64}N_4O_{31}S_2$, Calcd for: 1256.2996; found $[M-2H]^{2-}$ 628.1477.

*NMR assignment and MS data of compound **43***

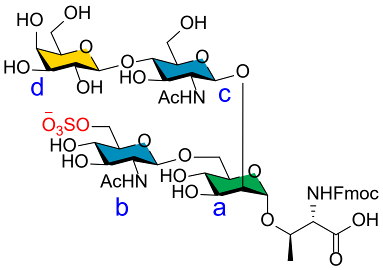

Chemical Shift (ppm)	Proton						
	1	2	3	4	5	6	NHAc
a – Man	4.76	3.72	3.73	-[a]	-[a]	4.19	-[b]
	98.10	69.11	71.92	-[a]	-[a]	69.95	-[b]
b – GlcNAc-6-Sulfate	4.59	3.77	3.61	3.55	3.72	4.38, 4.26	2.06
	101.31	55.49	73.71	69.66	78.27	67.08	22.39
c – GlcNAc	4.49	3.73	3.86	3.68	3.38	3.95, 3.84	2.06
	99.72	54.80	77.04	73.78	67.57	59.87	22.39
d – Gal	4.47	3.56	3.67	3.94	-[a]	3.80-3.74	-[b]
	102.88	70.92	72.46	68.55	-[a]	61.01	-[b]

[a] Not assigned. [b] Not applicable

	Chemical Shift (ppm)					
	Fmoc (Fluorenyl, 1-6)	Fmoc (Fluorenyl, 7)	Fmoc (CH_2, 8)	Threonine (CH, 11)	Threonine (CH, 12)	Threonine (CH_3, 13)
Proton	7.91 – 7.41 (8H)	4.25	4.80, 4.53	3.80	4.23	1.00
Carbon	143.99, 143.53, 141.06, 140.97, 128.13, 128.07, 127.54, 124.99, 124.81, 120.25, 120.20	47.24	65.87	60.53	76.87	18.46

HRMS, $C_{47}H_{64}N_3O_{28}S$, Calcd for: 1150.3397; found $[M-H]^-$ 1150.3322.

NMR and MS data of Synthesized Glycans

Compound 11

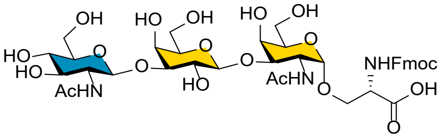

^{1}H NMR (600 MHz, D_2O) δ 7.89 – 7.84 (m, 2H), 7.69 (dd, *J* = 18.6, 7.5 Hz, 2H), 7.48 (q, *J* = 7.8 Hz, 2H), 7.44 – 7.38 (m, 2H), 4.80 (s, 1H), 4.69 (d, *J* = 8.4 Hz, 1H), 4.56 (dd, *J* = 10.5, 5.7 Hz, 1H), 4.53 – 4.48 (m, 1H), 4.33 – 4.21 (m, 3H), 4.16 – 4.08 (m, 3H), 3.95 – 3.66 (m, 12H), 3.54 – 3.48 (m, 5H), 2.20 –1.81 (m, 6H). ^{13}C NMR (151 MHz, D_2O) δ 176.19, 174.96, 174.54, 157.57, 143.86, 140.94, 128.03, 127.54, 127.50, 125.04, 120.19, 120.16, 104.68, 102.76, 97.93, 81.89, 77.27, 75.70, 74.34, 73.62, 70.67, 69.71, 69.68, 68.84. HRMS, $C_{40}H_{53}N_3O_{20}$, Calcd for: 895.3222; found $[M-H]^-$ 894.3101

Compound 12

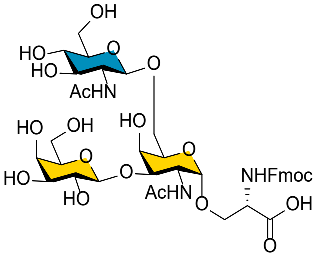

[1]NMR (600 MHz, D_2O) δ 7.57 – 7.10 (m, 8H), 4.67 (s, 1H), 4.37 (d, *J* = 8.3 Hz, 1H), 4.30 – 4.15 (m, 4H), 4.02 (s, 2H), 3.91 (s, 1H), 3.86 – 3.75 (m, 4H), 3.73 – 3.60 (m, 4H), 3.58 – 3.52 (m, 3H), 3.49 – 3.41 (m, 3H), 3.40 (dd, *J* = 9.9, 7.6 Hz, 1H), 3.34 – 3.23 (m, 2H), 2.07 – 1.77 (m, 6H). ^{13}C (151 MHz, D_2O) δ 176.06, 174.53, 174.23, 163.10, 162.87, 157.33, 143.60, 140.83, 127.89, 127.36, 124.91, 120.06, 117.32, 115.39, 104.63, 101.29, 97.94, 77.02, 75.80, 74.80, 73.78, 72.50, 70.59, 69.91, 69.28, 69.01, 68.74, 68.54, 66.42, 60.84, 60.67, 57.43, 56.05, 55.62, 48.35, 46.70, 22.28, 22.17, 16.81. HRMS, $C_{40}H_{53}N_3O_{20}$, Calcd for: 895.3222; found $[M-H]^-$ 894.3085

Compound 22

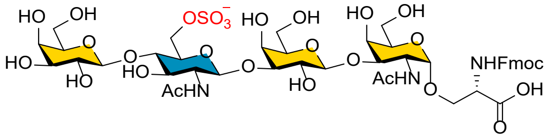

^{1}H NMR (900 MHz, D_2O) δ 7.93 (d, *J* = 7.7 Hz, 2H), 7.73 (d, *J* = 7.5 Hz, 2H), 7.54– 7.42 (m, 4H), 4.82 (s, 1H), 4.74 – 4.67 (m, 2H), 4.64 – 4.59 (m, 1H), 4.53 (d, *J* = 7.7 Hz, 1H), 4.49 (s, 1H), 4.44 (d, *J* = 11.0 Hz, 1H), 4.38 – 4.27 (m, 4H), 4.24 (d, *J* = 10.9 Hz, 1H), 4.17 – 4.13 (m, 2H), 3.93 (d, *J* = 3.4 Hz, 1H), 3.90 – 3.64 (m, 17H), 3.61 – 3.46 (m, 5H), 2.12 – 1.83 (m, 6H). ^{13}C NMR (226 MHz, D_2O) δ 174.85, 174.39, 173.49, 157.81, 143.76, 140.97, 128.11, 127.56, 125.01, 120.21, 104.60, 102.63, 98.11, 82.02, 77.71, 76.98, 75.35, 74.59, 72.48, 72.15, 70.98, 70.81, 69.54, 68.60, 68.52, 68.35, 67.67, 66.58, 66.34, 61.03, 55.22, 54.21, 48.43, 47.01, 22.18, 21.99. HRMS, $C_{46}H_{62}N_3O_{28}S$, Calcd for: 1136.3241; found $[M-H]^-$ 1136.3115

Compound 23

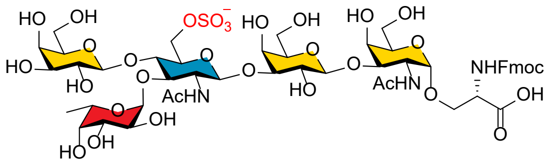

^{1}H NMR (900 MHz, D_2O) δ 7.97 – 7.87 (m, 2H), 7.77 – 7.64 (m, 2H), 7.55 – 7.39 (m, 4H), 5.14 (d, *J* = 4.2 Hz, 1H), 4.87 – 4.81 (m, 2H), 4.75 (d, *J* = 7.3 Hz, 2H), 4.69 – 4.60 (m, 1H), 4.56 – 4.49 (m, 1H), 4.43 – 4.28 (m, 5H), 4.27 – 4.20 (m, 1H), 4.15 (dd, *J* = 16.0, 3.2 Hz, 2H), 4.04 – 3.78 (m, 11H), 3.71 (m, 9H), 3.64 – 3.56 (m, 3H), 3.55 – 3.48 (m, 3H), 2.15 – 1.80 (m, 6H), 1.19 (d, *J* = 6.6 Hz, 3H). ^{13}C NMR (226 MHz, D_2O) δ 174.66, 143.79, 140.98, 128.10, 127.56, 125.02, 120.20, 118.27, 116.98, 115.69, 114.40, 104.62, 102.39, 101.59, 98.56, 98.05, 82.01, 77.01, 74.97, 74.66, 72.90, 72.44, 71.92, 71.01, 70.78, 69.59, 69.20, 68.58, 68.43, 68.32, 67.98, 67.76, 66.71, 66.33, 66.22, 61.46, 60.96, 55.90, 54.80, 48.43, 46.99, 22.25, 22.03, 15.32. HRMS, $C_{52}H_{72}N_3O_{32}S$, Calcd for: 1282.3820; found $[M-H]^-$ 1282.3705

Compound 24

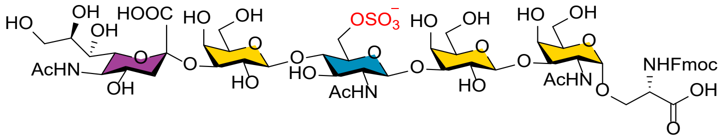

^{1}H NMR (600 MHz, D_2O) δ 7.95 (d, *J* = 7.6 Hz, 2H), 7.76 (s, 2H), 7.53 (d, *J* = 7.6 Hz, 2H), 7.46 (d, *J* = 6.9 Hz, 2H), 4.80 (1H), 4.72 (d, *J* = 8.1 Hz, 2H), 4.65 (s, 2H), 4.61 (d, *J* = 7.7 Hz, 1H), 4.45 (d, *J* = 11.2 Hz, 1H), 4.37 (s, 1H), 4.31 (d, *J* = 7.7 Hz, 2H), 4.29 – 4.19 (m, 2H), 4.18 – 4.12 (m, 3H), 3.98 (d, *J* = 3.1 Hz, 1H), 3.96 – 3.78 (m, 10H), 3.78 – 3.63 (m, 12H), 3.63 – 3.55 (m, 3H), 3.55 – 3.47 (m, 2H), 2.76 (dd, *J* = 12.5, 4.6 Hz, 1H), 2.07 – 1.92 (m, 9H), 1.82 (t, *J* = 12.1 Hz, 1H). ^{13}C NMR (226 MHz, D_2O) δ 175.60, 174.91, 174.50, 173.95, 163.08, 162.92, 157.69, 143.84, 140.99, 128.10, 127.55, 125.04, 120.22, 116.97, 115.68, 104.65, 103.47, 102.72, 102.48, 102.20, 100.12, 99.74, 97.95, 82.20, 77.39, 75.34, 75.08, 74.52, 72.85, 72.54, 72.42, 72.32, 72.11, 71.68, 71.52, 70.72, 69.52, 69.45, 68.65, 68.45, 68.31, 68.20, 68.08, 67.47, 66.96, 66.54, 66.30, 63.29, 62.65, 62.53, 61.04, 59.31, 55.78, 55.16, 54.88, 51.90, 51.70, 48.40, 46.97, 40.06, 39.57, 22.29, 22.18, 22.06. HRMS, $C_{57}H_{79}N_4O_{36}S$, Calcd for: 1427.4195; found $[M-H]^-$ 1427.4229

Compound 25

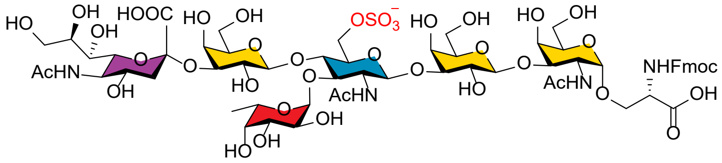

^{1}H NMR (600 MHz, D_2O) δ 7.94 – 7.84 (m, 2H), 7.74 – 7.60 (m, 2H), 7.49 (s, 2H), 7.42 (s, 2H), 5.09 (s, 1H), 4.78 (1H), 4.75 (d, *J* = 8.6 Hz, 2H), 4.69 (s, 3H), 4.63 – 4.54 (m, 2H), 4.34 (d, *J* = 19.3 Hz, 4H), 4.26 (d, *J* = 7.7 Hz, 1H), 4.11 (d, *J* = 10.0 Hz, 3H), 3.99 (d, *J* = 9.1 Hz, 1H), 3.94 – 3.73 (m, 14H), 3.72 – 3.60 (m, 12H), 3.59 – 3.52 (m, 4H), 3.49 (d, *J* = 7.7 Hz, 3H), 2.73 (d, *J* = 12.5 Hz, 1H), 2.01 – 1.88 (m, 9H), 1.81 (t, *J* = 12.3 Hz, 1H), 1.14 (d, *J* = 6.4 Hz, 3H). ^{13}C NMR (226 MHz, D_2O) δ 174.95, 174.65, 174.53, 174.02, 143.89, 128.11, 127.57, 125.06, 120.22, 118.27, 116.98, 115.69, 114.40, 104.67, 102.47, 101.24,

99.60, 98.52, 97.93, 75.42, 74.83, 74.55, 72.88, 72.73, 71.91, 71.41, 70.71, 69.58, 69.42, 69.17, 68.65, 68.43, 68.07, 67.86, 67.76, 67.27, 66.69, 66.30, 66.13, 62.55, 61.49, 60.96, 56.03, 51.74, 48.42, 46.98, 39.71, 22.25, 22.05, 19.80, 15.29. HRMS, $C_{63}H_{89}N_4O_{40}S$, Calcd for: 1537.4774; found $[M-2H]^{2-}$ 786.2420

Compound 27

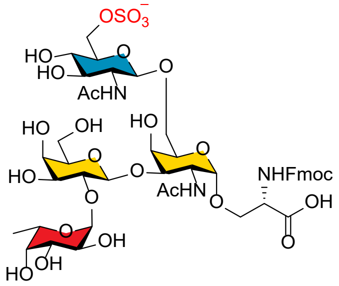

^{1}H NMR (900 MHz, D_2O) δ 7.97 – 7.88 (m, 2H), 7.78 – 7.65 (m, 2H), 7.58 – 7.46 (m, 2H), 7.46 (q, *J* = 8.2 Hz, 2H), 5.20 (d, *J* = 4.1 Hz, 1H), 4.77 (1H), 4.71 (dd, *J* = 11.0, 5.3 Hz, 1H), 4.65 – 4.59 (m, 1H), 4.52 (m, 2H), 4.36 (t, *J* = 5.3 Hz, 1H), 4.31 – 4.27 (m, 1H), 4.21 – 4.13 (m, 3H), 4.10 – 4.06 (m, 2H), 4.02 (dd, *J* = 11.1, 3.1 Hz, 1H), 3.99 – 3.95 (m, 1H), 3.93 (dd, *J* = 7.9, 3.4 Hz, 2H), 3.84 – 3.71 (m, 7H), 3.68 – 3.58 (m, 7H), 3.54 (dd, *J* = 10.9, 8.6 Hz, 1H), 3.42 (t, *J* = 9.5 Hz, 1H), 2.12 – 1.76 (m, 6H), 1.15 (dd, *J* = 32.1, 6.6 Hz, 3H). ^{13}C NMR (226 MHz, D_2O) δ 175.41, 174.27, 173.70, 157.57, 143.89, 141.02, 140.95, 128.03, 127.58, 125.04, 120.18, 101.97, 101.47, 99.29, 97.33, 76.56, 74.82, 73.82, 73.67, 73.51, 71.79, 69.90, 69.66, 69.53, 68.94, 68.29, 68.17, 67.06, 66.83, 66.19, 60.76, 55.65, 55.48, 49.20, 47.04, 22.18, 21.94, 15.36. HRMS, $C_{46}H_{62}N_3O_{27}S$, Calcd for: 1120.3291; found $[M-H]^-$ 1120.3213

Compound 28

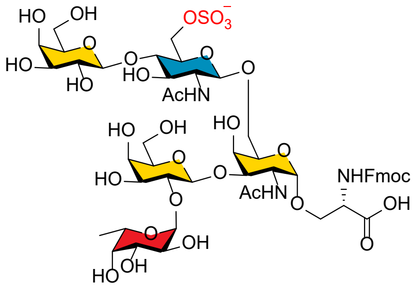

^{1}H NMR (900 MHz, D_2O) δ 7.97 – 7.86 (m, 2H), 7.78 – 7.63 (m, 2H), 7.56 – 7.40 (m, 4H), 5.20 (d, *J* = 4.2 Hz, 1H), 4.81 (1H), 4.74 – 4.67 (m, 1H), 4.62 (dd, *J* = 11.2, 5.5 Hz, 1H), 4.59 – 4.51 (m, 3H), 4.41 (d, *J* = 7.8 Hz, 1H), 4.36 (q, *J* = 8.7 Hz, 2H), 4.29 (s, 1H), 4.26 – 4.21 (m, 1H), 4.14 (d, *J* = 6.6 Hz, 1H), 4.11 – 4.04 (m, 3H), 4.01 (s, 2H), 3.96 – 3.87 (m, 4H), 3.84 – 3.58 (m, 22H), 3.51 (t, *J* = 9.0 Hz, 2H), 2.11 – 1.76 (m, 6H), 1.13 (d, *J* = 6.6 Hz, 3H). ^{13}C NMR (226 MHz, D_2O) δ 174.19, 173.70, 157.64, 143.91, 143.83, 141.04, 140.93, 128.05, 127.63, 125.08, 125.03, 120.26, 120.19, 116.98, 115.69, 102.62, 102.00, 101.47, 99.30, 97.27, 77.82, 76.64, 75.29, 74.82, 74.02, 73.46, 72.52, 72.46, 72.40, 71.77, 70.90, 70.12, 69.70, 69.55, 68.92, 68.85, 68.56, 68.19, 67.82, 66.87, 66.41, 66.27, 60.94, 60.77, 55.19, 54.77, 49.21, 47.09, 22.20, 21.87, 15.38. HRMS, $C_{52}H_{72}N_3O_{32}S$, Calcd for: 1282.3820; found $[M-H]^-$ 1282.3867

Compound 29

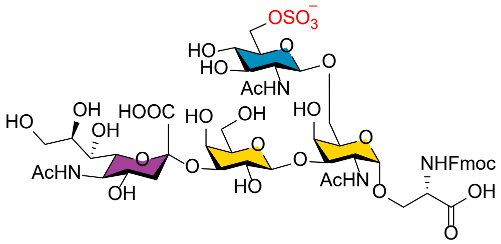

^{1}H NMR (600 MHz, D2O) δ 7.94 – 7.40 (m, 9H), 4.70 – 4.57 (m, 2H), 4.50 (d, *J* = 8.3 Hz, 1H), 4.46 (d, *J* = 7.7 Hz, 1H), 4.36 – 4.26 (m, 3H), 4.24 – 4.10 (m, 4H), 4.08 – 4.04 (m, 1H), 4.01 – 3.83 (m, 9H), 3.82 – 3.75 (m, 2H), 3.75 – 3.57 (m, 14H), 3.56 – 3.41 (m, 4H), 3.41 – 3.37 (m, 1H), 2.76 (d, *J* = 12.2 Hz, 1H), 2.05 – 1.91 (m, 9H), 1.81 (t, *J* = 11.9 Hz, 1H). HRMS, $C_{51}H_{69}N_4O_{31}S$, Calcd for: 1265.3666; found $[M-H]^-$ 1265.3613.

Compound 30

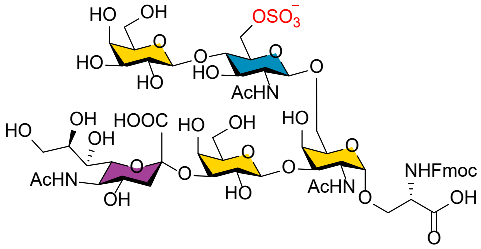

^{1}H NMR (600 MHz, D_2O) δ 7.88 (s, 2H), 7.77 – 7.57 (m, 2H), 7.45 (d, *J* = 39.1 Hz, 4H), 4.60 (s, 1H), 4.52 (d, *J* = 8.4 Hz, 1H), 4.47 (d, *J* = 7.9 Hz, 1H), 4.38 (d, *J* = 11.4 Hz, 3H), 4.30 (s, 1H), 4.23 (dd, *J* = 11.1, 5.3 Hz, 2H), 4.13 (d, *J* = 3.1 Hz, 1H), 4.09 (dd, *J* = 9.8, 3.4 Hz, 1H), 4.02 (s, 1H), 3.98 – 3.46 (m, 29H), 2.75 (d, *J* = 12.1 Hz, 1H), 2.06 – 1.85 (m, 10H). HRMS, $C_{57}H_{79}N_4O_{36}S$, Calcd for: 1427.4195; found $[M-2H]^{2-}$ 1427.4135.

Compound 31

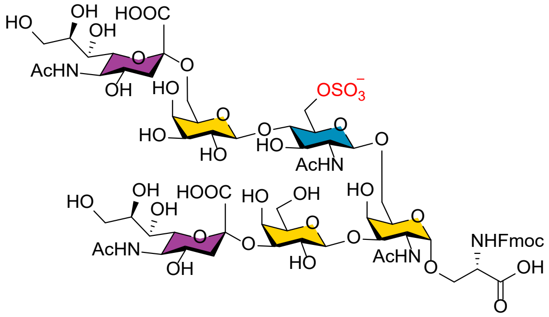

^{1}H NMR (900 MHz, D_2O) δ 7.96 (s, 2H), 7.79 – 7.65 (m, 2H), 7.57 – 7.42 (m, 5H), 4.82 (s, 2H), 4.72 – 4.60 (m, 1H), 4.57 (d, *J* = 7.7 Hz, 1H), 4.50 (d, *J* = 7.8 Hz, 1H), 4.38 (d, *J* = 10.9 Hz, 3H), 4.31 (d, *J* = 7.9 Hz, 1H), 4.25 – 4.14 (m, 3H), 4.08 (d, *J* = 11.6 Hz, 2H), 3.97 (s, 3H), 3.95 – 3.49 (m, 38H), 3.47 (d, *J* = 8.5 Hz, 1H), 2.77 (d, *J* = 13.4 Hz, 1H), 2.71 – 2.66 (m, 1H), 2.04 (d, *J* = 12.0 Hz, 6H), 2.14 – 1.83 (m, 6H), 1.81 (t, *J* = 12.4 Hz, 1H), 1.74 (t, *J* = 11.9 Hz, 1H). HRMS, $C_{68}H_{96}N_5O_{44}S$, Calcd for: 1718.5149; found $[M-2H]^{2-}$ 858.7537.

Compound 32

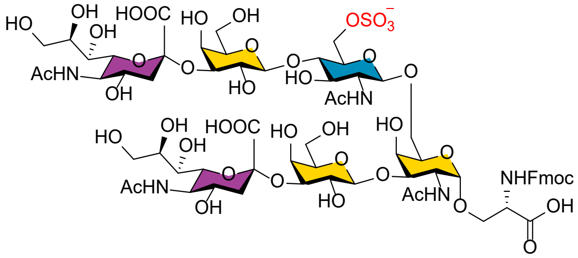

^{1}H NMR (600 MHz, D_2O) δ 7.86 (s, 2H), 7.74 – 7.55 (m, 2H), 7.52 – 7.33 (m, 4H), 4.58 (s, 2H), 4.52 – 4.40 (m, 3H), 4.40 – 4.20 (m, 5H), 4.20 – 4.04 (m, 5H), 4.01 – 3.42 (m, 38H), 2.72 (d, *J* = 12.7 Hz, 2H), 2.05 – 1.83 (m, 15H). HRMS, $C_{68}H_{96}N_5O_{44}S$, Calcd for: 1718.5149; found $[M-2H]^{2-}$ 858.7537.

Compound 33

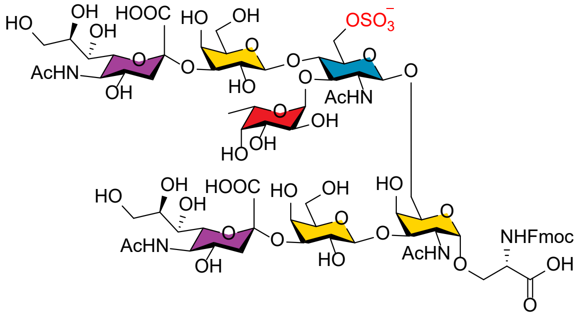

^{1}H NMR (600 MHz, D_2O) δ 7.98 – 7.88 (m, 2H), 7.79 – 7.64 (m, 2H), 7.55 – 7.44 (m, 4H), 5.07 (s, 1H), 4.66 (dd, *J* = 14.9, 5.6 Hz, 1H), 4.55 (d, *J* = 8.3 Hz, 2H), 4.46 (d, *J* = 8.0 Hz, 1H), 4.42 – 4.25 (m, 4H), 4.21 (d, *J* = 10.7 Hz, 1H), 4.15 – 4.03 (m, 3H), 4.00 – 3.81 (m, 17H), 3.81 – 3.58 (m, 21H), 3.57 – 3.45 (m, 4H), 2.75 (dd, *J* = 12.5, 4.9 Hz, 2H), 2.06 – 1.90 (m, 13H), 1.81 (t, *J* = 12.2 Hz, 2H), 1.16 (d, *J* = 6.4 Hz, 3H). HRMS, $C_{74}H_{106}N_5O_{48}S$, Calcd for: 1864.5728; found 931.8312 $[M-2H]^{2-}$

Compound 34

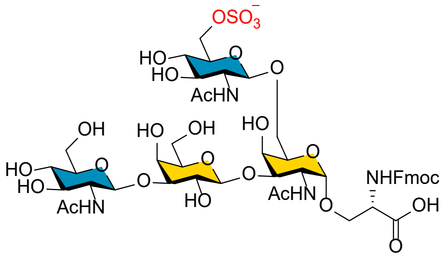

^{1}H NMR (600 MHz, D_2O) δ 7.95 – 7.38 (m, 8H), 4.69 (d, *J* = 8.4 Hz, 1H), 4.66 – 4.55 (m, 2H), 4.51 (d, *J* = 8.3 Hz, 1H), 4.32 (dd, *J* = 20.6, 7.1 Hz, 3H), 4.25 – 4.09 (m, 5H), 3.95 (d, *J* = 51.6 Hz, 4H), 3.76 (d, *J* = 9.6 Hz, 6H), 3.68 – 3.50 (m, 8H), 3.50 – 3.40 (m, 3H), 2.07 – 1.91 (m, 9H). HRMS, $C_{48}H_{65}N_4O_{28}S$, Calcd for: 1177.3506; found $[M-H]^-$ 1177.3456.

Compound 35

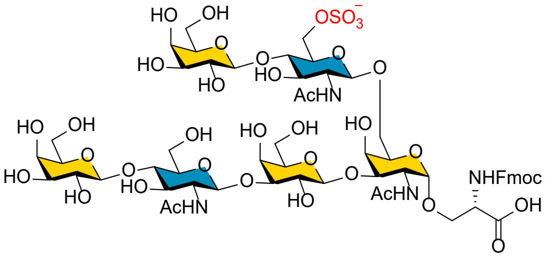

^{1}H NMR (600 MHz, D_2O) δ 7.93 – 7.34 (m, 8H), 4.76 (s, 1H), 4.66 (d, *J* = 7.5 Hz, 1H), 4.57 (dd, *J* = 10.5, 5.7 Hz, 1H), 4.49 (d, *J* = 8.4 Hz, 1H), 4.45 (d, *J* = 7.8 Hz, 1H), 4.40 – 4.25 (m, 5H), 4.24 – 4.15 (m, 2H), 4.11 (d, *J* = 15.6 Hz, 4H), 4.03 (d, *J* = 7.7 Hz, 1H), 3.97 – 3.44 (m, 37H), 3.36 (dd, *J* = 8.8, 6.3 Hz, 2H), 3.29 – 3.20 (m, 2H), 2.02 – 1.78(m, 9H). HRMS, $C_{60}H_{85}N_4O_{38}S$, Calcd for: 1501.4563; found $[M-H]^-$ 1501.4567

Compound 36

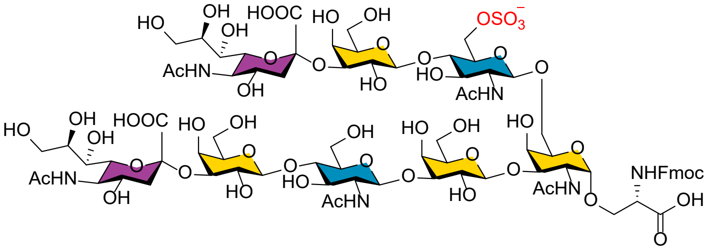

^{1}H NMR (600 MHz, D_2O) δ 7.91 (m, 2H), 7.69 (m, 2H), 7.47 (m, 4H), 4.79 (1H), 4.68 (1H), 4.61 (1H), 4.52 (m, 3H), 4.36 (m, 4H), 4.13 (m, 7H), 3.98 – 3.46 (m, 45H), 2.74 (q, *J* = 5.4 Hz, 2H), 2.12 – 1.71 (m, 9H), 1.87 (s, 2H). HRMS, $C_{82}H_{119}N_6O_{54}S$, Calcd for: 2083.6471; found $[M-2H]^{2-}$ 1041.3156

Compound 37

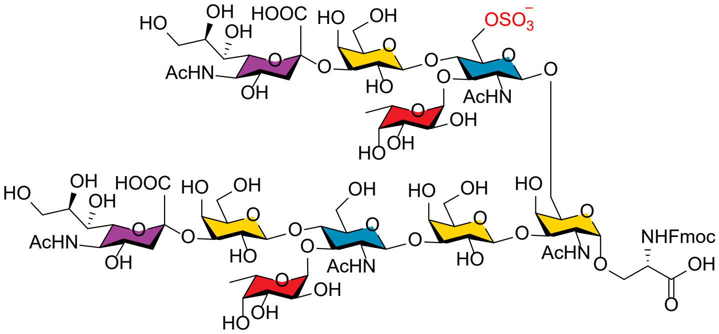

^{1}H NMR (600 MHz, D_2O) δ 7.94 (m, 2H), 7.76 (m, 2H), 7.52 (m, 2H), 7.45 (m, 2H), 5.11 (s, 1H), 5.06 (s, 1H), 4.81 – 4,78 (m, 2H), 4.75 (1H), 4.70 (1H), 4.61 (d, *J* = 37.4 Hz, 4H), 4.52 (s, 3H), 4.37 (dd, *J* = 13.7, 7.0 Hz, 3H), 4.24 (d, *J* = 17.6 Hz, 2H), 4.11 (d, *J* = 30.8 Hz, 5H), 4.02 – 3.80 (m, 24H), 3.79 – 3.45 (m, 43H), 2.75 (dd, *J* = 12.6, 6.3 Hz, 2H), 2.07 – 1.88 (m, 15H), 1.78 (d, *J* = 12.8 Hz, 2H), 1.19 – 1.10 (m, 6H). HRMS, $C_{94}H_{139}N_6O_{62}S$, Calcd for: 2375.7629; found $[M-2H]^{2-}$ 1187.3822

Compound 39

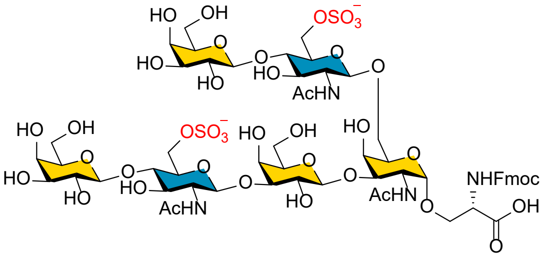

^{1}H NMR (600 MHz, D_2O) δ 7.90 (s, 2H), 7.73 (s, 2H), 7.47 (d, J = 40.3 Hz, 4H), 4.70 (s, 1H), 4.61 (s, 1H), 4.55 – 4.50 (m, 2H), 4.40 (d, J = 8.0 Hz, 3H), 4.37 – 4.30 (m, 4H), 4.25 (dd, J = 11.2, 5.1 Hz, 1H), 4.13 (d, J = 14.8 Hz, 2H), 4.03 (s, 1H), 3.95 – 3.48 (m, 33H), 2.06 – 1.92 (m, 9H). HRMS, $C_{60}H_{84}N_4O_{41}S_2$, Calcd for: 1580.4052; found $[M-2H]^{2-}$ 790.2045

Compound 40

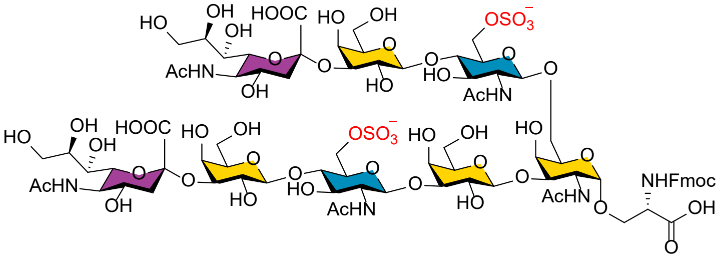

^{1}H NMR (600 MHz, D_2O) δ 7.94 (s, 2H), 7.71 (d, J = 50.9 Hz, 2H), 7.49 (d, J = 40.6 Hz, 4H), 4.70 (d, J = 8.1 Hz, 2H), 4.61 (d, J = 7.8 Hz, 2H), 4.53 (d, J = 8.3 Hz, 2H), 4.37 (s, 7H), 4.24 – 4.09 (m, 5H), 4.09 – 4.00 (m, 1H), 3.99 – 3.48 (m, 43H), 2.79 – 2.71 (m, 2H), 2.07 – 1.86 (m, 16H). HRMS, $C_{82}H_{118}N_6O_{57}S_2$, Calcd for: 2162.5961; found $[M-2H]^{2-}$ 1081.2894.

Compound 41

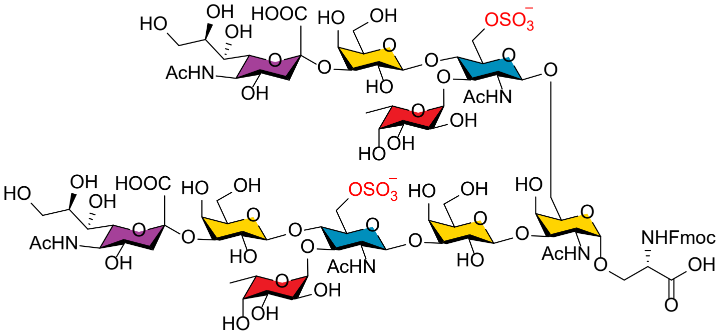

^{1}H NMR (900 MHz, D_2O) δ 7.98 – 7.90 (m, 2H), 7.73 (d, J = 82.0 Hz, 2H), 7.54 (s, 2H), 7.47 (d, J = 8.0 Hz, 2H), 5.13 (s, 1H), 5.09 (s, 1H), 4.69 – 4.63 (m, 1H), 4.62 (d, J = 7.5 Hz, 1H), 4.59 – 4.54 (m, 2H), 4.35 (dd, J = 70.0, 20.0 Hz, 8H), 4.23 (d, J = 9.1 Hz, 1H), 4.17 – 4.08 (m, 5H), 4.05 – 3.81 (m, 28H), 3.81 – 3.64 (m, 35H), 3.62 (d, J = 9.8 Hz, 5H), 3.59 – 3.49 (m, 13H), 2.76 (d, J = 11.6 Hz, 2H), 2.05 – 1.97 (m, 15H), 1.82 (t, J = 12.2 Hz, 2H), 1.21 – 1.15 (m, 8H). HRMS, $C_{94}H_{138}N_6O_{65}S_2$, Calcd for: 2454.7119; found $[M-2H]^{2-}$ 1227.8594.

Compound 42

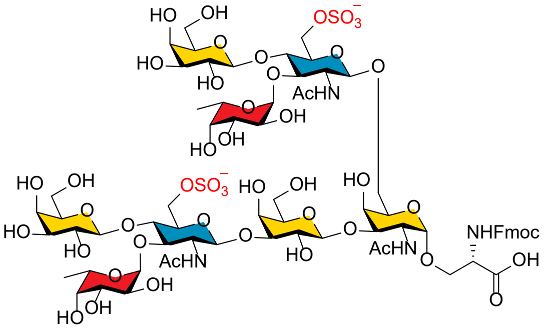

^{1}H NMR (600 MHz, D_2O) δ 7.83 (d, *J* = 22.3 Hz, 2H), 7.64 (d, *J* = 44.2 Hz, 2H), 7.48 – 7.32 (m, 4H), 5.05 (d, *J* = 4.0 Hz, 1H), 5.01 (s, 1H), 4.53 (d, *J* = 5.5 Hz, 2H), 4.45 (d, *J* = 7.5 Hz, 2H), 4.39 (d, *J* = 8.0 Hz, 1H), 4.27 (td, *J* = 21.1, 9.6 Hz, 6H), 4.14 (s, 2H), 4.07 (s, 1H), 4.03 (d, *J* = 3.1 Hz, 1H), 3.97 – 3.76 (m, 14H), 3.76 – 3.49 (m, 21H), 3.42 (m, 5H), 1.95 – 1.84 (m, 9H), 1.12 – 1.06 (m, 6H). HRMS, $C_{72}H_{104}N_4O_{49}S_2$, Calcd for: 1872.5211; found $[M-2H]^{2-}$ 936.2574.

Compound 44

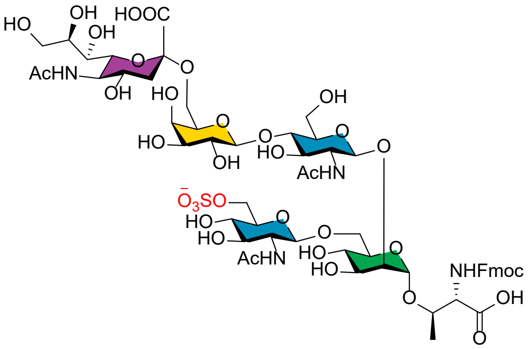

^{1}H NMR (600 MHz, D_2O) δ 7.96 (s, 2H), 7.77 (dd, *J* = 14.5, 7.8 Hz, 2H), 7.58 – 7.43 (m, 4H), 4.59 (d, *J* = 8.4 Hz, 1H), 4.48 (t, *J* = 7.8 Hz, 2H), 4.40 (d, *J* = 10.8 Hz, 2H), 4.34 – 4.17 (m, 4H), 4.13 – 4.03 (m, 2H), 4.00 – 3.63 (m, 21H), 3.58 (q, *J* = 9.3 Hz, 6H), 3.41 (t, *J* = 9.9 Hz, 1H), 2.73 – 2.66 (m, 1H), 2.13 – 2.02 (m, 9H), 1.83 (m, 1H), 1.09 (d, *J* = 6.4 Hz, 2H). HRMS, $C_{58}H_{81}N_4O_{36}S$, Calcd for: 1441.4351; found $[M-H]^{-}$ 1441.4434

Compound 45

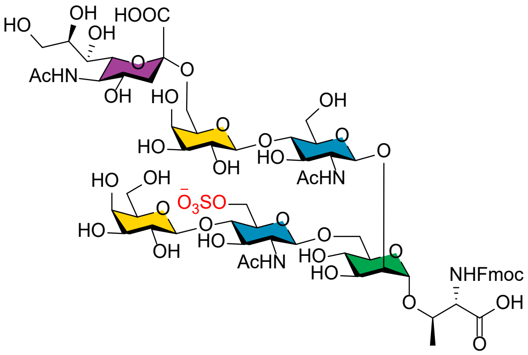

^{1}H NMR (600 MHz, D_2O) δ 7.93 (s, 2H), 7.77 – 7.63 (m, 2H), 7.55 – 7.39 (m, 4H), 4.76 (s, 1H), 4.59 (t, *J* = 9.4 Hz, 2H), 4.49 – 4.38 (m, 4H), 4.37 – 4.29 (m, 2H), 4.29 – 4.14 (m, 2H), 4.03 – 3.96 (m, 2H), 3.96 – 3.85 (m, 5H), 3.84 – 3.60 (m, 22H), 3.59 – 3.48 (m, 7H), 3.35 (t, *J* = 10.0 Hz, 1H), 2.67 (dd, *J* = 12.4, 4.6 Hz, 1H), 2.08 – 1.98 (m, 9H), 1.72 (t, *J* = 12.2 Hz, 1H), 1.03 (d, *J* = 6.1 Hz, 3H). HRMS, $C_{64}H_{91}N_4O_{41}S$, Calcd for: 1603.4879; found $[M-H]^-$ 1603.4882

Compound 46

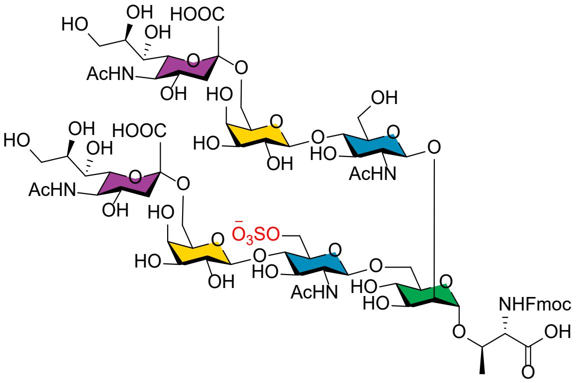

^{1}H NMR (600 MHz, D_2O) δ 7.97 (t, *J* = 7.5 Hz, 2H), 7.78 – 7.72 (m, 2H), 7.57 – 7.43 (m, 4H), 4.88 – 4.84 (m, 1H), 4.66 – 4.58 (m, 2H), 4.46 (dd, *J* = 22.8, 8.9 Hz, 3H), 4.42 – 4.33 (m, 2H), 4.33 – 4.18 (m, 3H), 4.05 – 3.51 (m, 42H), 3.39 – 3.32 (m, 1H), 2.73 – 2.65 (m, 2H), 2.11 – 1.99 (m, 12H), 1.76 – 1.70 (m, 2H), 1.08 (d, *J* = 6.4 Hz, 3H). HRMS, $C_{75}H_{108}N_5O_{49}S$, Calcd for: 1894.5834; found $[M-2H]^{2-}$ 946.7864.

Compound 47

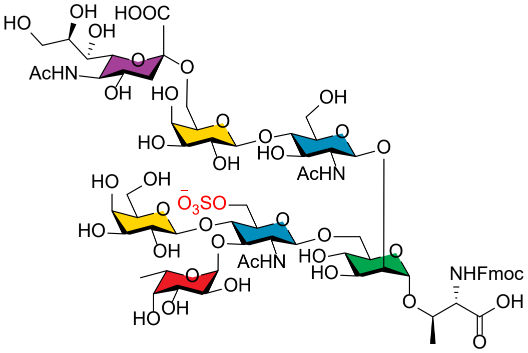

^{1}H NMR (600 MHz, D_2O) δ 7.95 (t, *J* = 6.5 Hz, 2H), 7.73 (td, *J* = 21.7, 7.3 Hz, 2H), 7.56 – 7.42 (m, 4H), 5.12 (d, *J* = 3.9 Hz, 1H), 4.90 – 4.82 (m, 2H), 4.75 (s, 1H), 4.62 (d, *J* = 4.8 Hz, 1H), 4.58 (dd, *J* = 10.9, 4.6 Hz, 1H), 4.54 (d, *J* = 8.0 Hz, 1H), 4.48 (d, *J* = 8.2 Hz, 1H), 4.43 (d, *J* = 7.9 Hz, 1H), 4.38 (dd, *J* = 14.9, 4.0 Hz, 3H), 4.26 – 4.20 (m, 2H), 3.99 (t, *J* = 9.4 Hz, 2H), 3.96 – 3.86 (m, 8H), 3.86 – 3.78 (m, 7H), 3.77 – 3.62 (m, 14H), 3.60 – 3.48 (m, 7H), 3.38 – 3.33 (m, 1H), 2.68 (dd, *J* = 12.4, 4.7 Hz, 1H), 2.11 – 1.92 (m, 9H), 1.72 (t, *J* = 12.2 Hz, 1H), 1.19 (d, *J* = 6.5 Hz, 3H), 1.03 (d, *J* = 6.6 Hz, 3H). HRMS, $C_{70}H_{101}N_4O_{45}S$, Calcd for: 1749.5459; found $[M-H]^-$ 1749.5597.

Compound 48

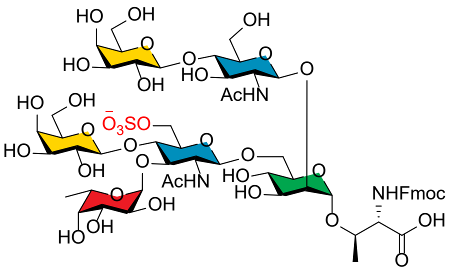

^{1}H NMR (600 MHz, D_2O) δ 7.97 (t, *J* = 7.3 Hz, 2H), 7.77 (dd, *J* = 23.2, 7.5 Hz, 2H), 7.55 (td, *J* = 7.6, 4.1 Hz, 2H), 7.54 – 7.42 (m, 2H), 5.14 (d, *J* = 4.1 Hz, 1H), 4.92 – 4.85 (m, 4H), 4.65 (d, *J* = 6.8 Hz, 1H), 4.61 – 4.54 (m, 2H), 4.49 (t, *J* = 8.5 Hz, 2H), 4.43 – 4.35 (m, 3H), 4.29 – 4.18 (m, 2H), 4.06 – 3.90 (m, 7H), 3.89 – 3.66 (m, 20H), 3.63 – 3.50 (m, 5H), 3.38 (t, *J* = 9.8 Hz, 1H), 2.11 – 2.01 (m, 6H), 1.21 (d, *J* = 6.5 Hz, 3H), 1.05 (d, *J* = 6.4 Hz, 3H). HRMS, $C_{59}H_{84}N_3O_{37}S$, Calcd for: 1458.4504; found $[M-H]^-$ 1458.4540.

IV. HPLC analysis of purified compounds.

The purity of synthesized compounds was analyzed by analytical HPLC equipped with an XBridge peptide BEH C18 column (130Å, 5 µm, 4.6×250 mm) and a UV detector at 210 and 254 nm. The running solvents are solvent A (H_2O with 0.1% TFA) and solvent B (Acetonitrile with 0.1% TFA), with a total flow rate of 1 mL/min at 40 °C. A linear gradient of 15-30% B over 20 min was used for **Compound 11, 12, 21-48**, and a linear gradient of 5-20% B over 20 min was used for glycopeptide (**Compound 49-54**).

Compound 11

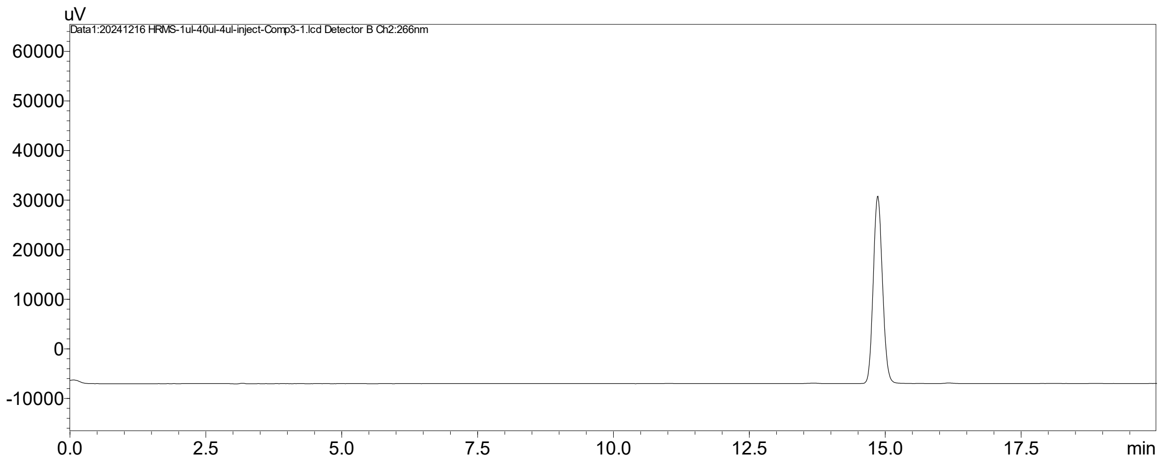

Compound 12

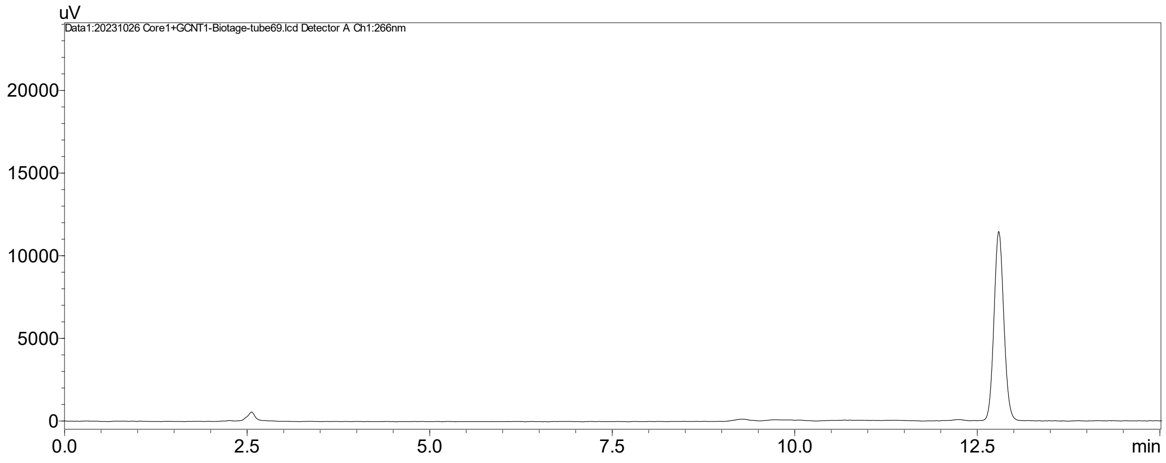

Compound 21

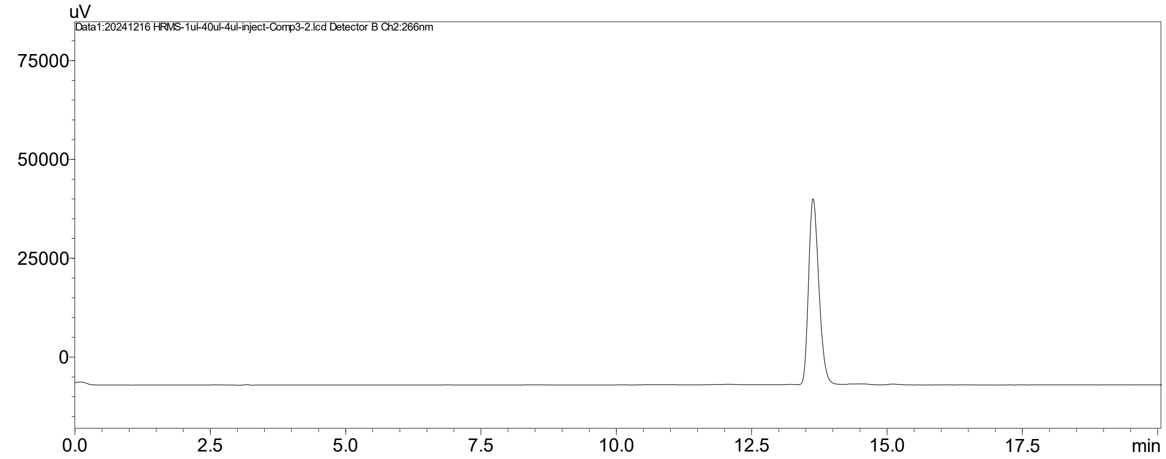

Compound 22

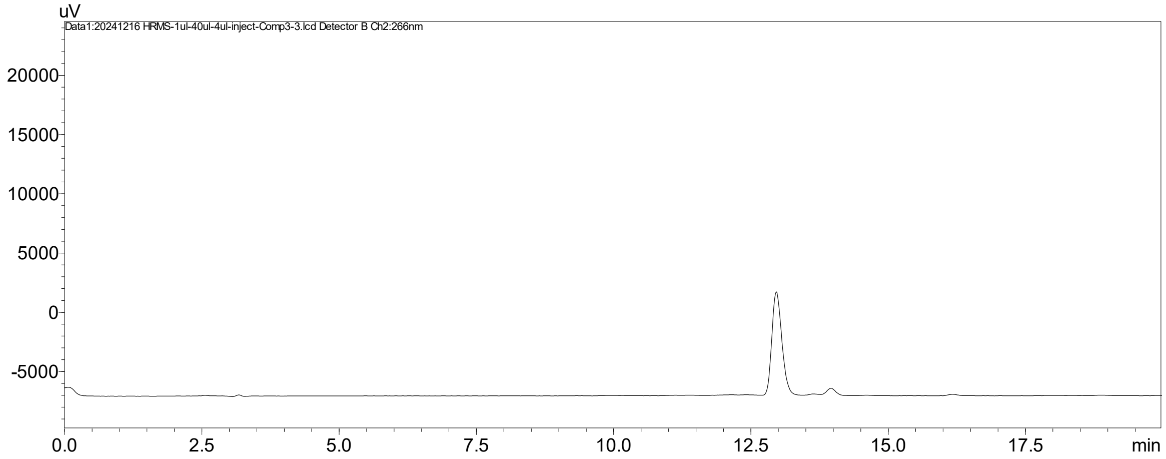

Compound 23

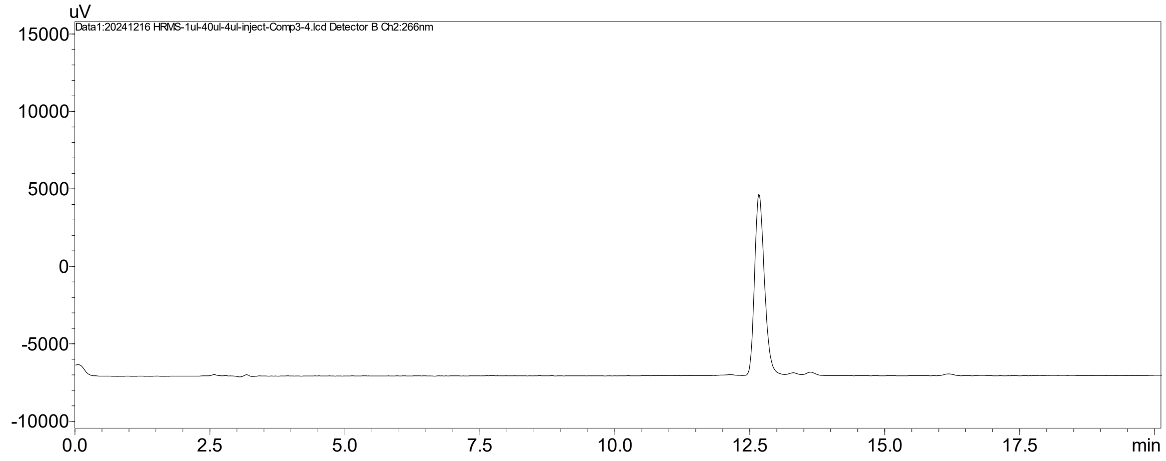

Compound 24

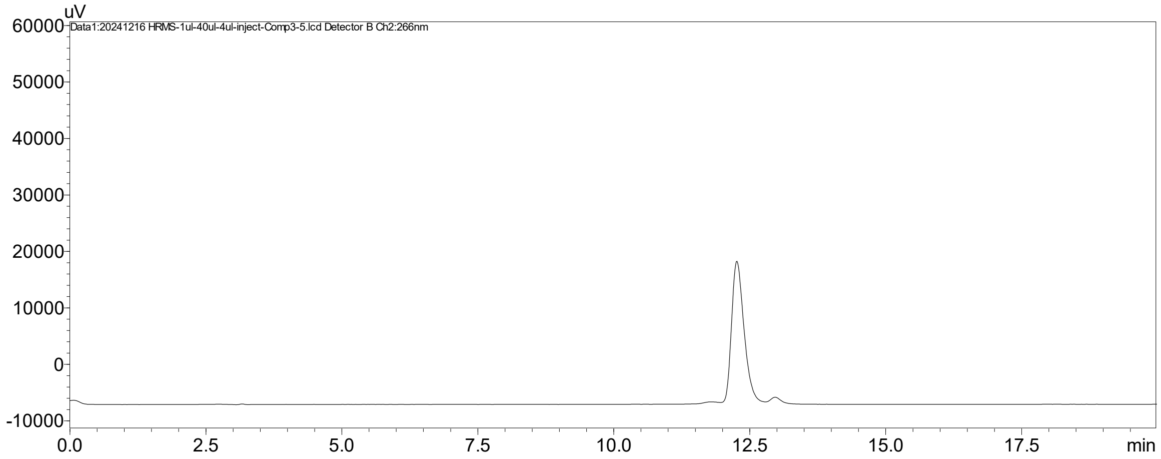

Compound 25

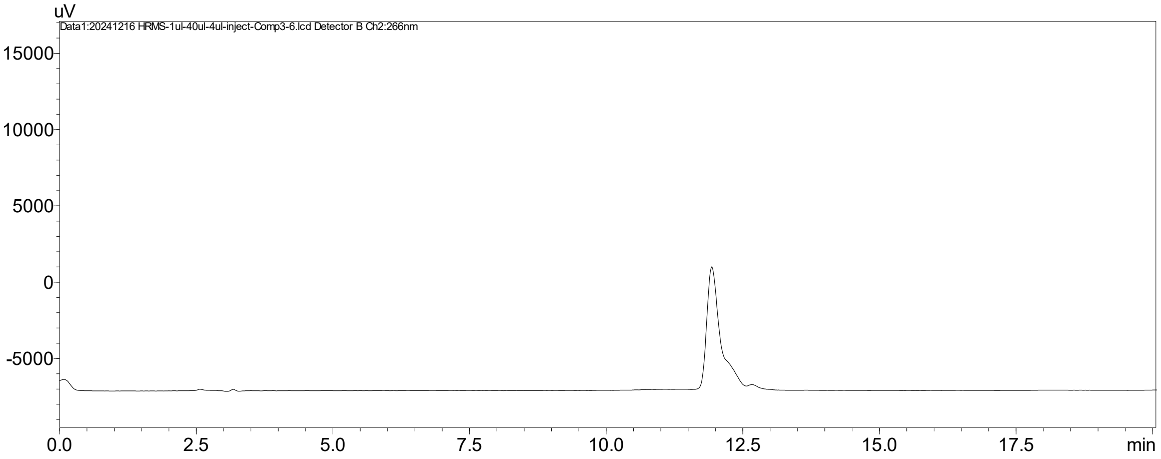

Compound 26

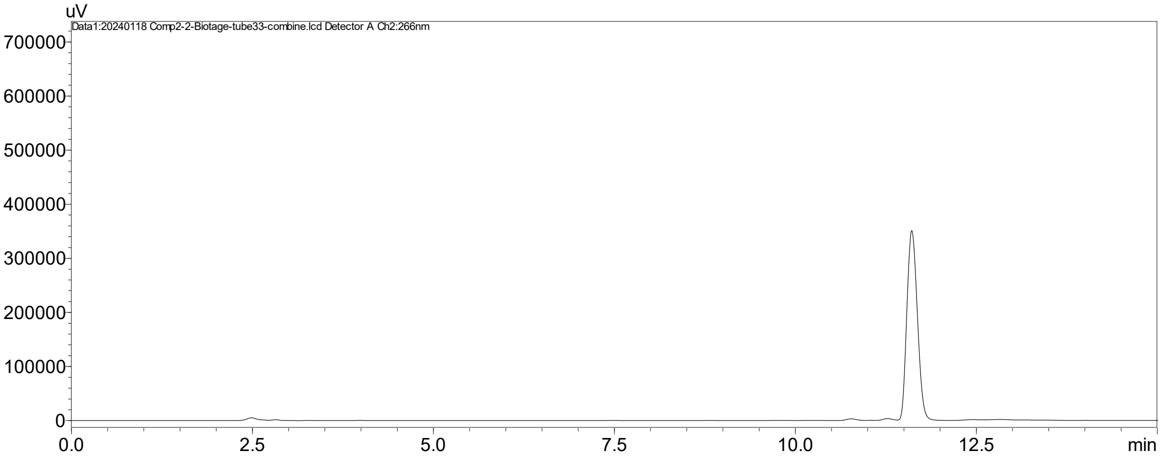

Compound 27

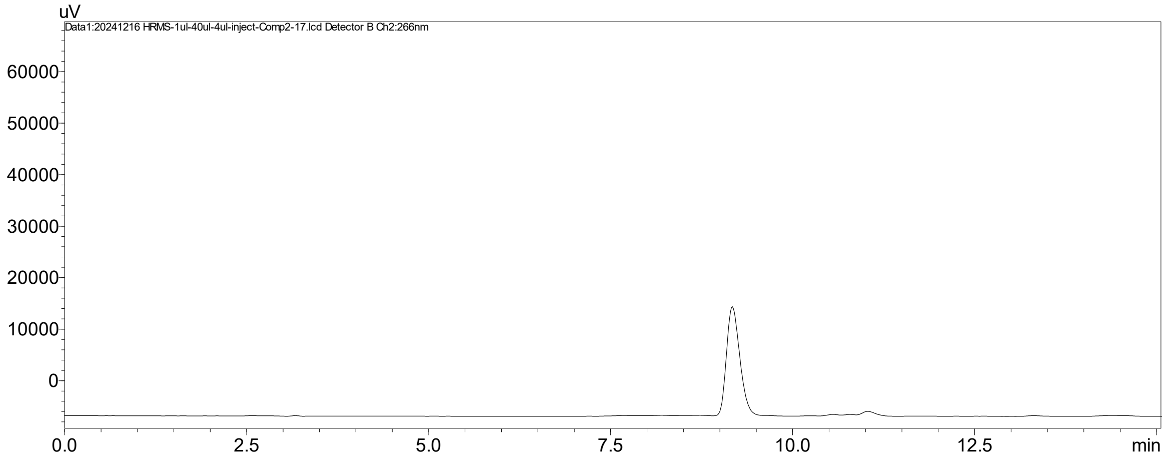

Compound 28

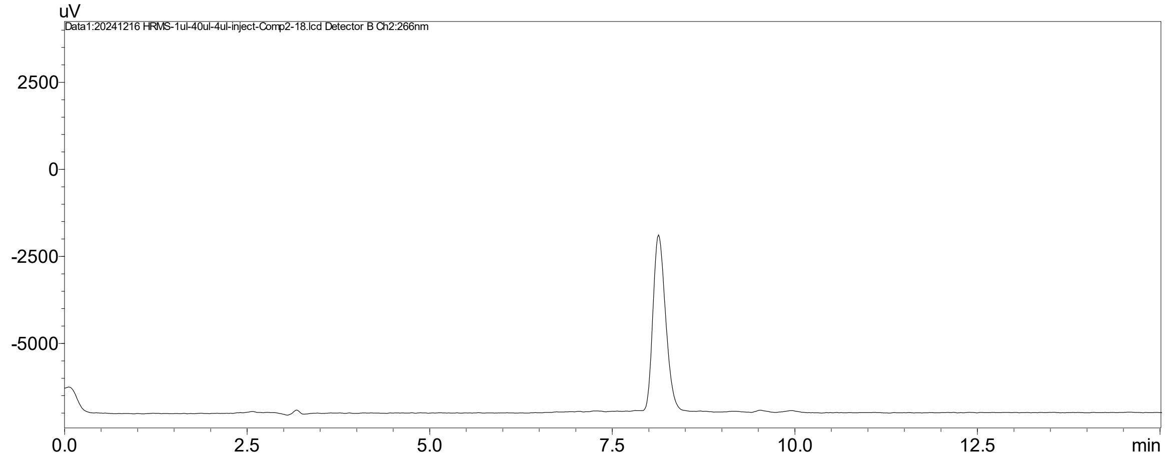

Compound 29

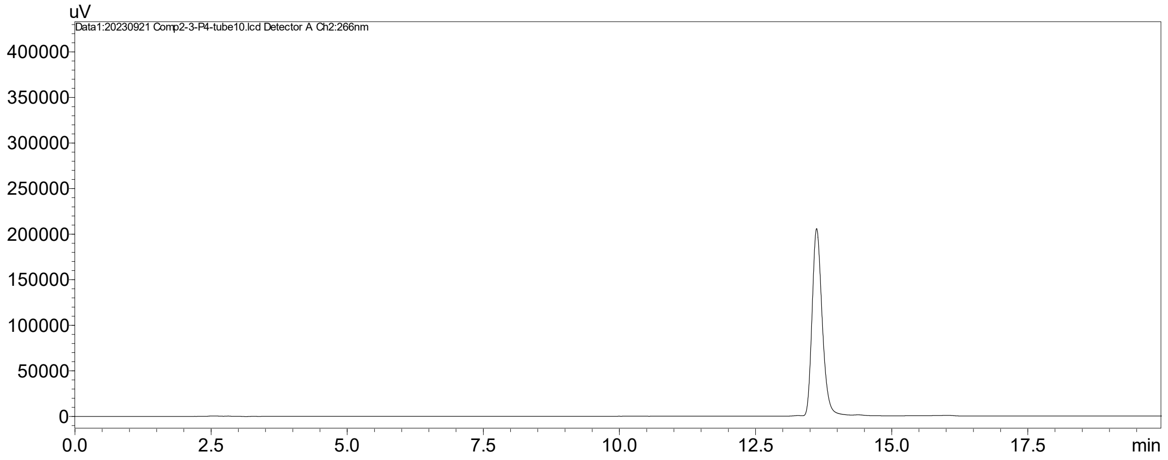

Compound 30

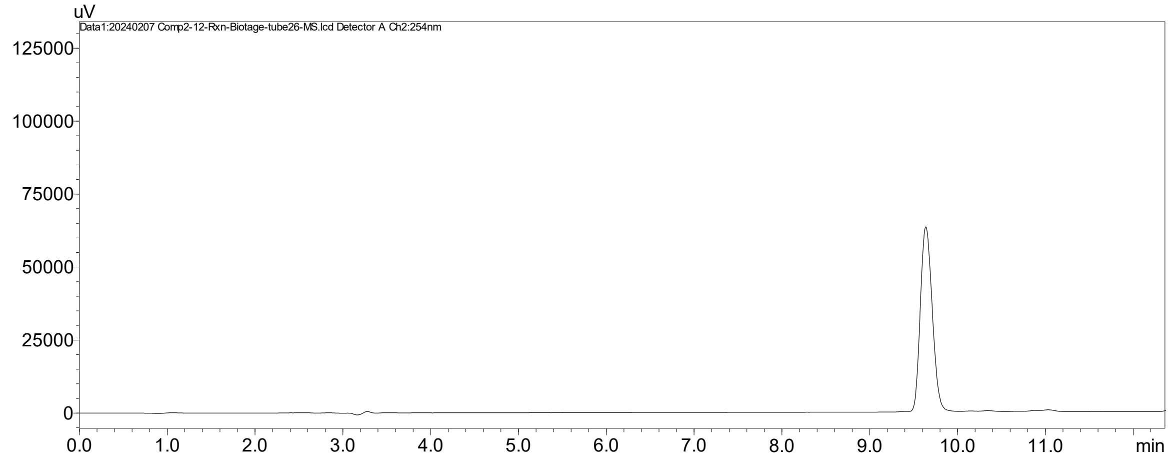

Compound 31

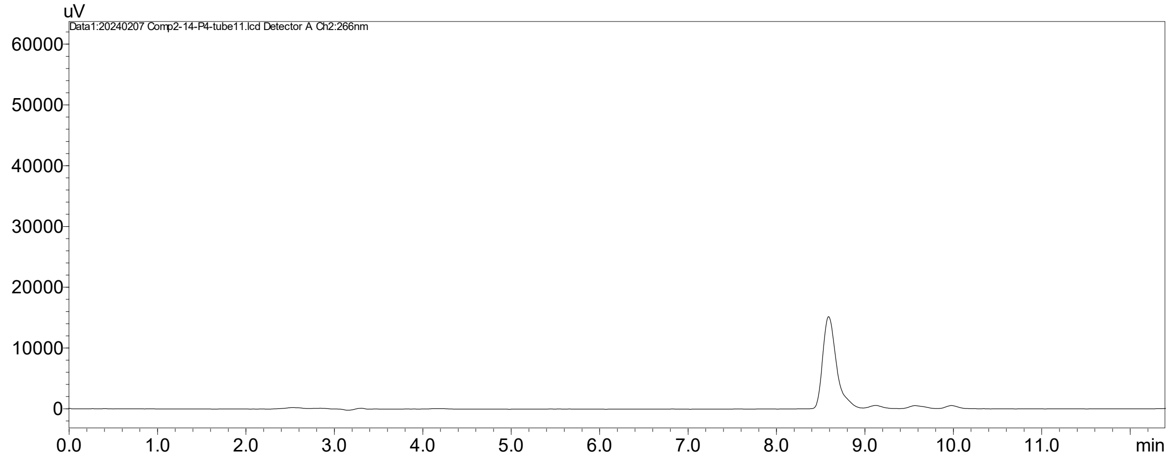

Compound 32

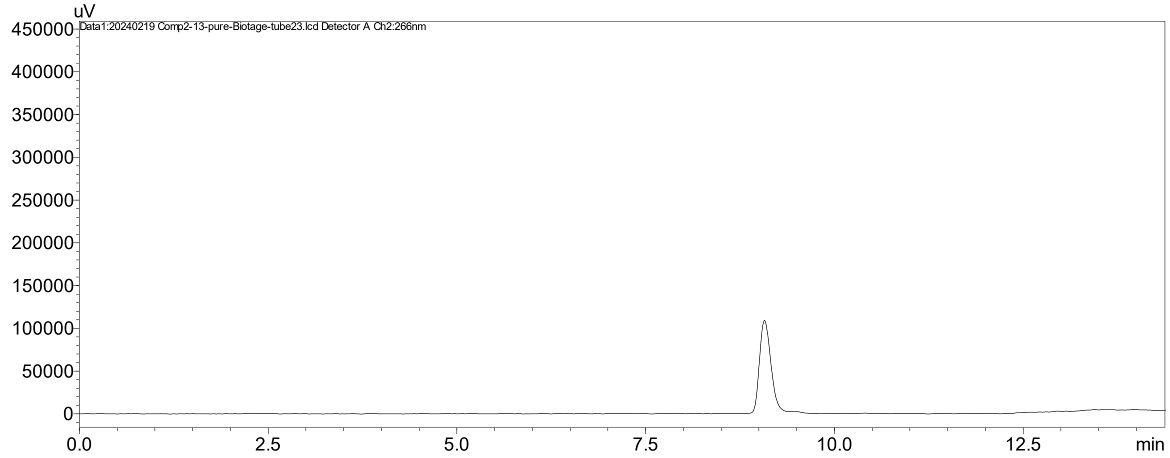

Compound 33

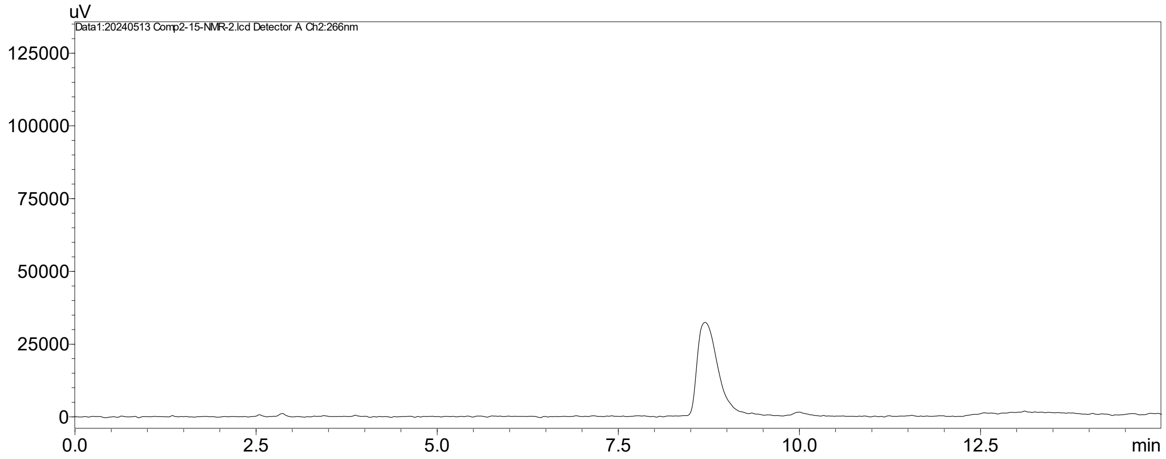

Compound 34

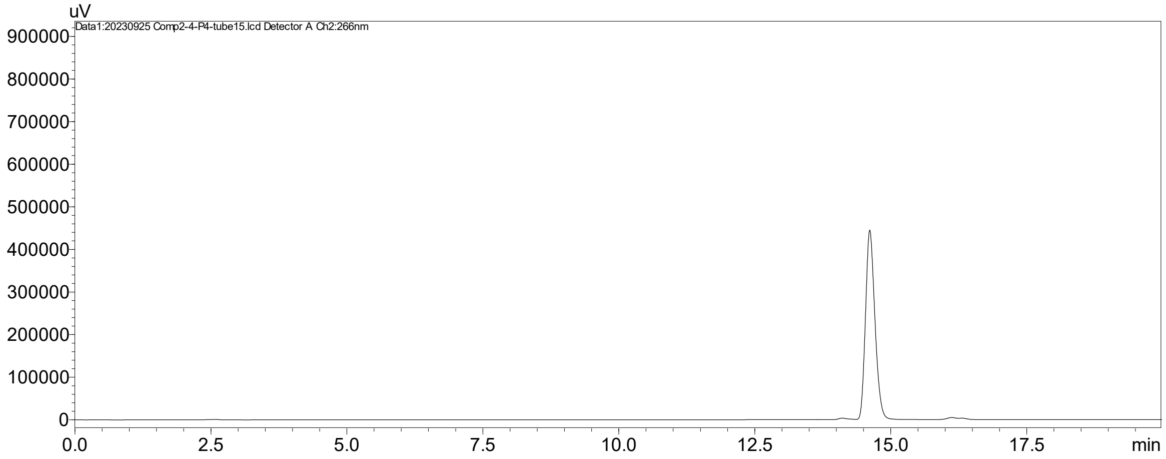

Compound 35

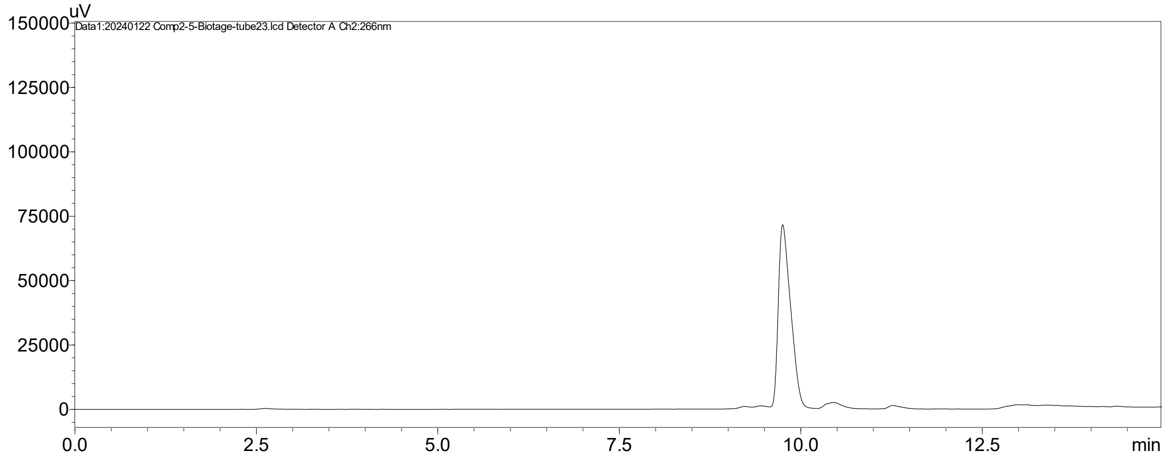

Compound 36

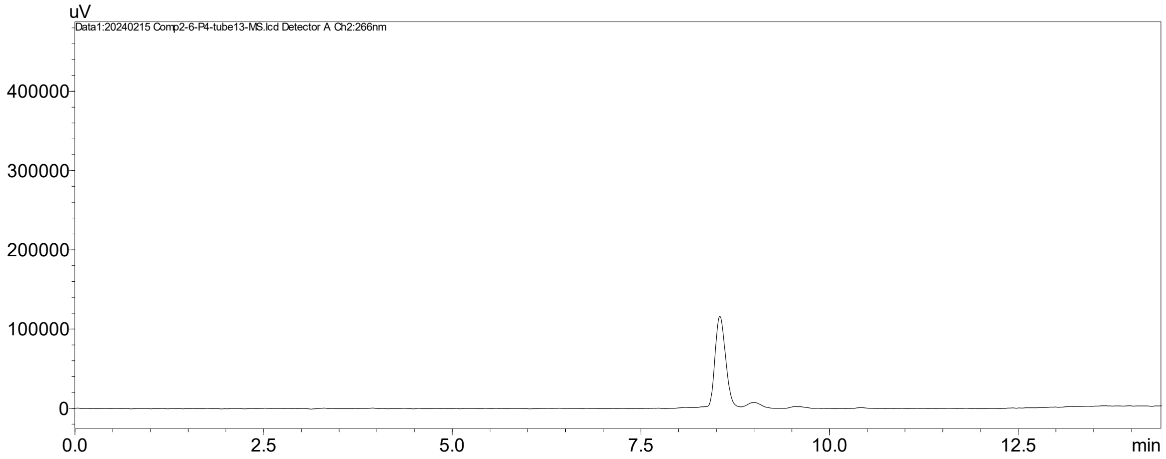

Compound 37

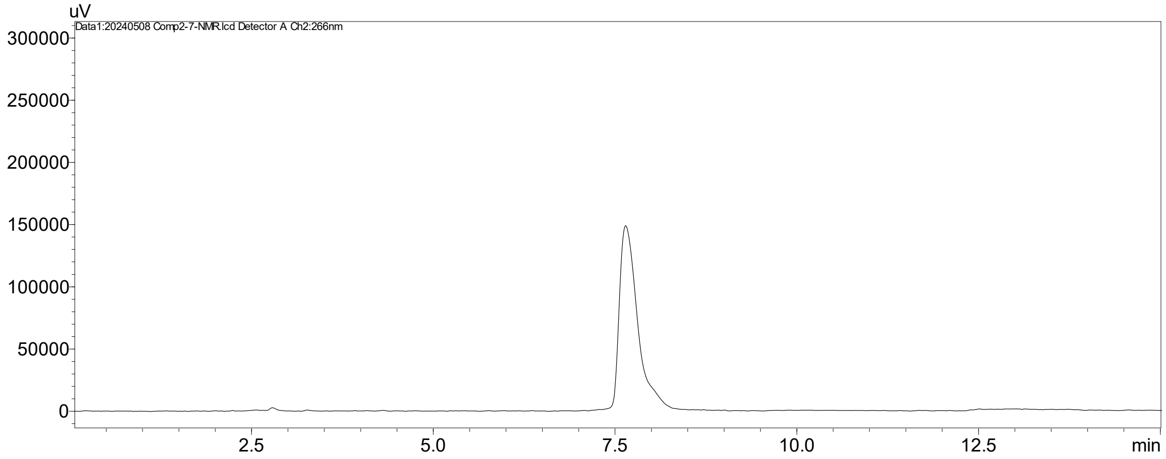

Compound 38

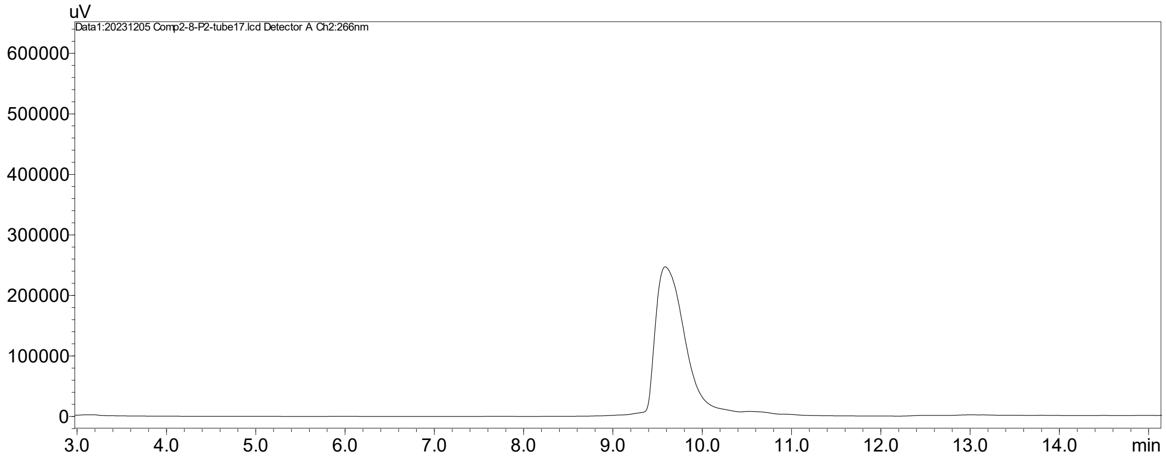

Compound 39

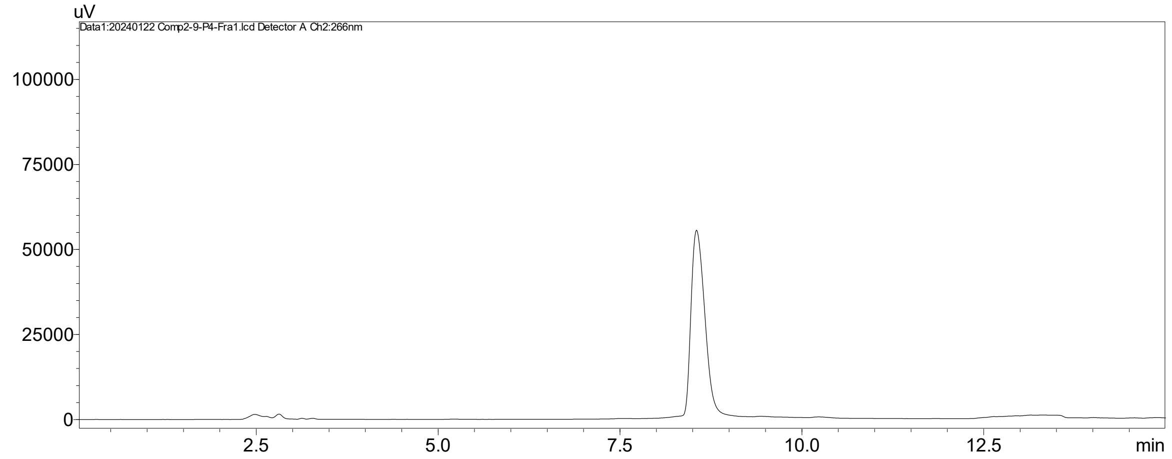

Compound 40

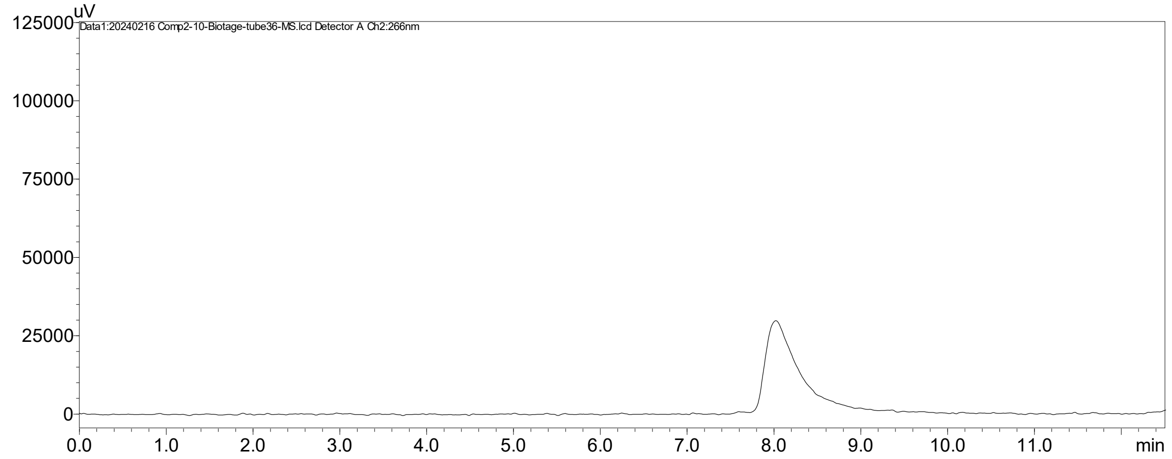

Compound 41

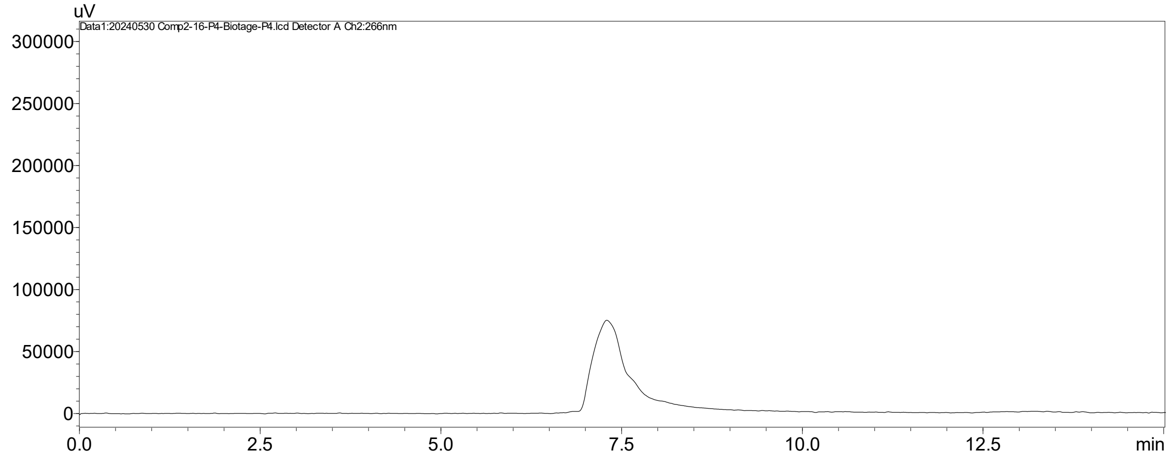

Compound 42

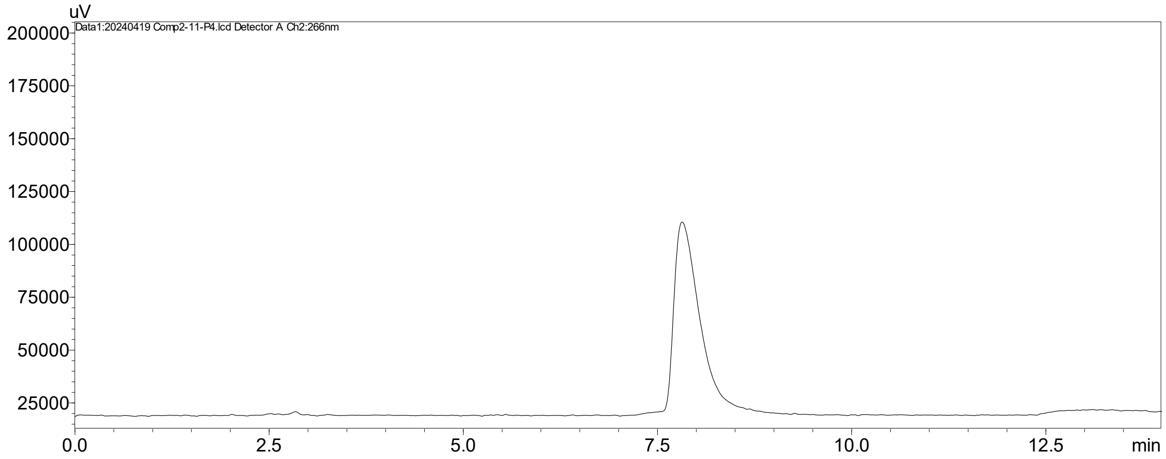

Compound 43

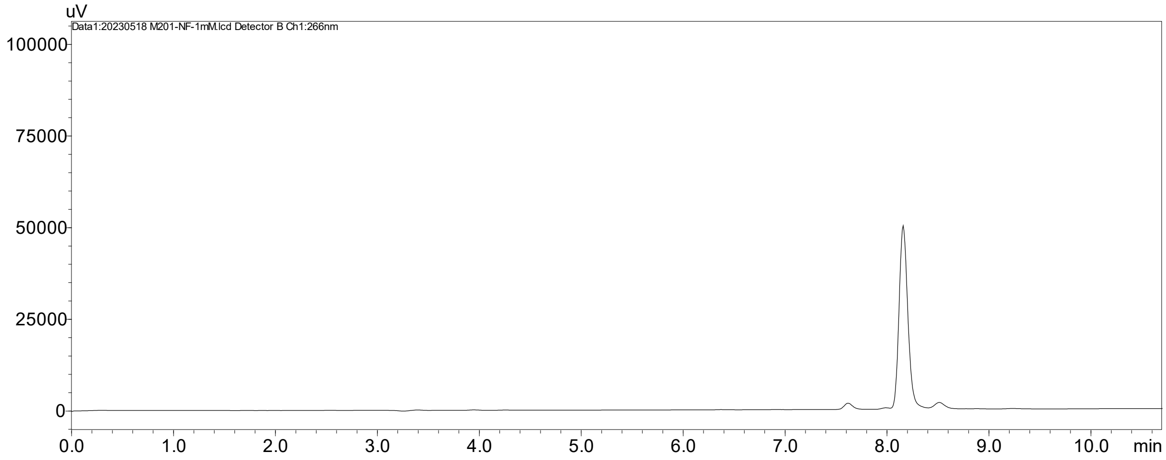

Compound 44

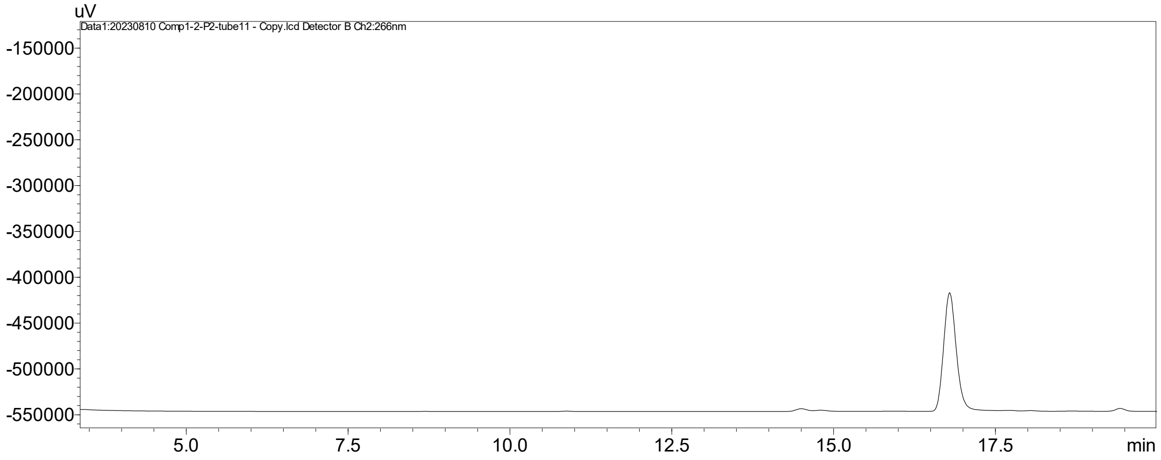

Compound 45

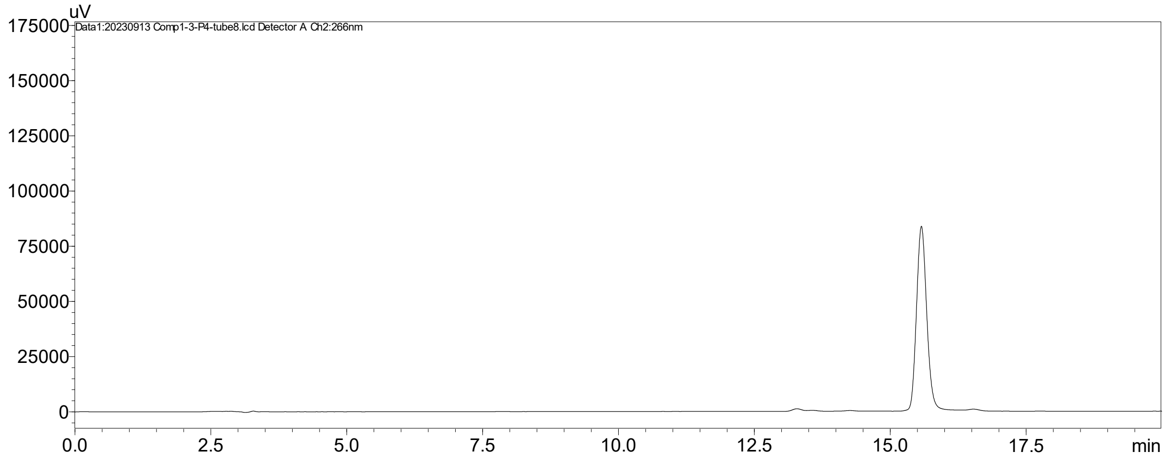

Compound 46

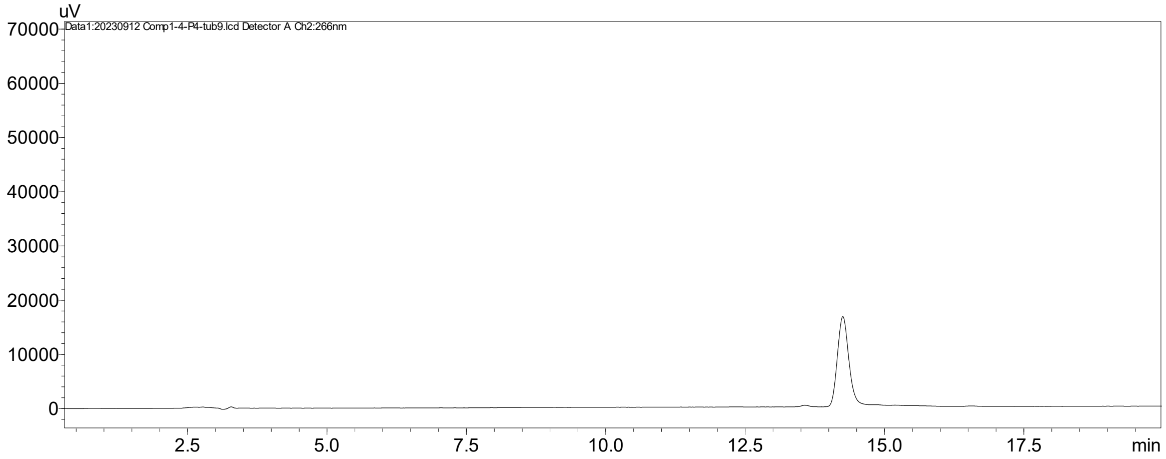

Compound 47

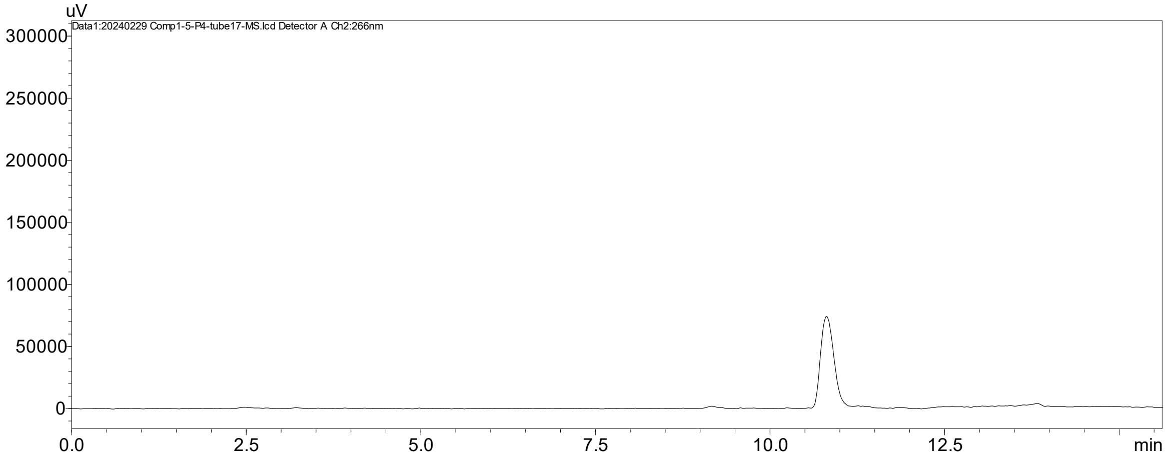

Compound 48

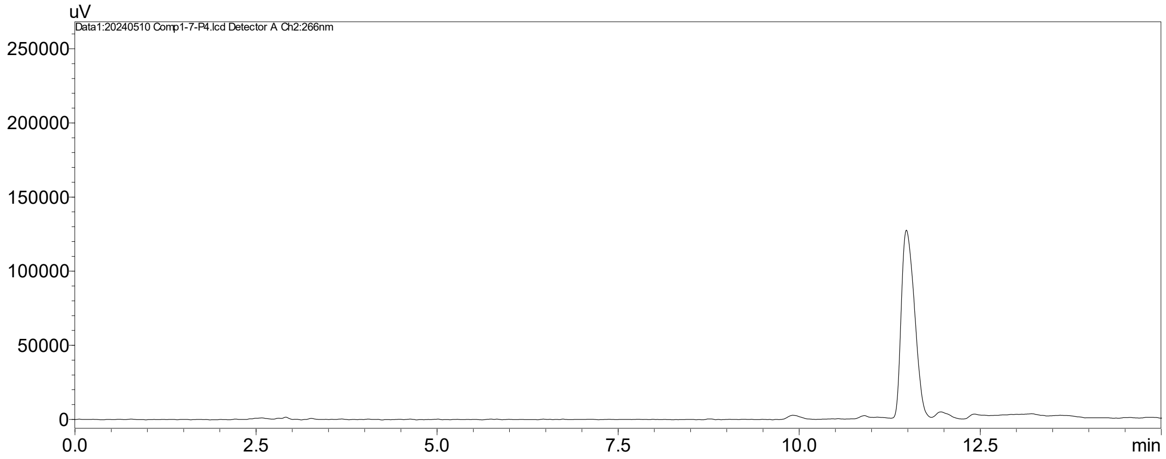

Compound 49

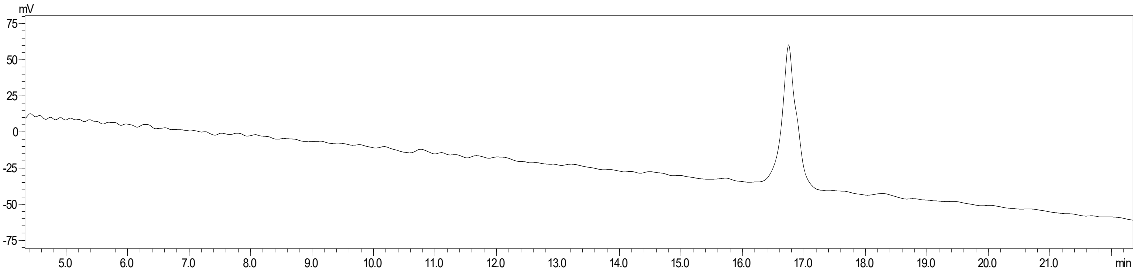

Compound 50

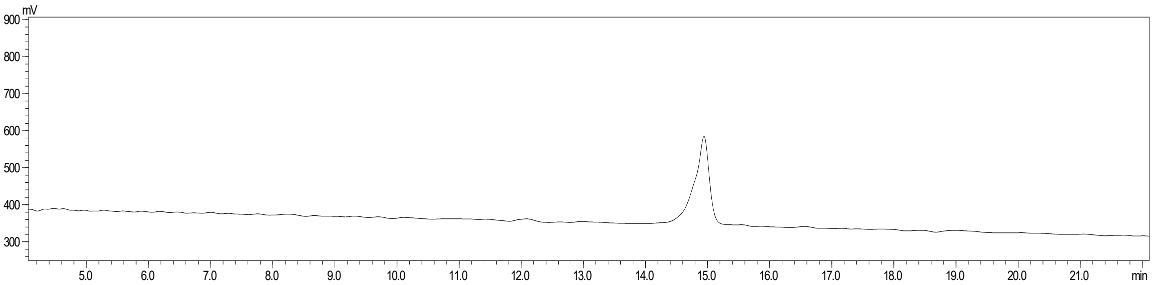

Compound 51

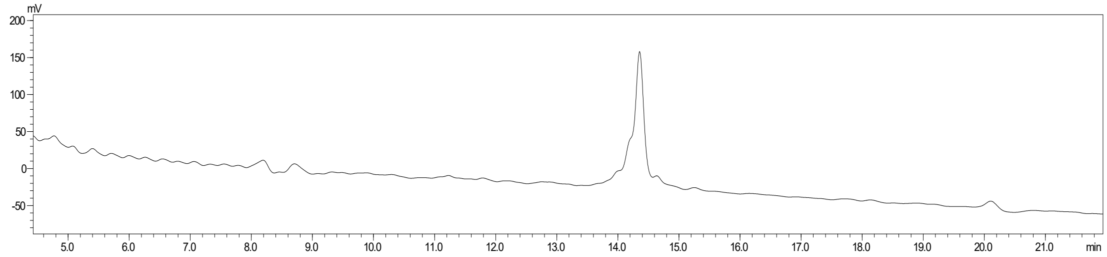

Compound 52

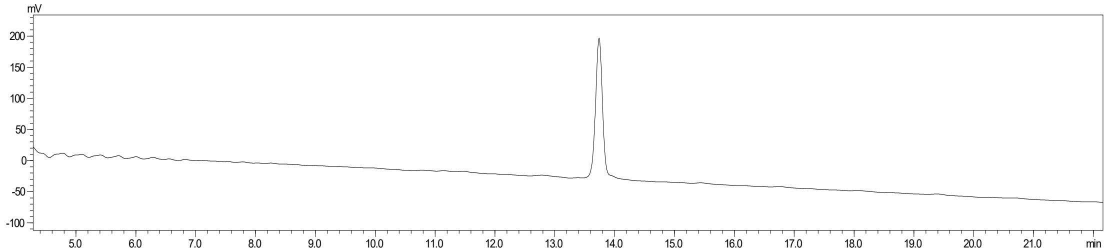

Compound 53

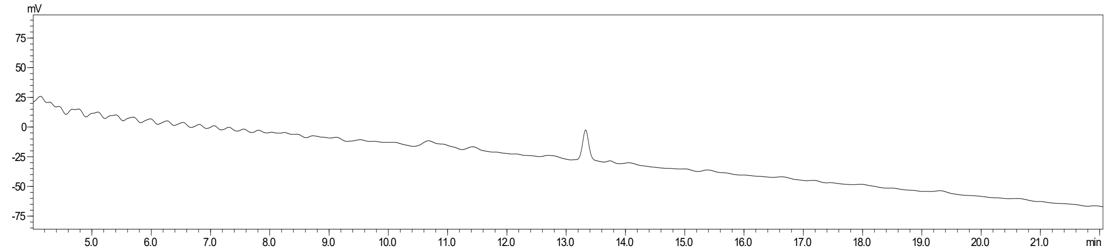

Compound 54

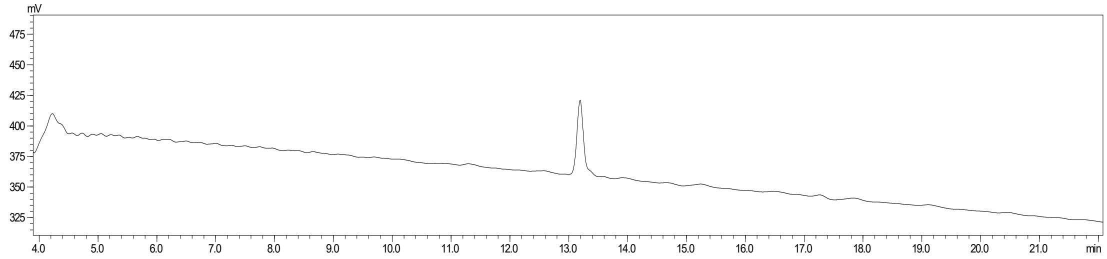

V. NMR Spectra

Compound 11

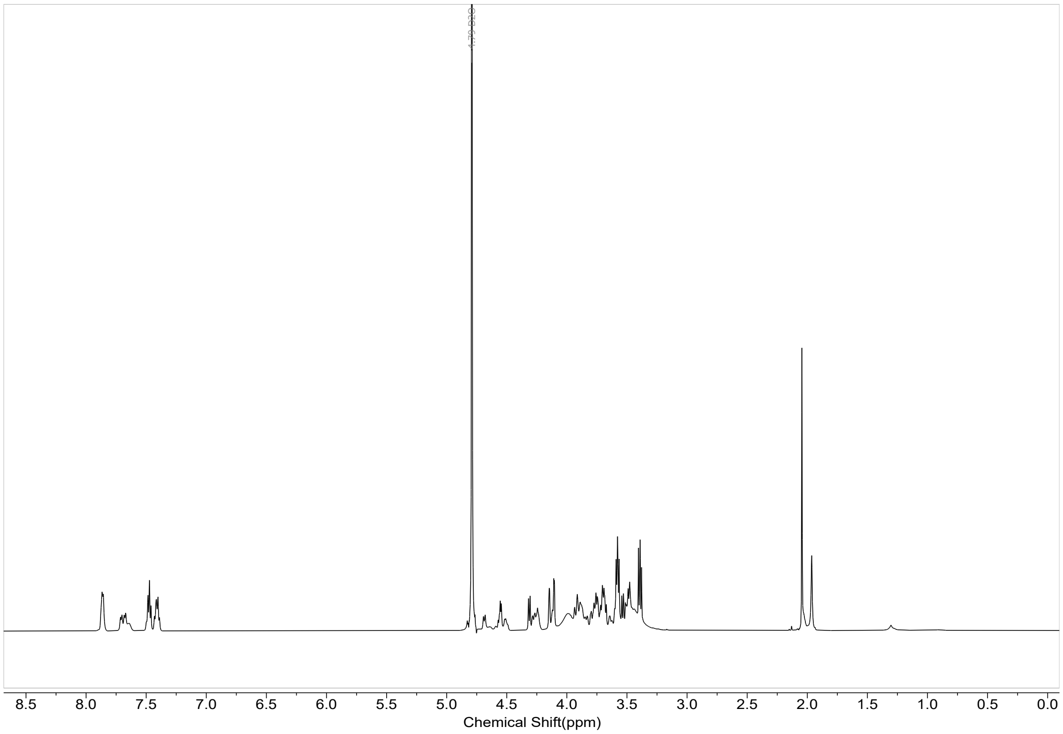

^{1}H NMR of Compound **11**

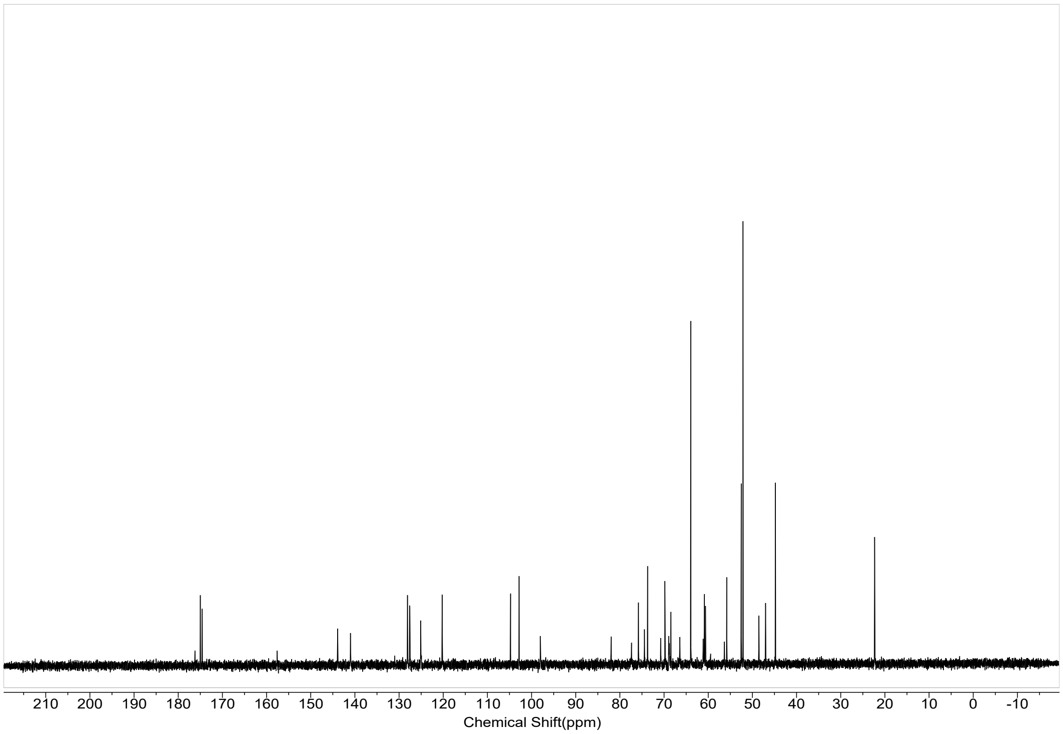

^{13}C NMR of Compound **11**

Compound 12

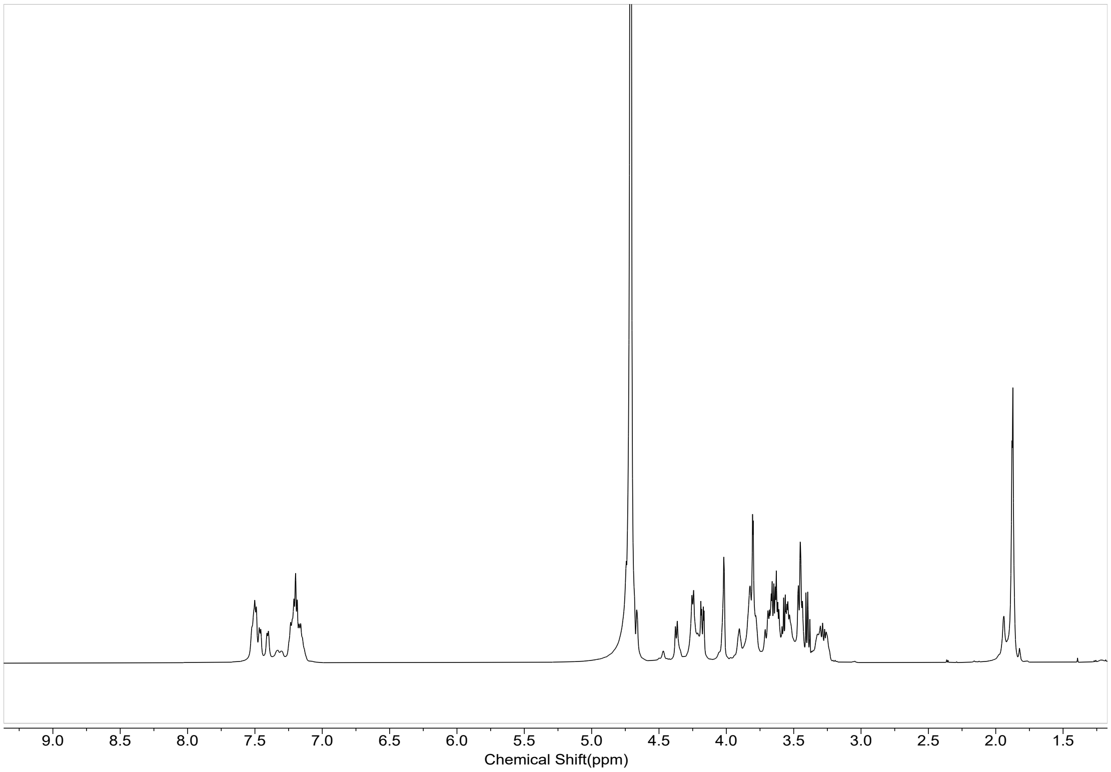

^{1}H NMR of Compound **12**

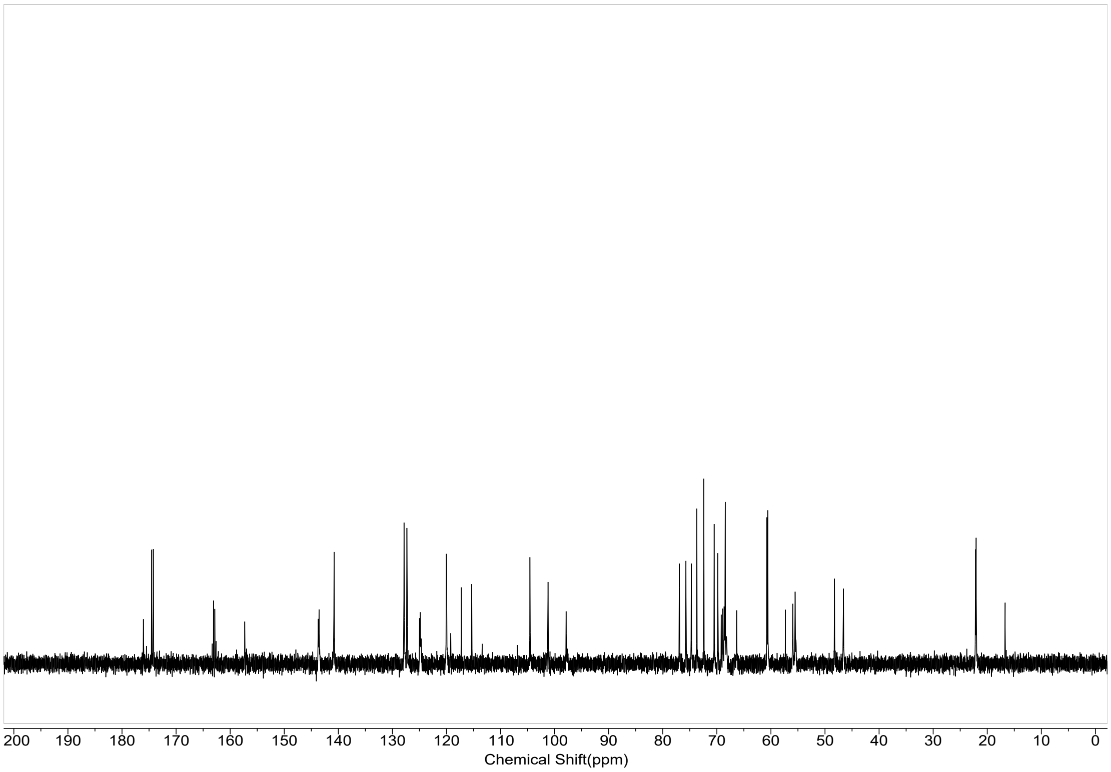

^{13}C NMR of Compound **12**

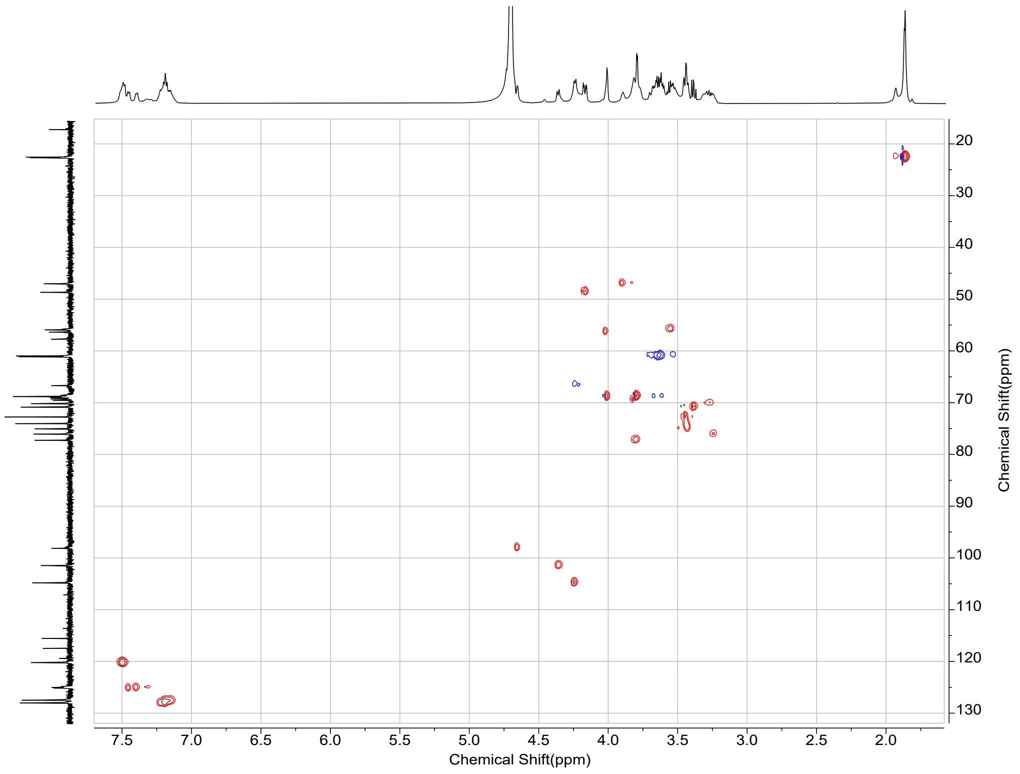

HSQC spectra of Compound **12**

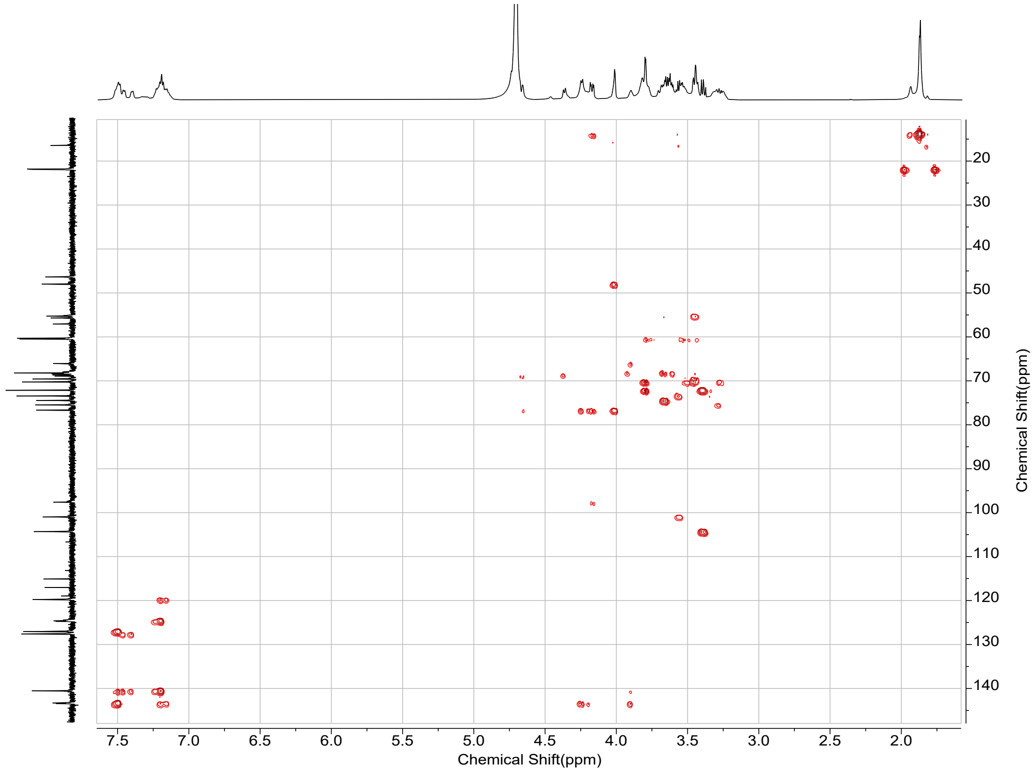

HMBC spectra of Compound **12**

Compound 21

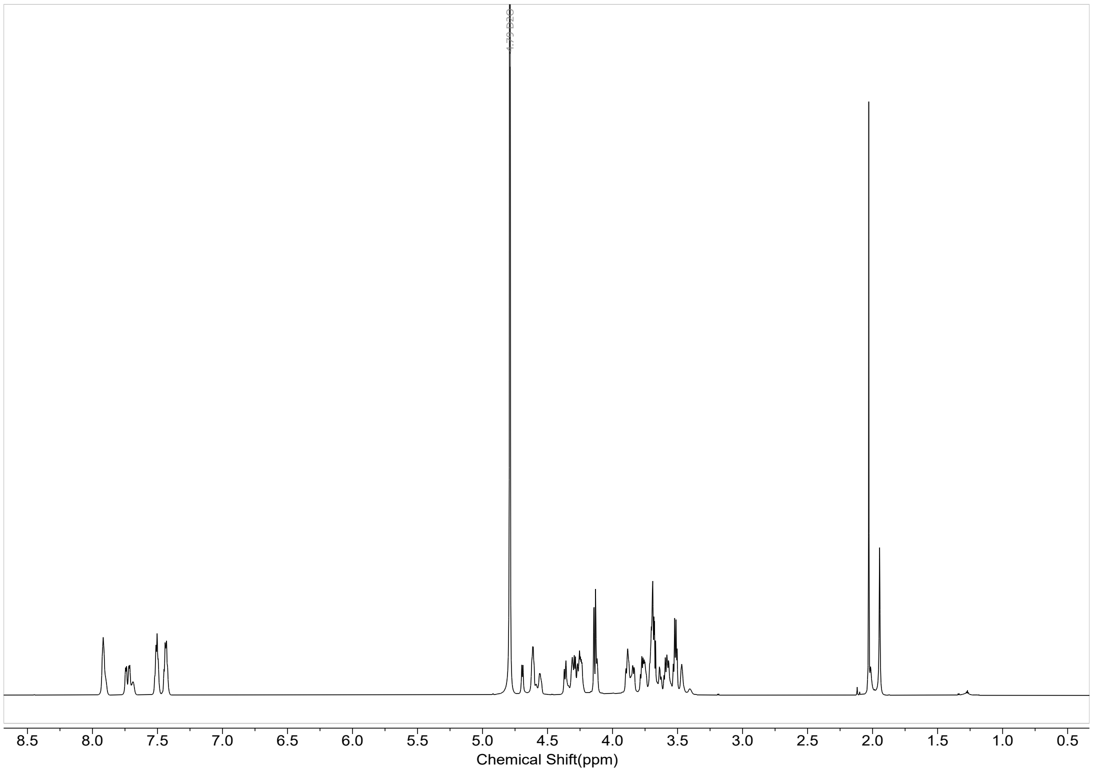

^{1}H NMR of Compound **21**

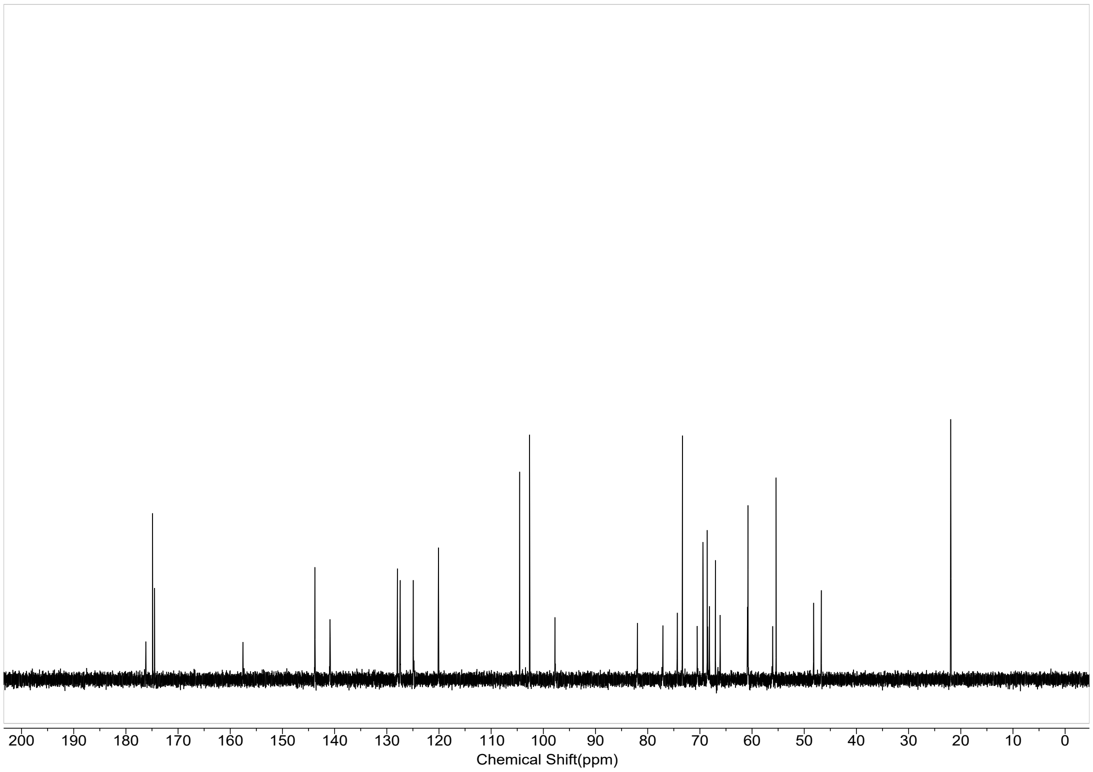

^{13}C NMR of Compound **21**

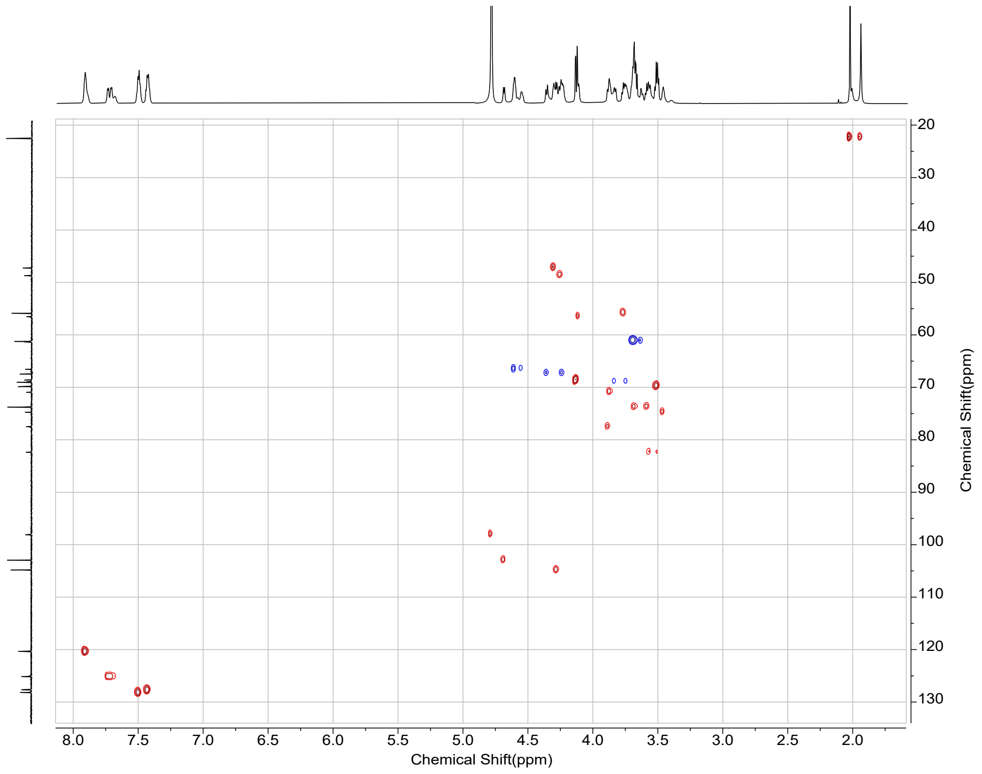

HSQC spectra of Compound **21**

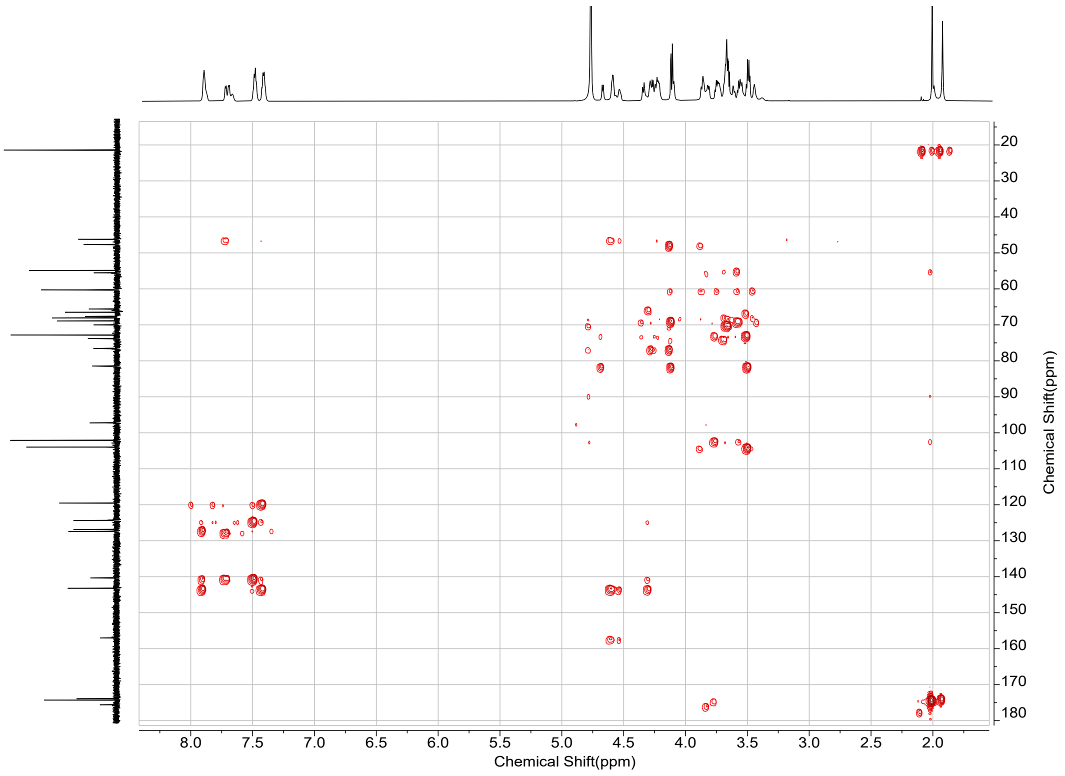

HMBC spectra of Compound **21**

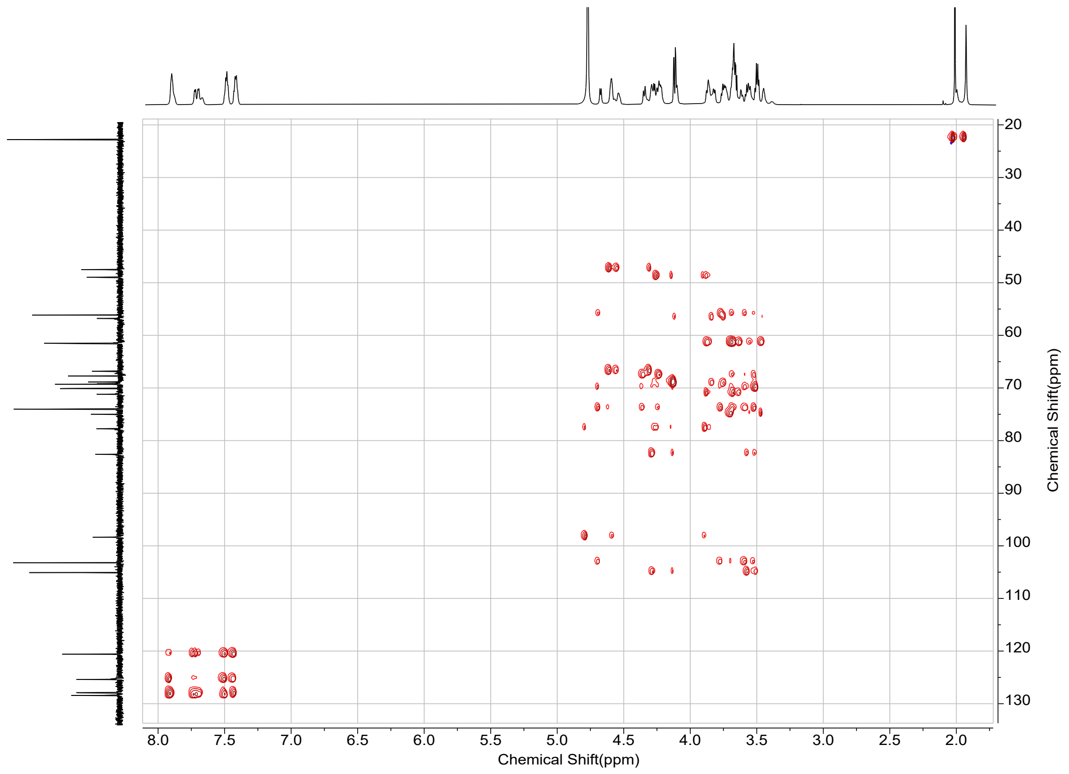

HSQC-TOCSY spectra of Compound **21**

Compound 22

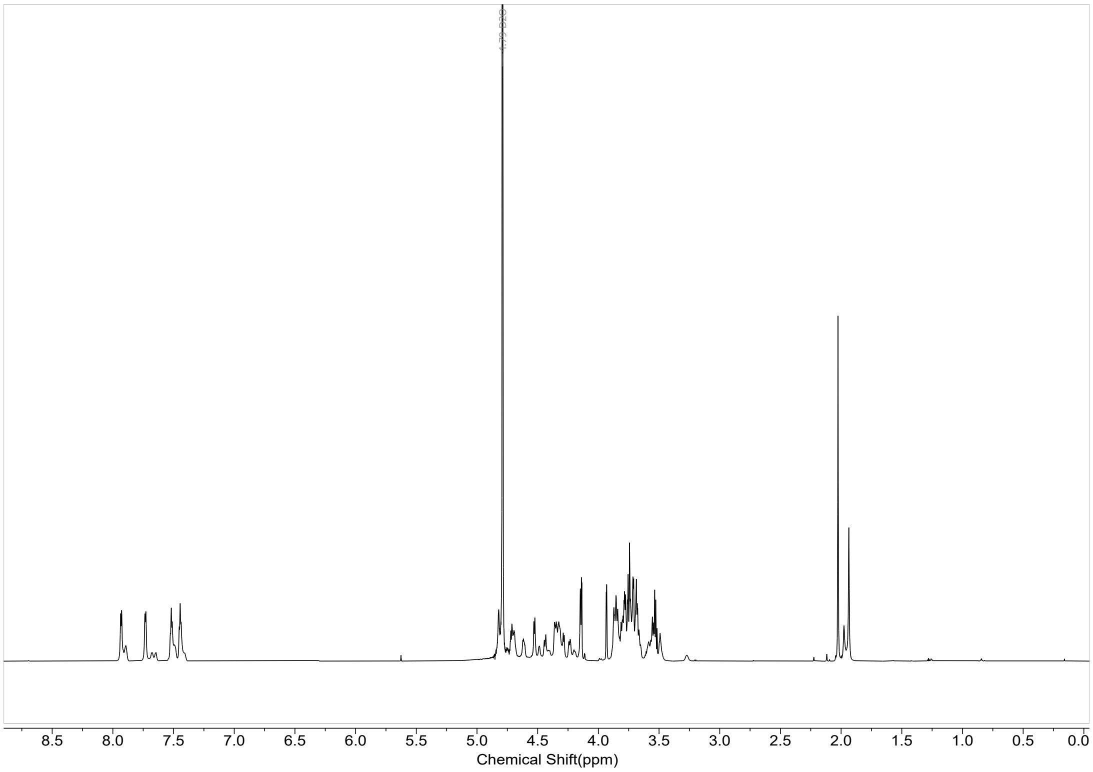

^{1}H NMR of Compound **22**

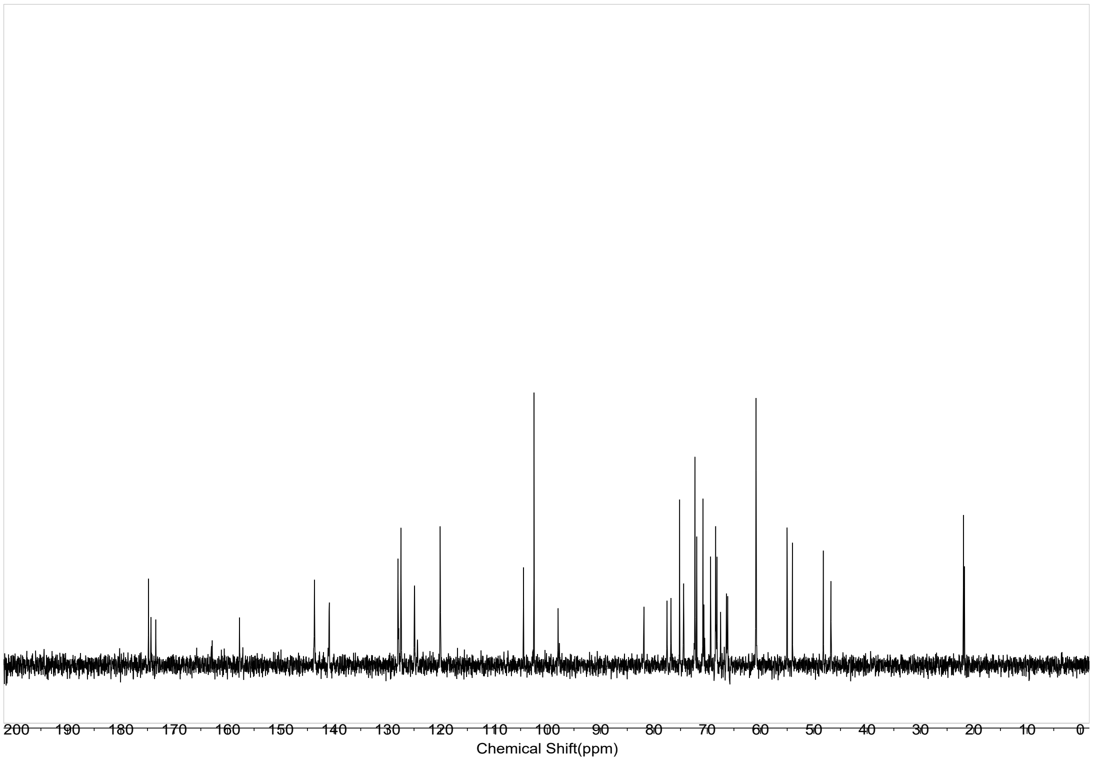

^{13}C NMR of Compound **22**

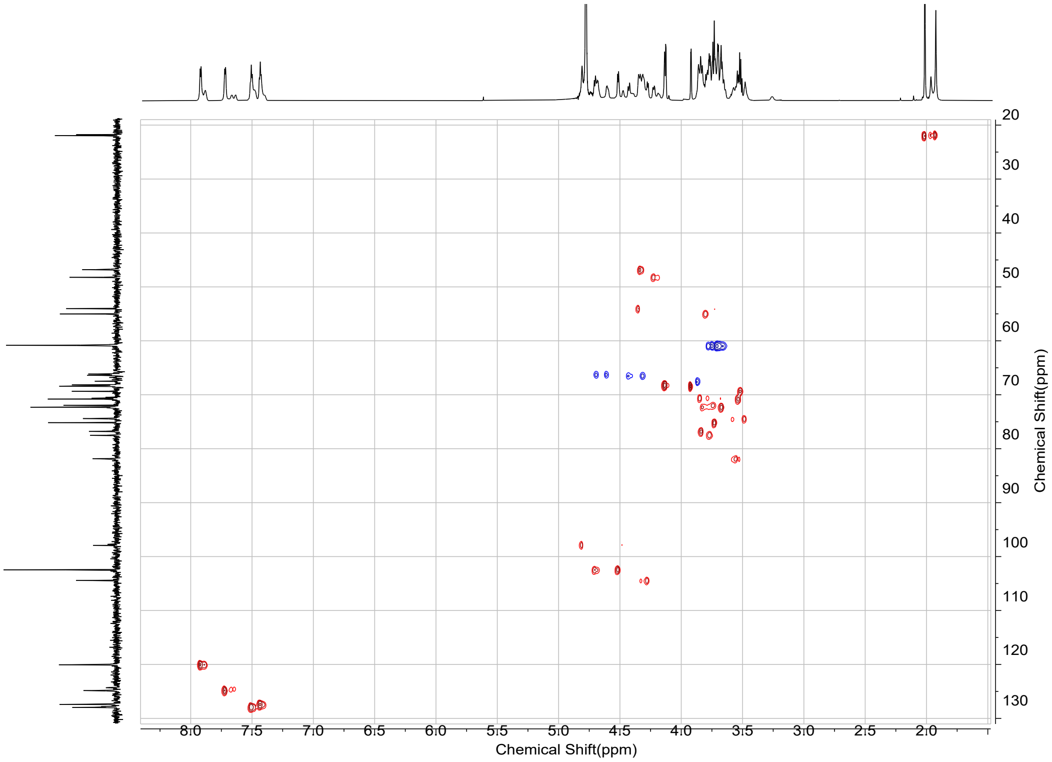

HSQC spectra of Compound **22**

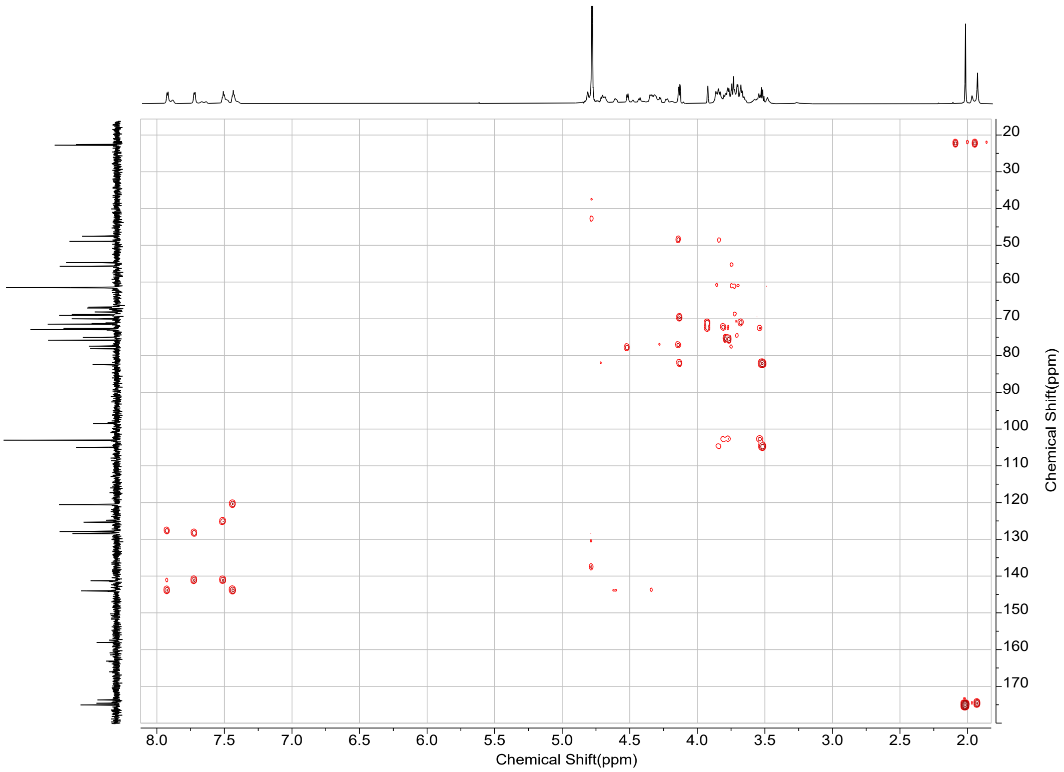

HMBC spectra of Compound **22**

Compound 23

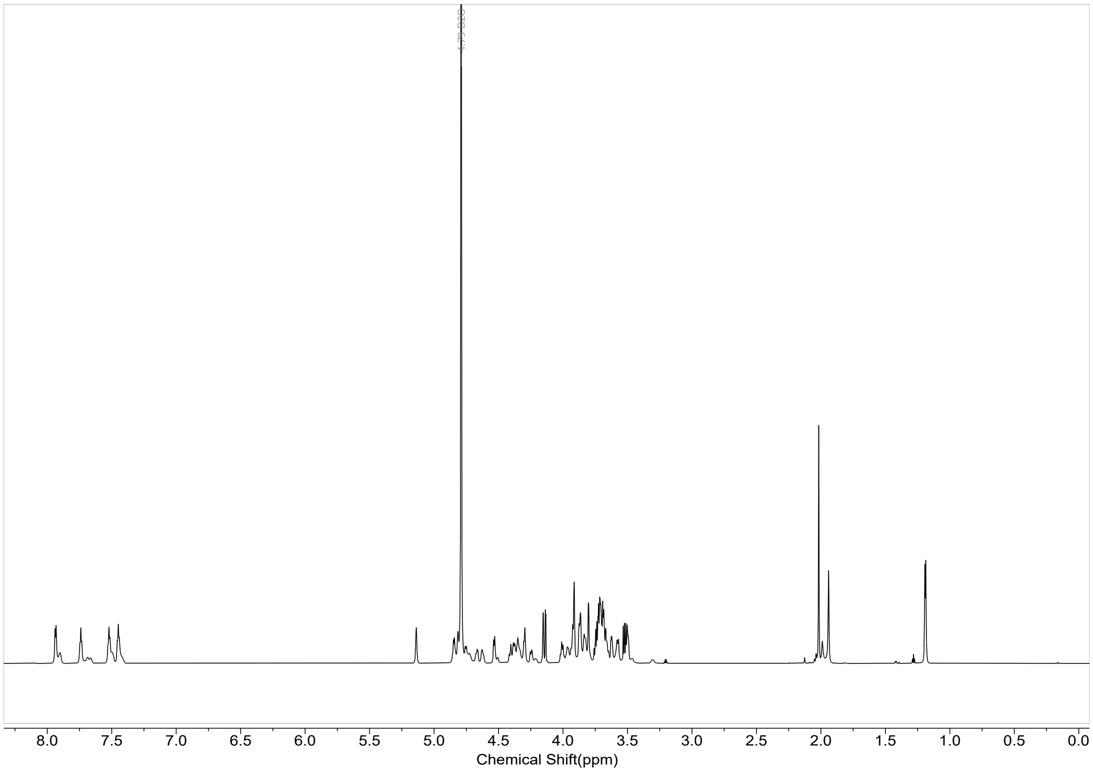

^{1}H NMR of Compound **23**

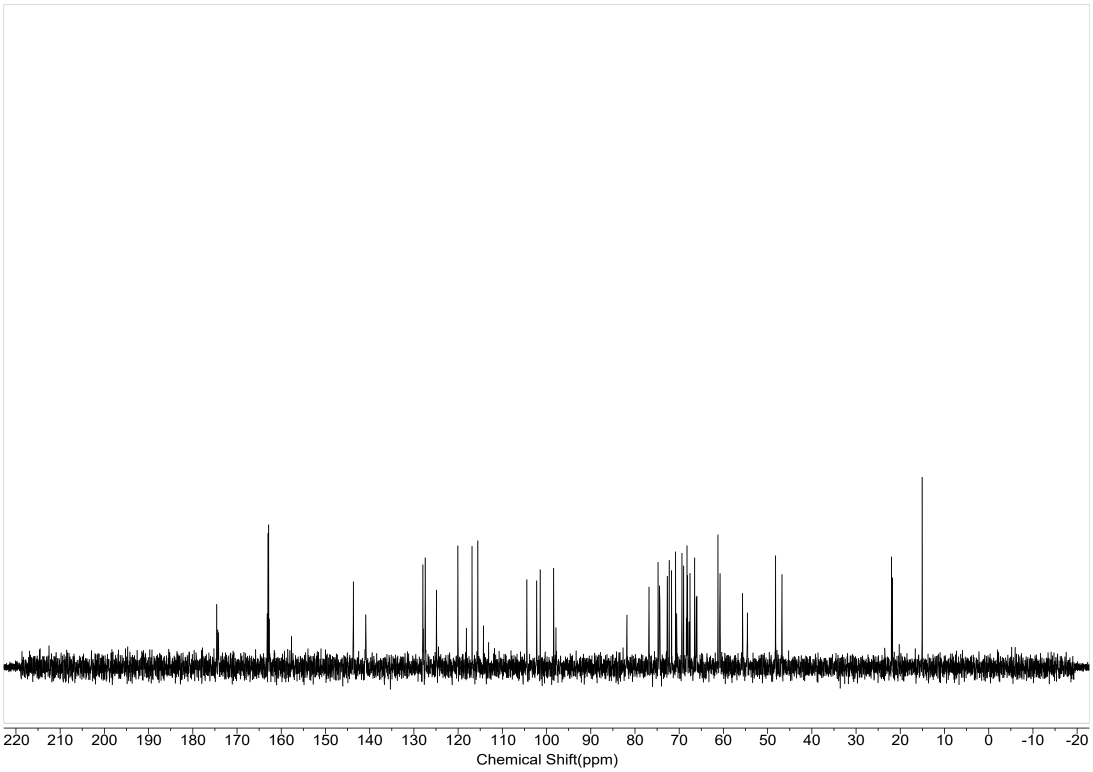

^{13}C NMR of Compound **23**

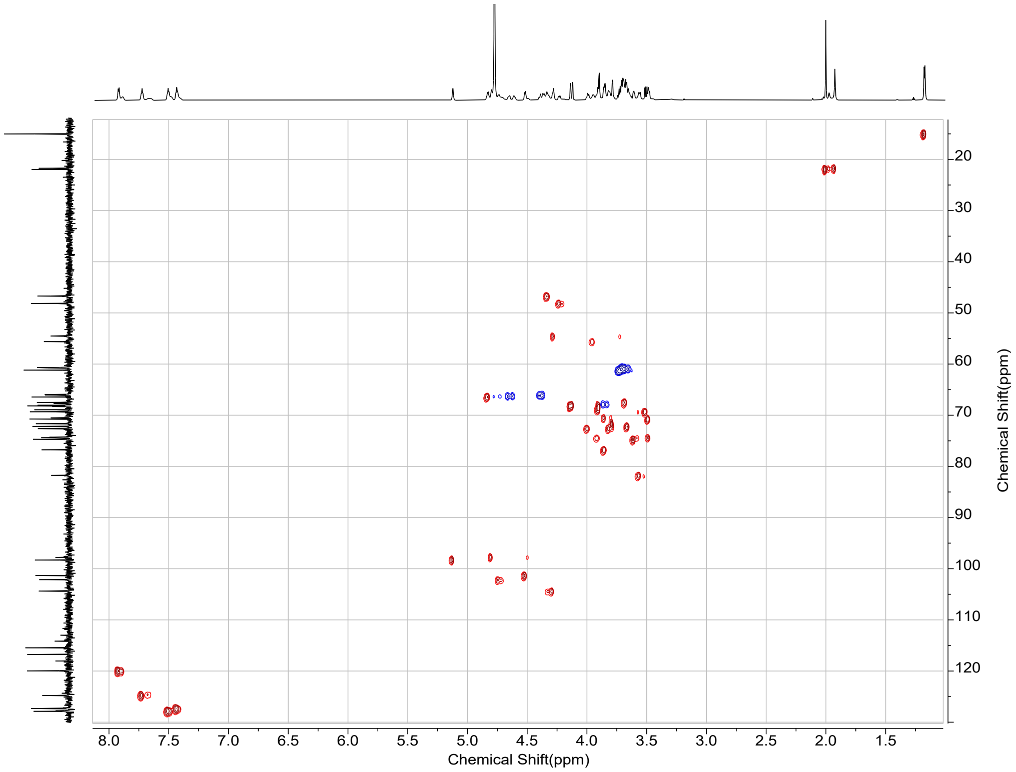

HSQC spectra of Compound **23**

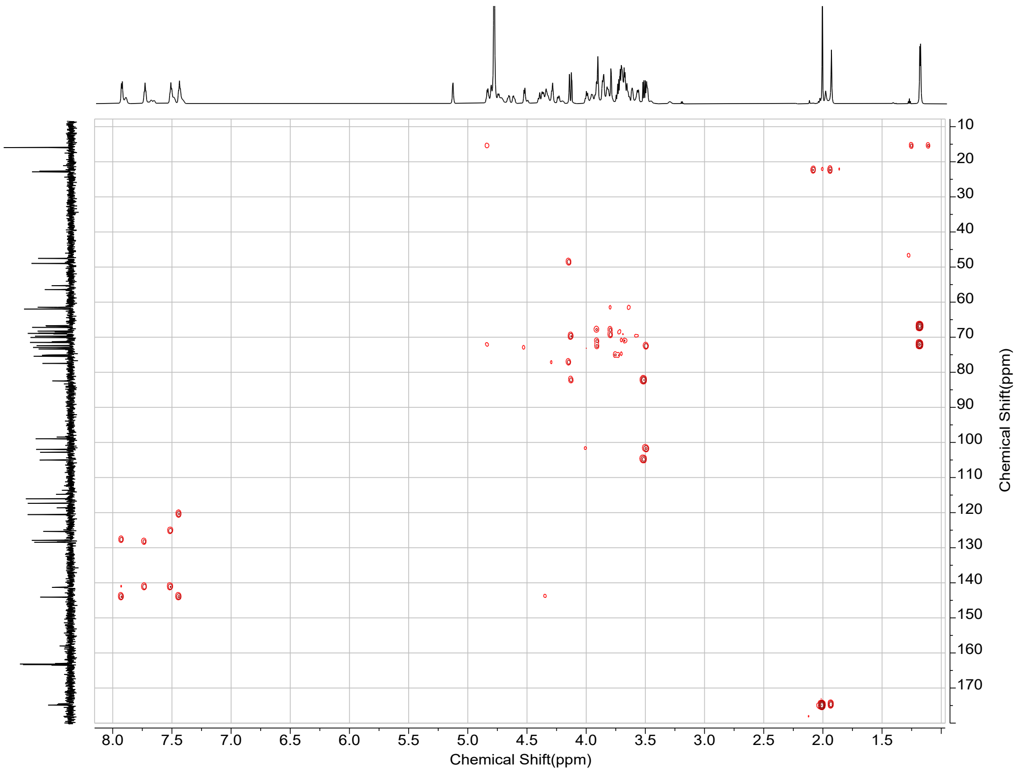

HMBC spectra of Compound **23**

Compound 24

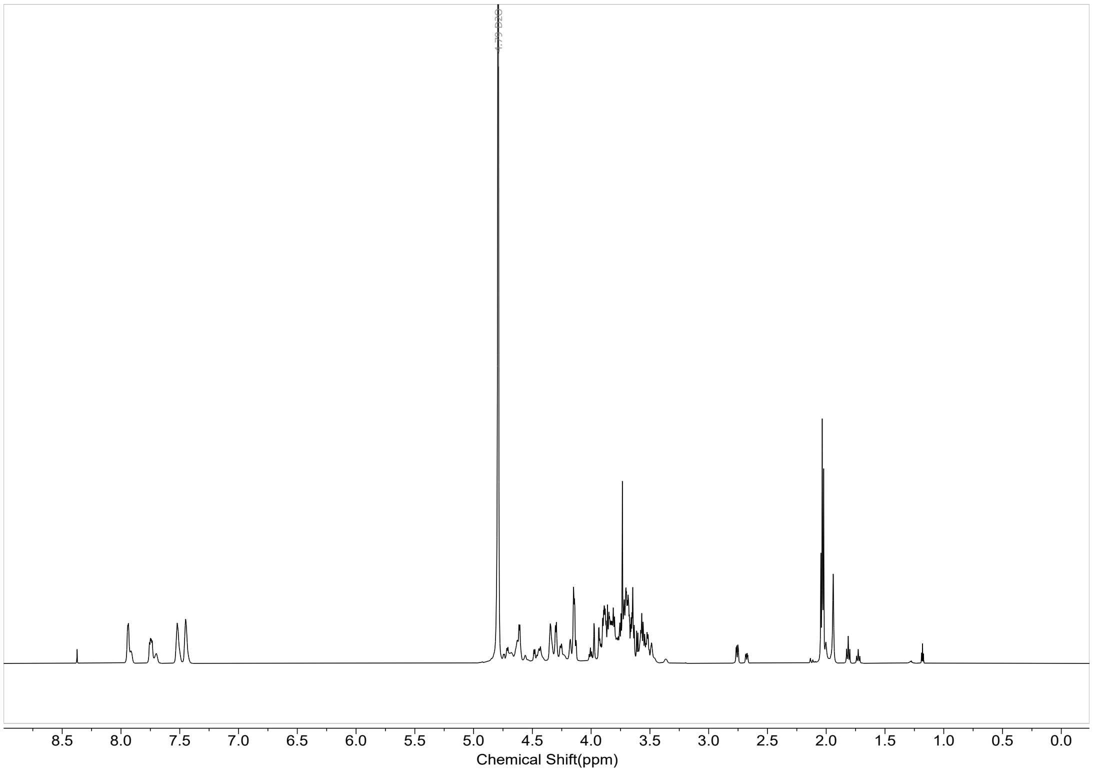

^{1}H NMR of Compound **24**

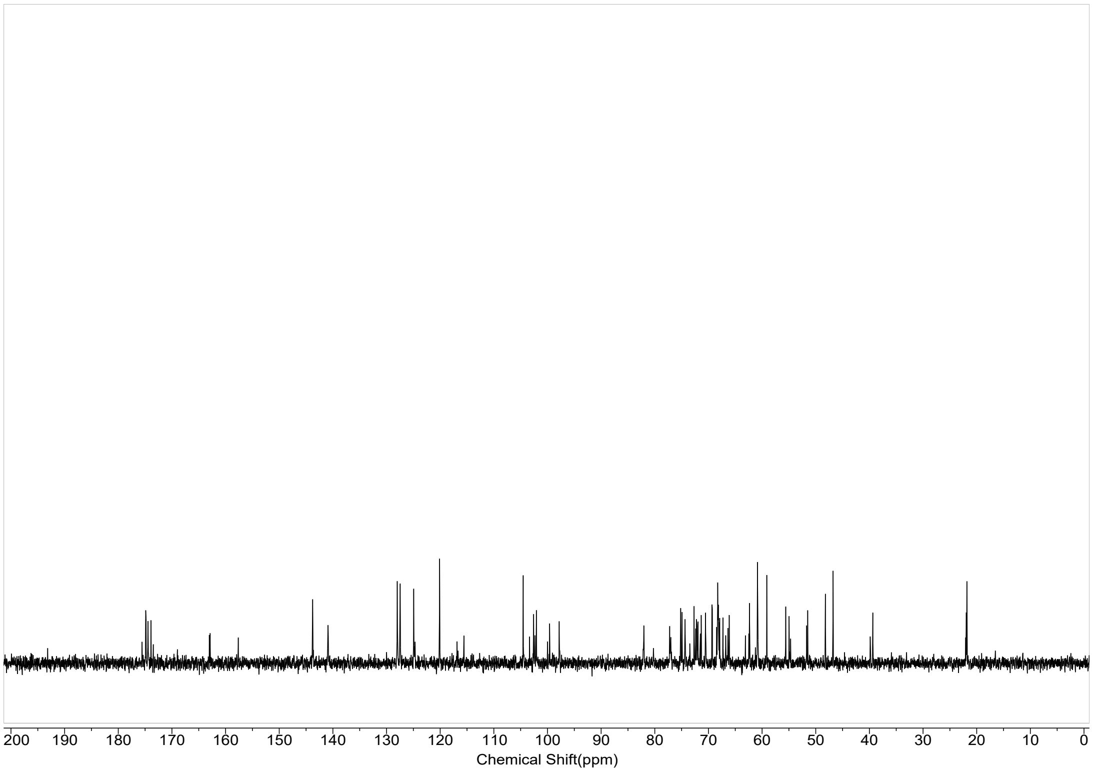

^{13}C NMR of Compound **24**

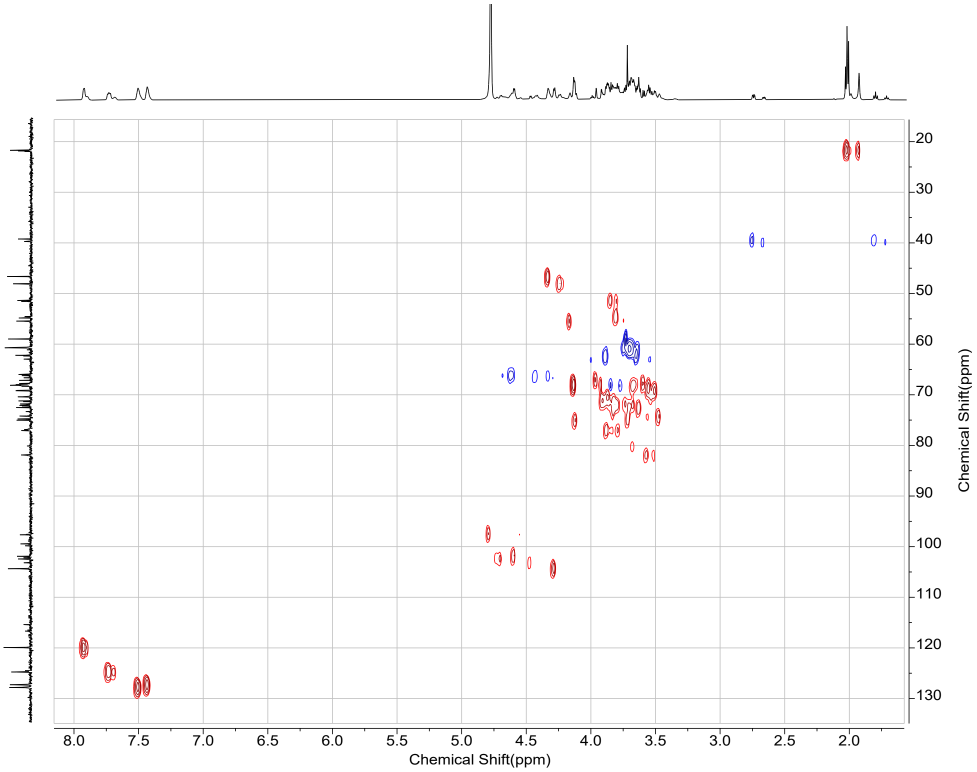

HSQC spectra of Compound **24**

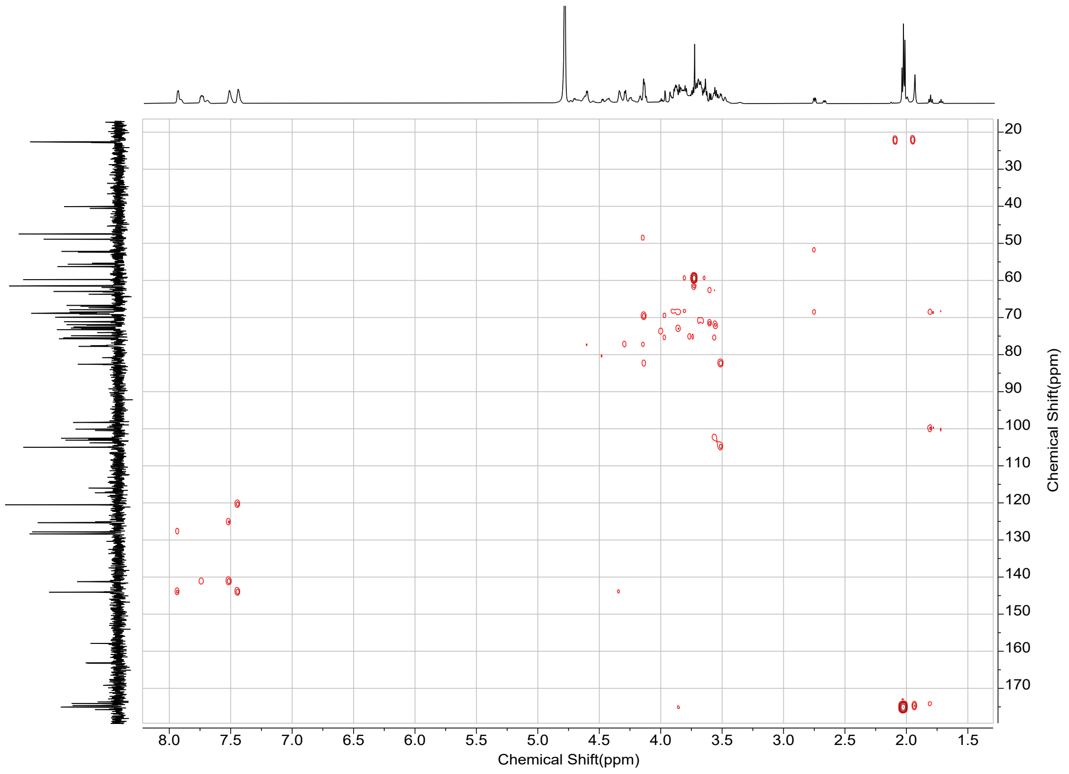

HMBC spectra of Compound **24**

Compound 25

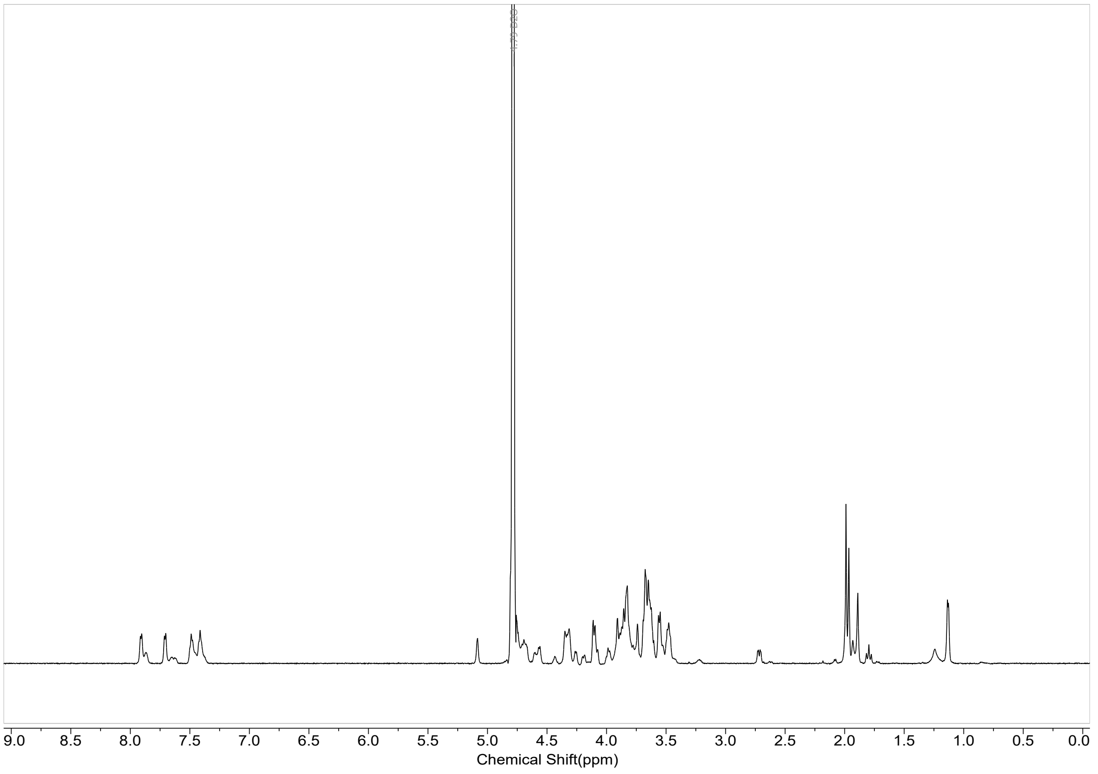

^{1}H NMR of Compound **25**

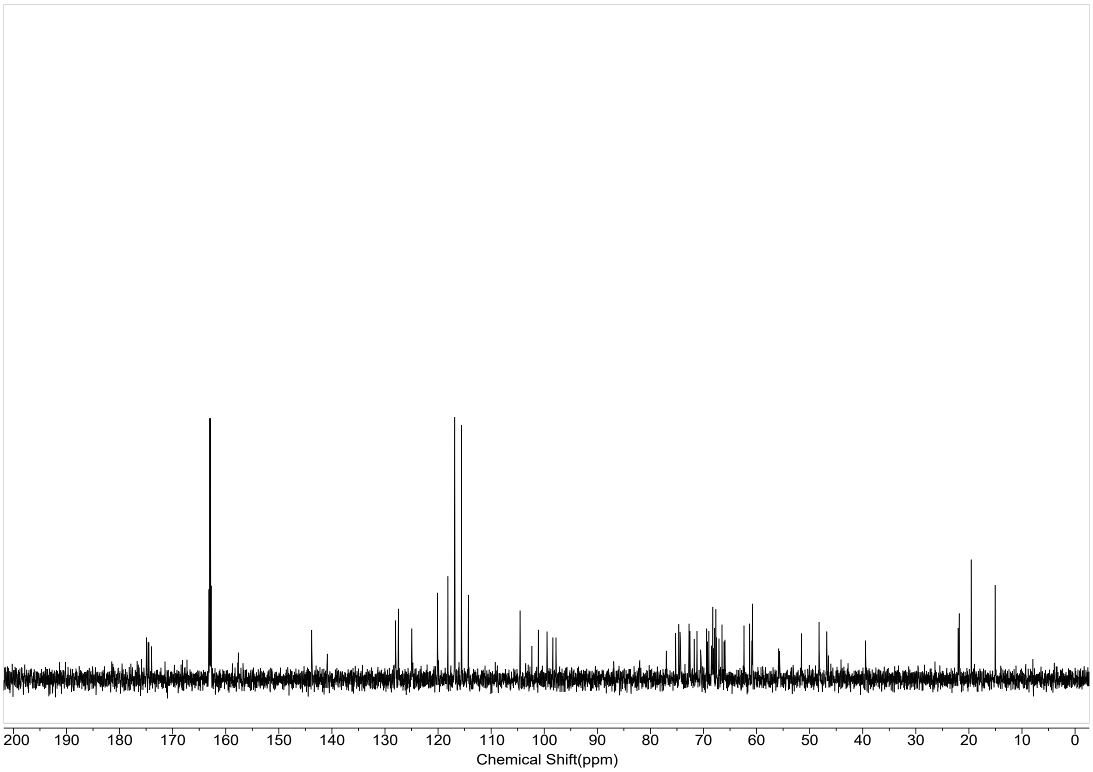

^{13}C NMR of Compound **25**

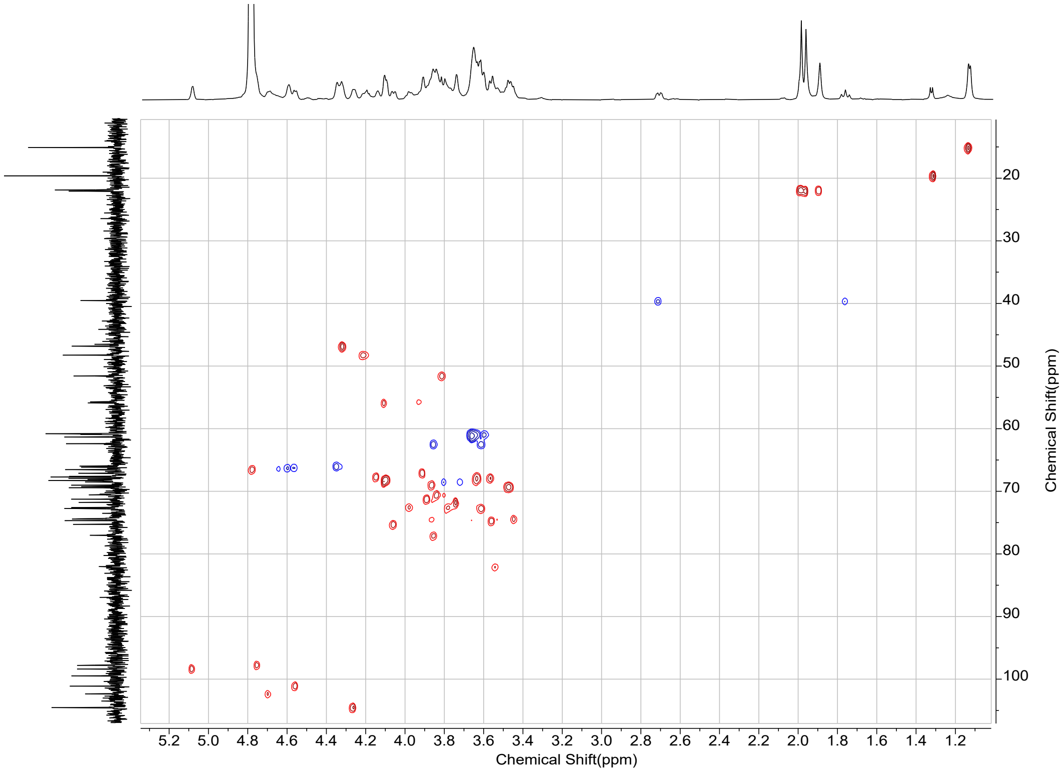

HSQC spectra of Compound **25**

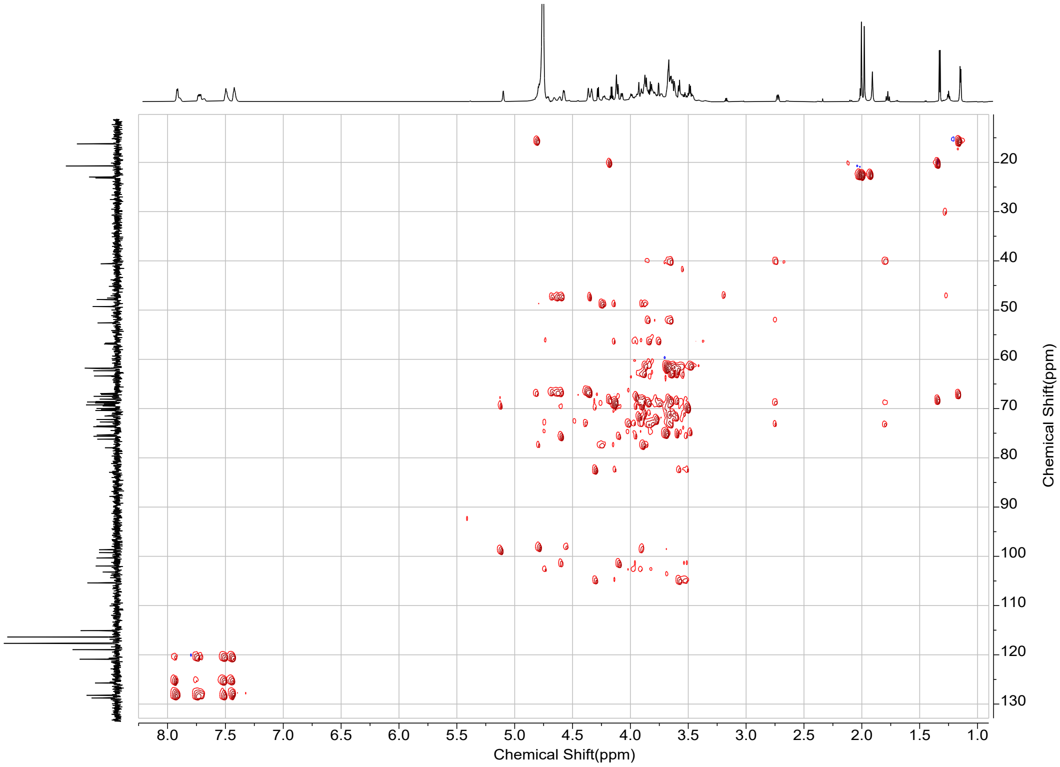

HSQC-TOCSY spectra of Compound **25**

Compound 26

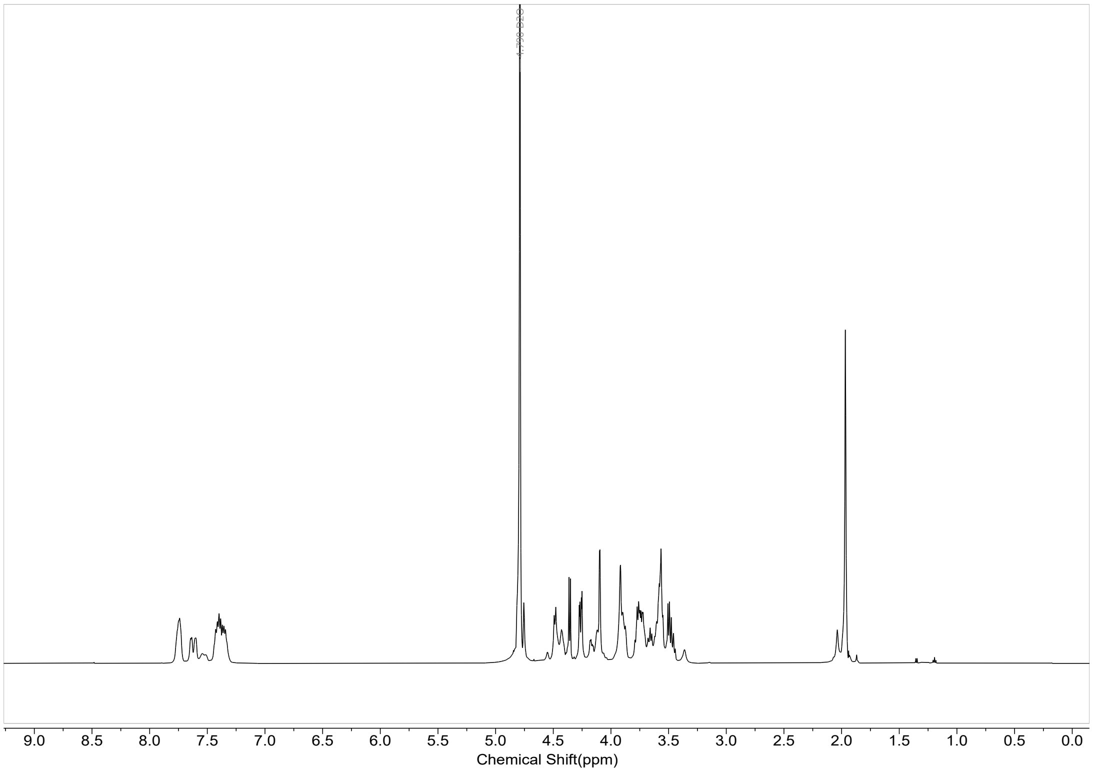

^{1}H NMR of Compound **26**

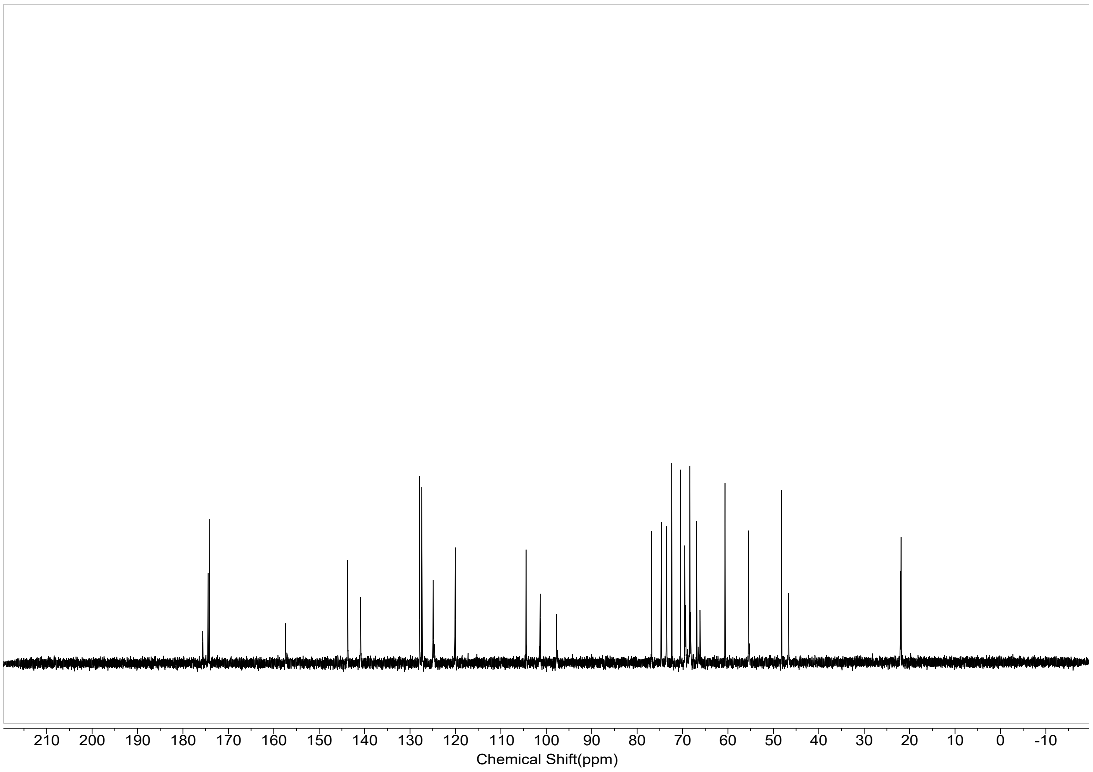

^{13}C NMR of Compound **26**

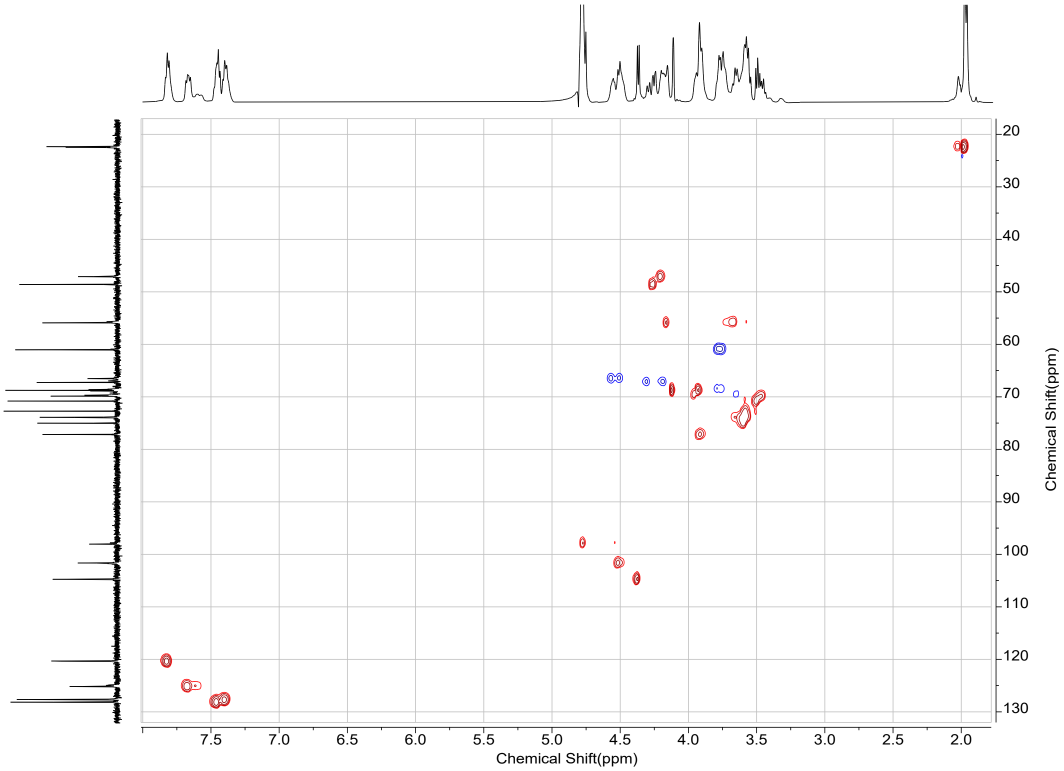

HSQC spectra of Compound **26**

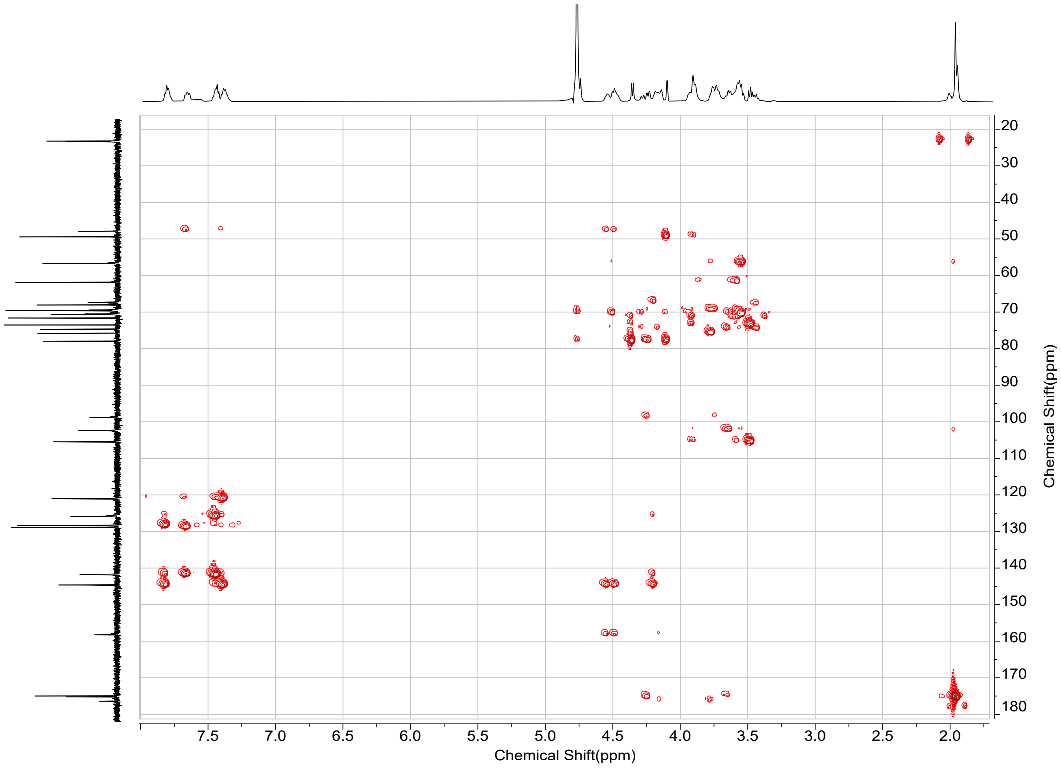

HMBC spectra of Compound **26**

Compound 27

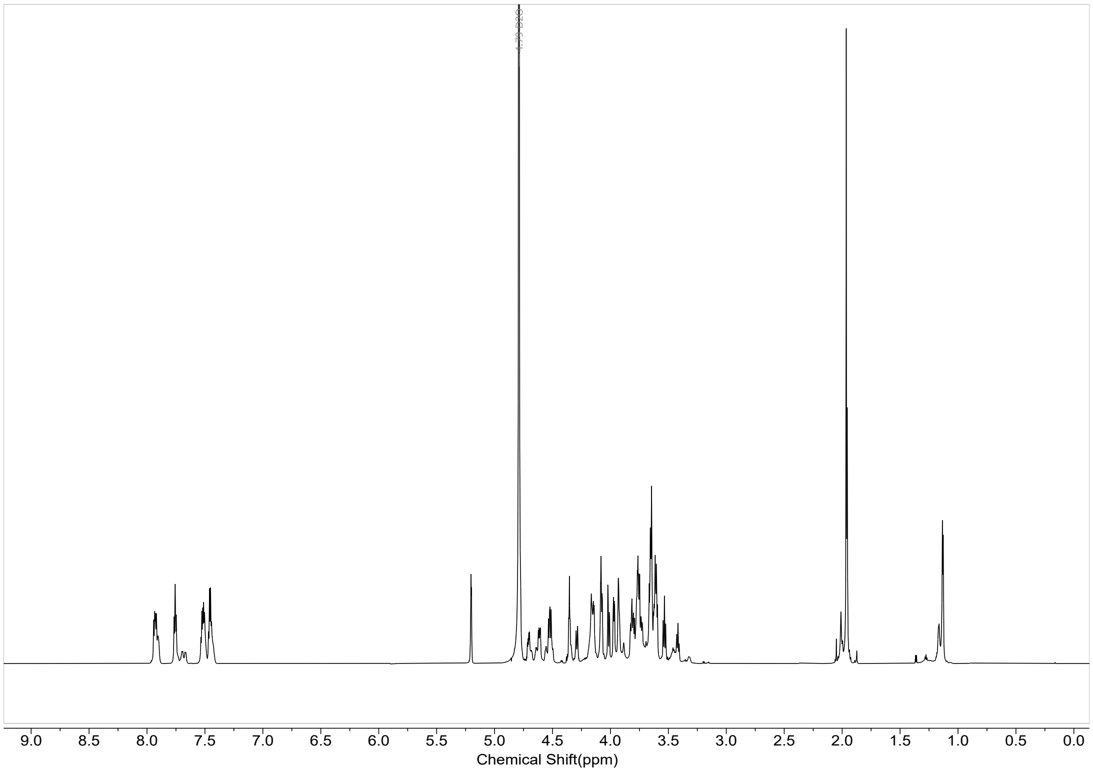

^{1}H NMR of Compound **27**

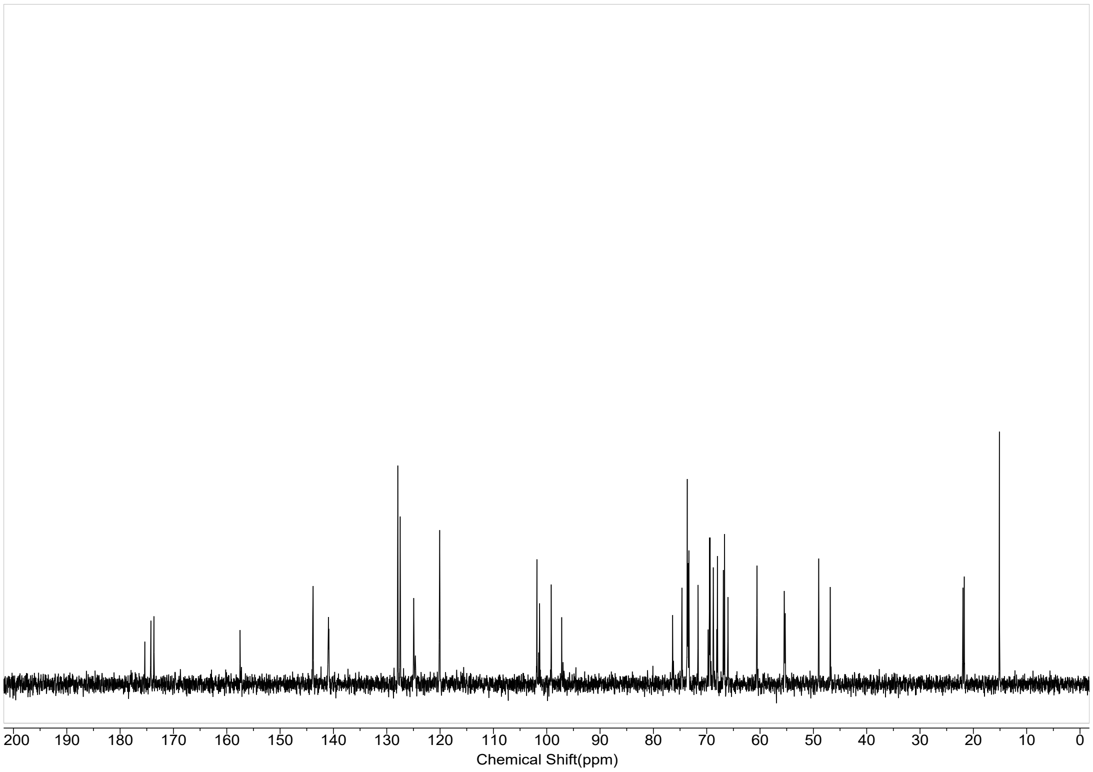

^{13}C NMR of Compound **27**

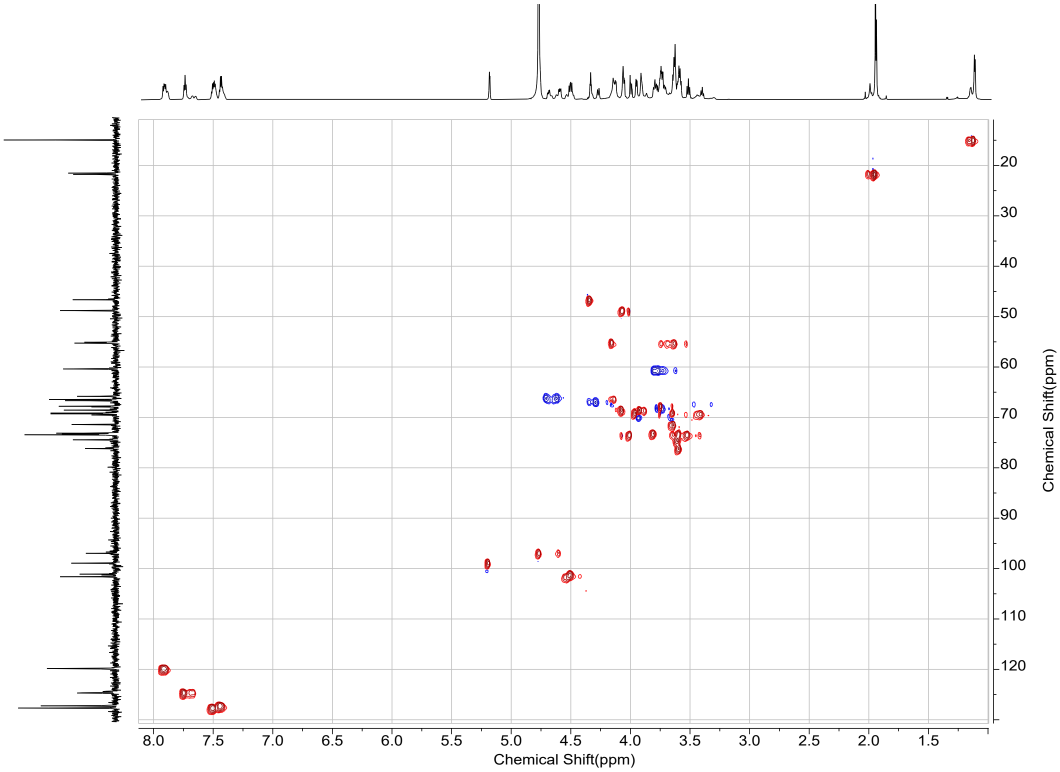

HSQC spectra of Compound **27**

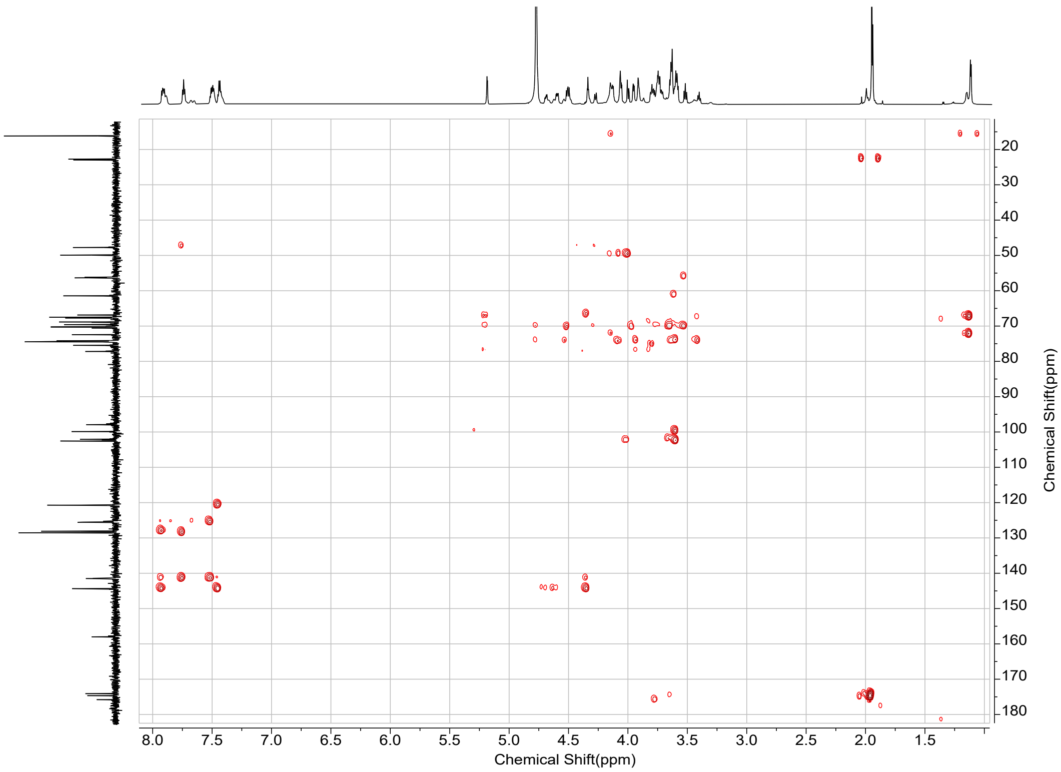

HMBC spectra of Compound **27**

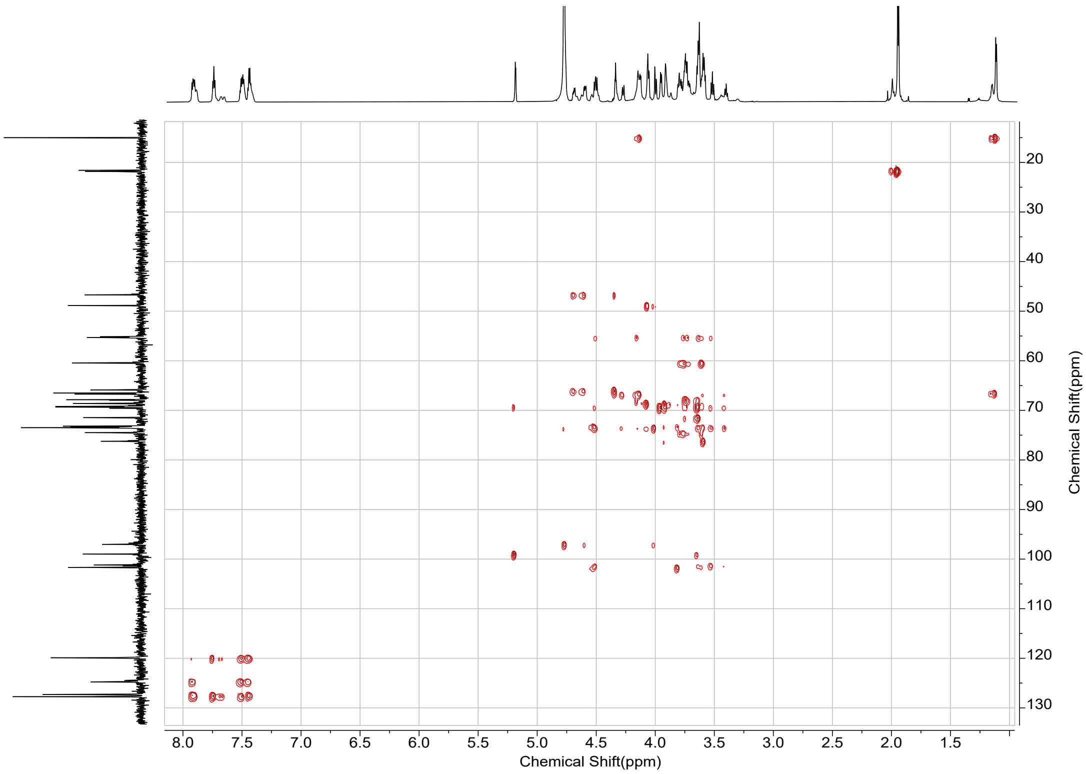

HSQC-TOCSY spectra of Compound **27**

Compound 28

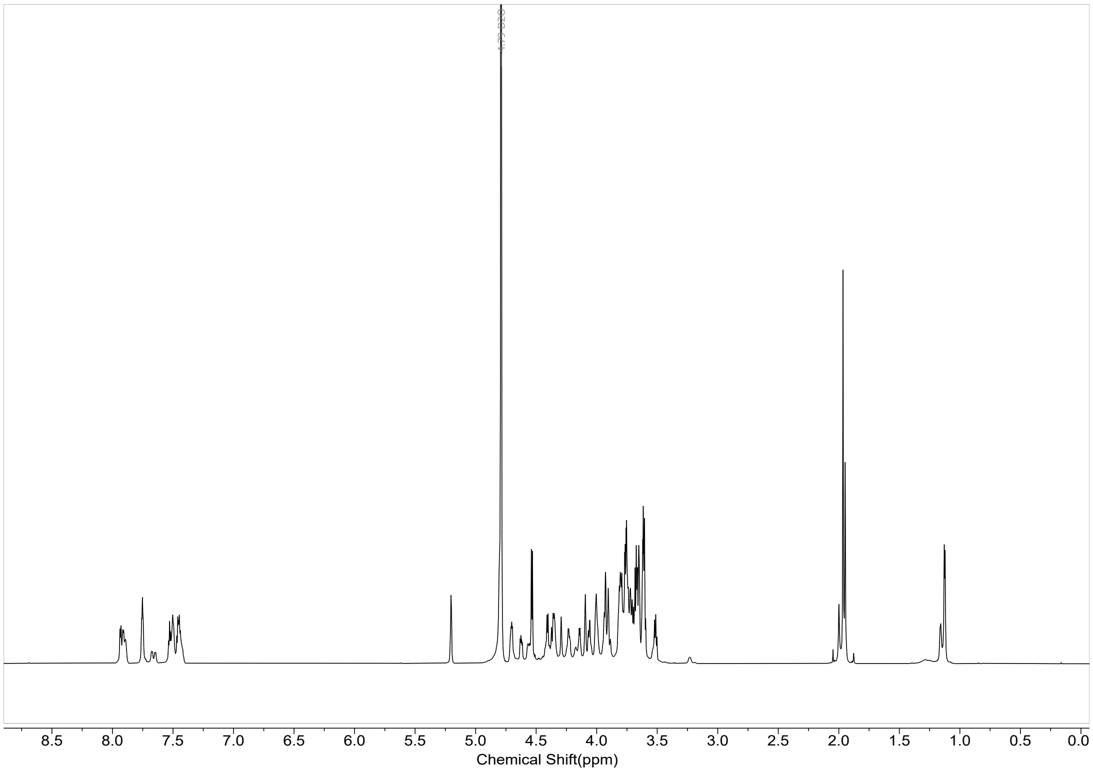

^{1}H NMR of Compound **28**

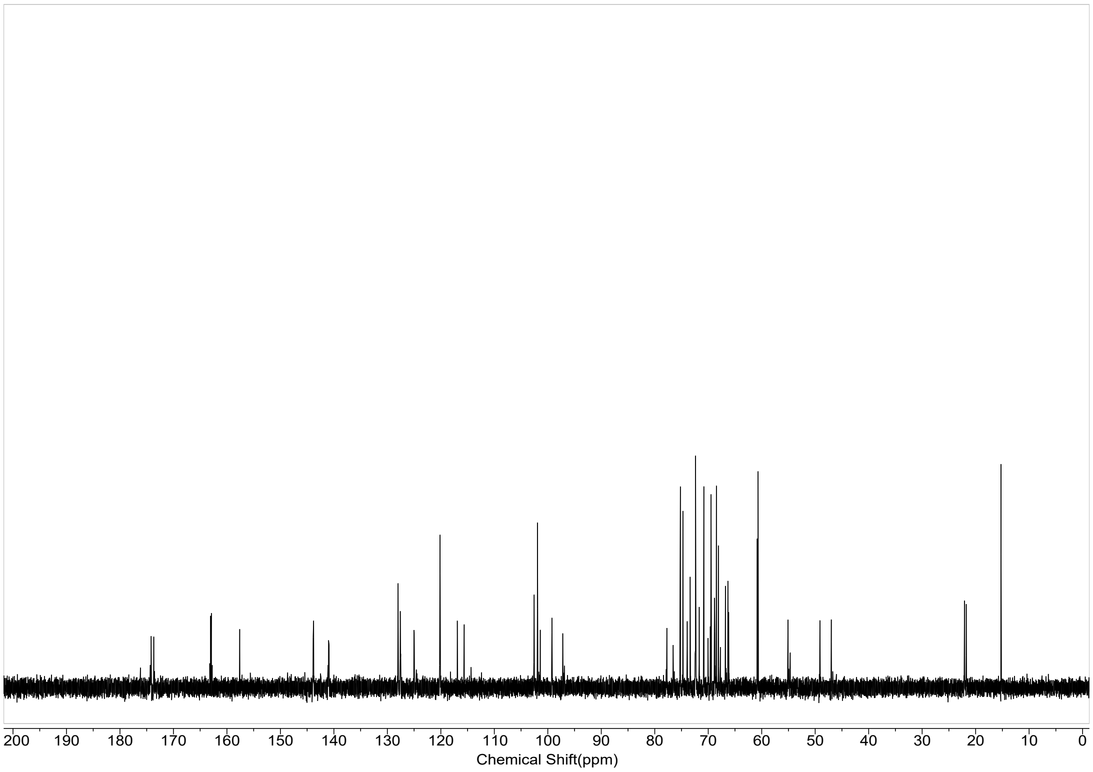

^{13}C NMR of Compound **28**

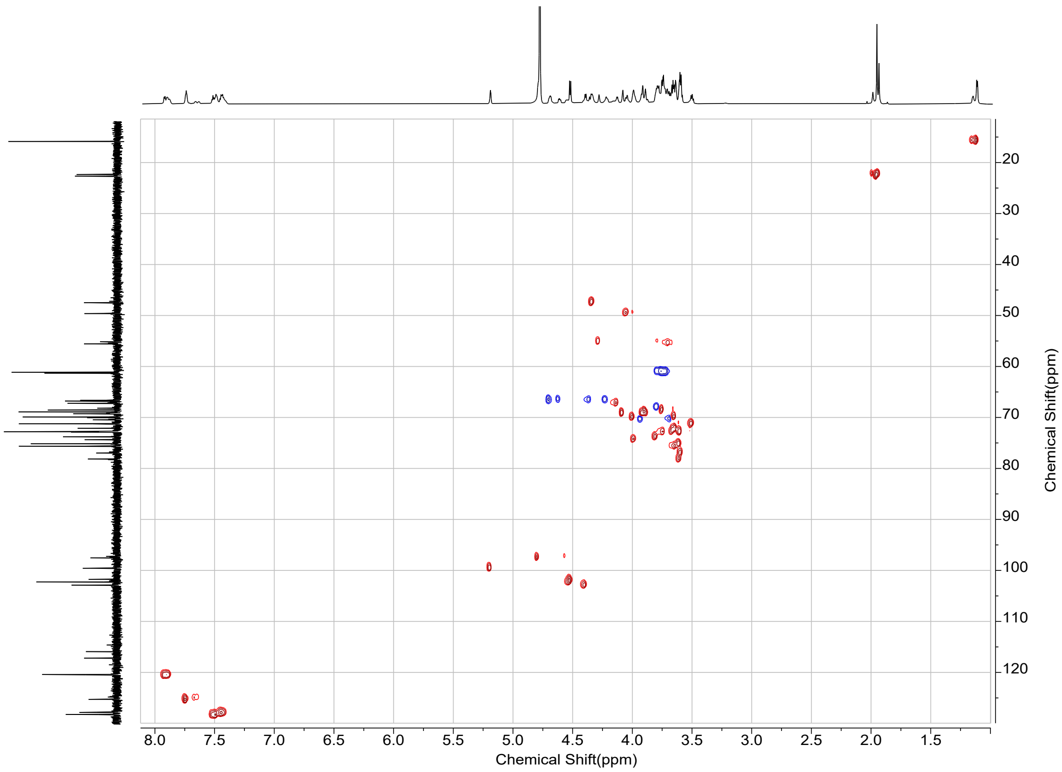

HSQC spectra of Compound **28**

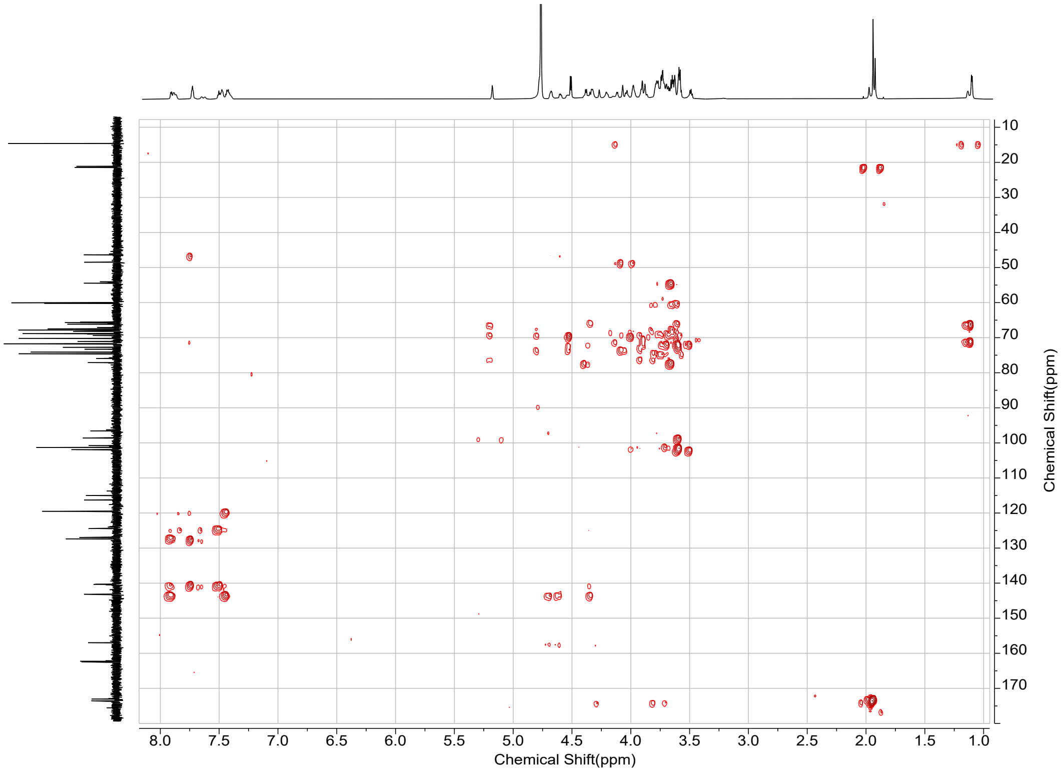

HMBC spectra of Compound **28**

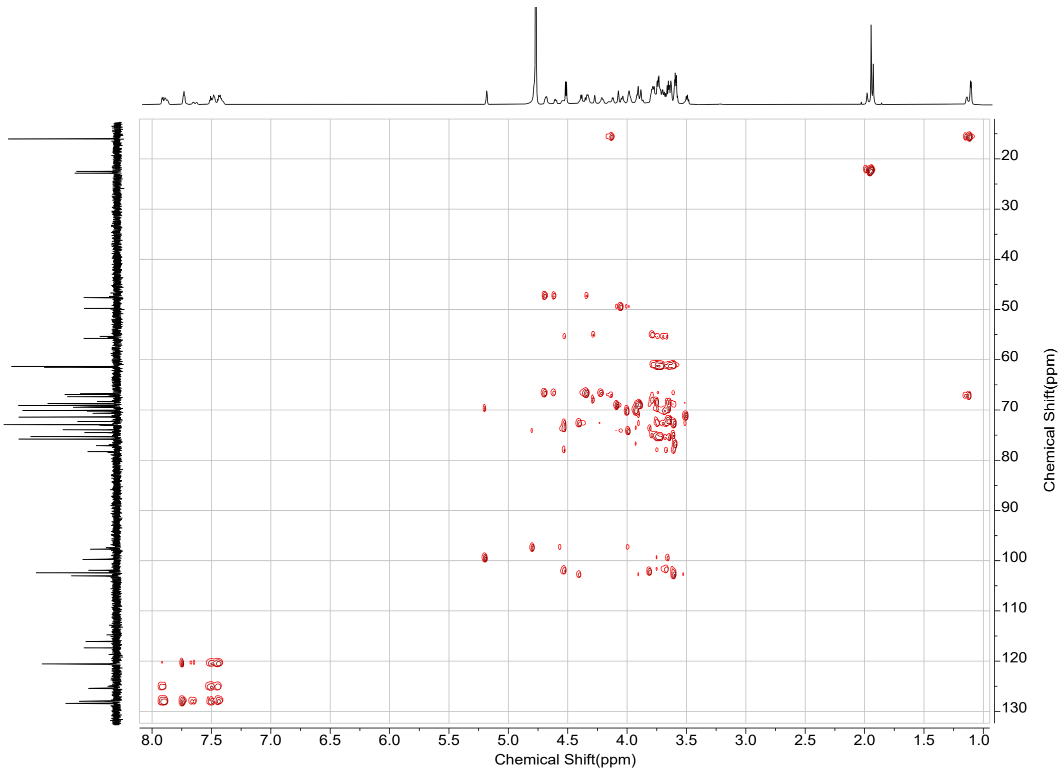

HSQC-TOCSY spectra of Compound **28**

Compound 29

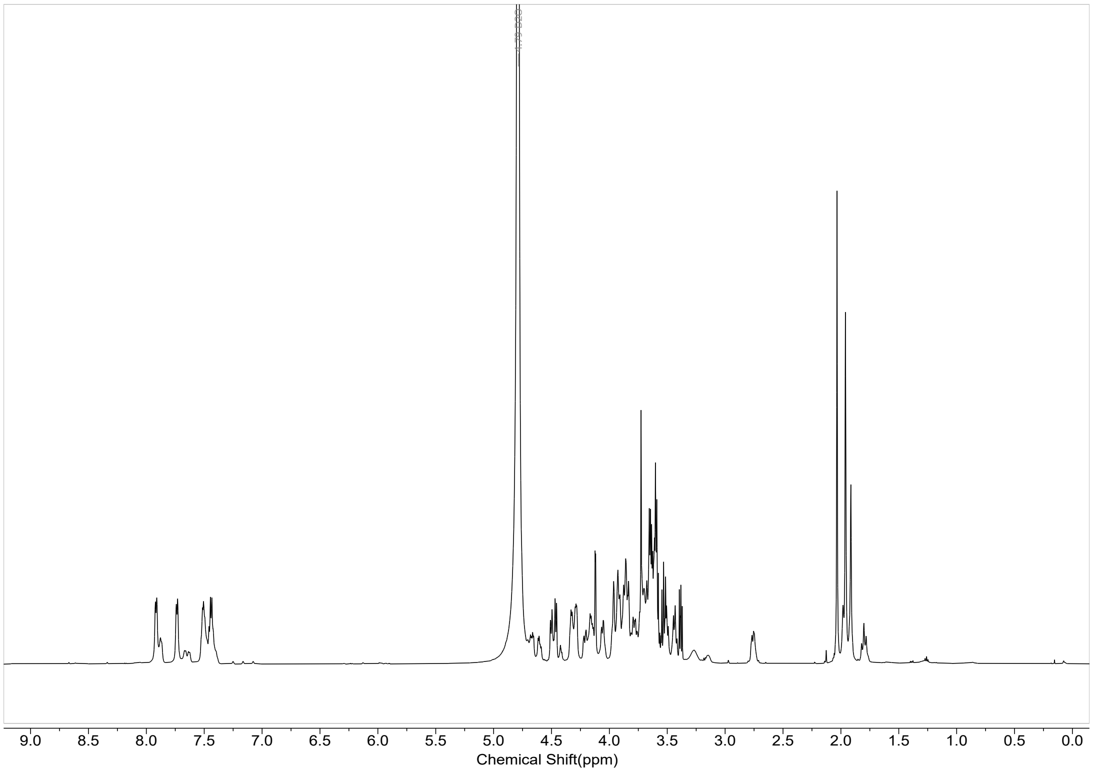

^{1}H NMR of Compound **29**

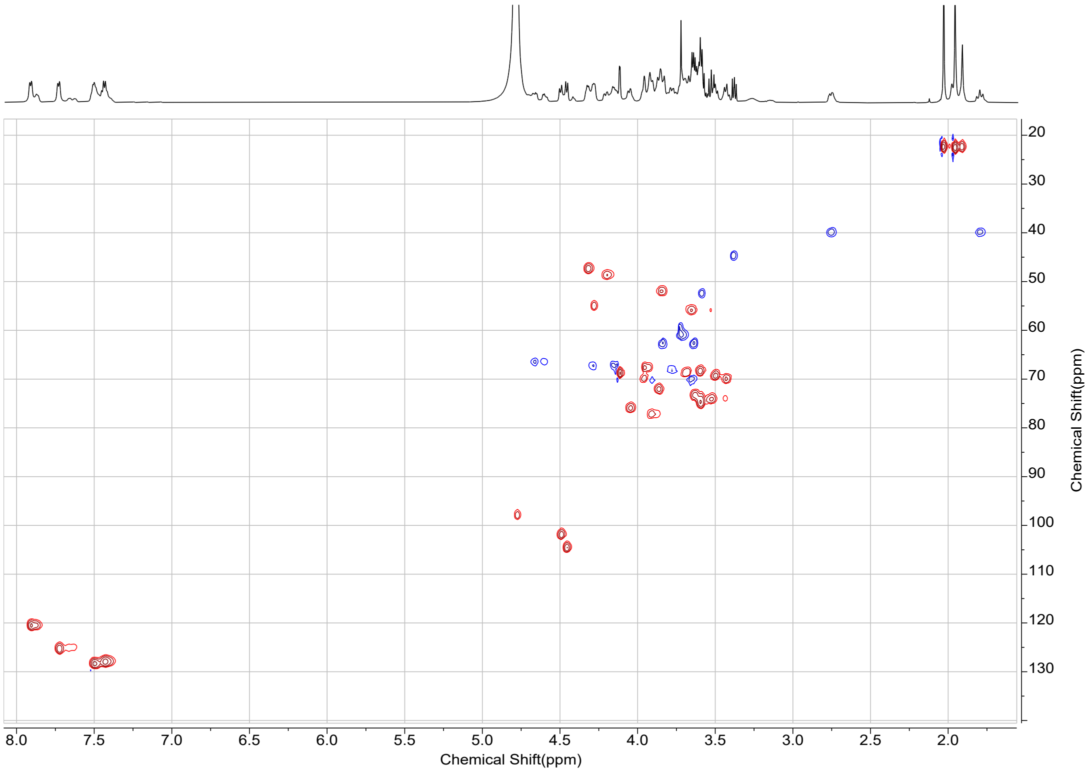

HSQC of Compound **29**

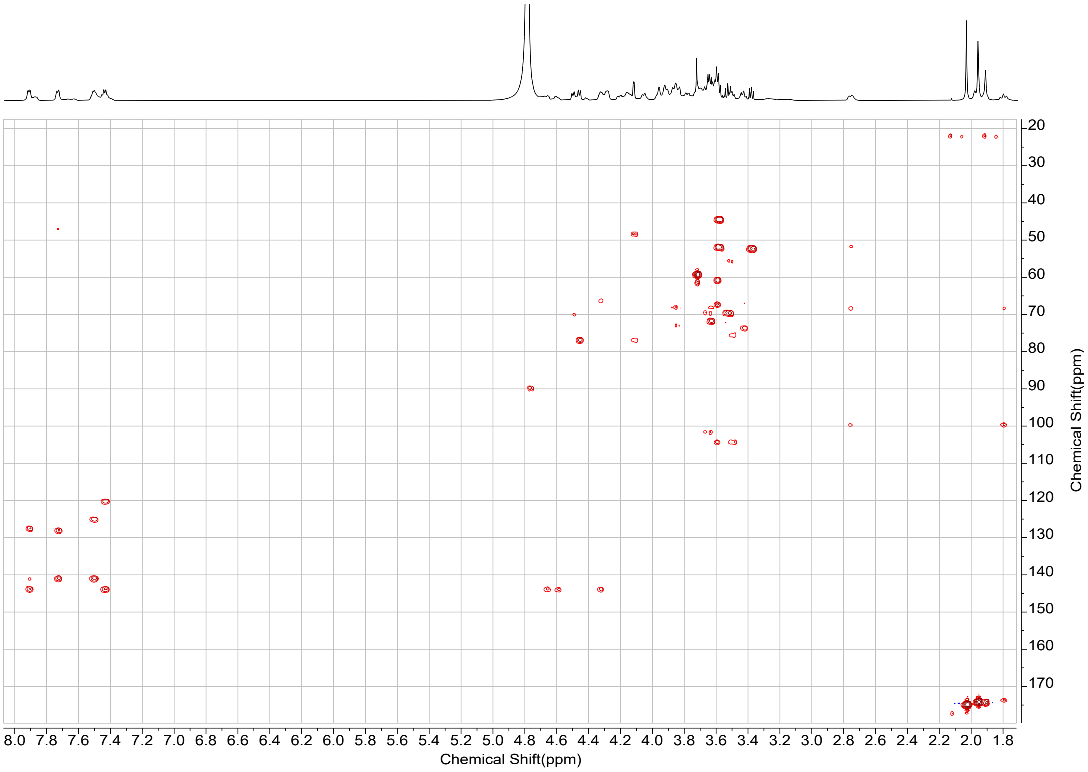

HMBC spectra of Compound **29**

Compound 30

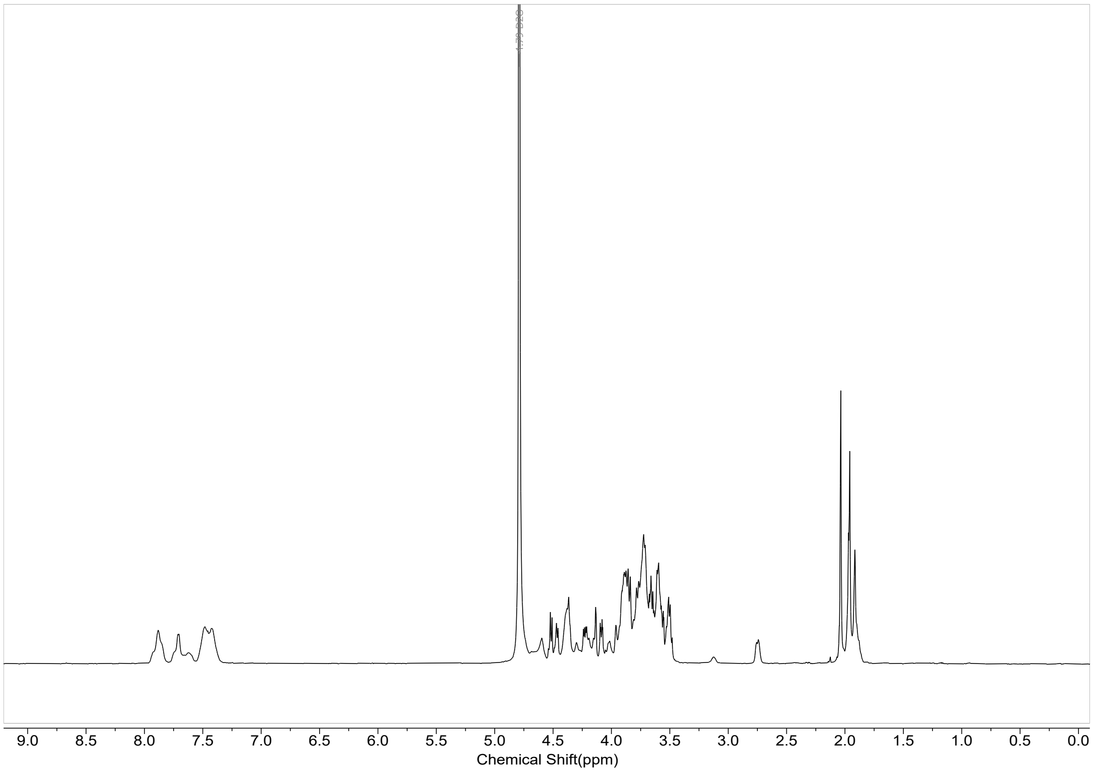

^{1}H NMR of Compound **30**

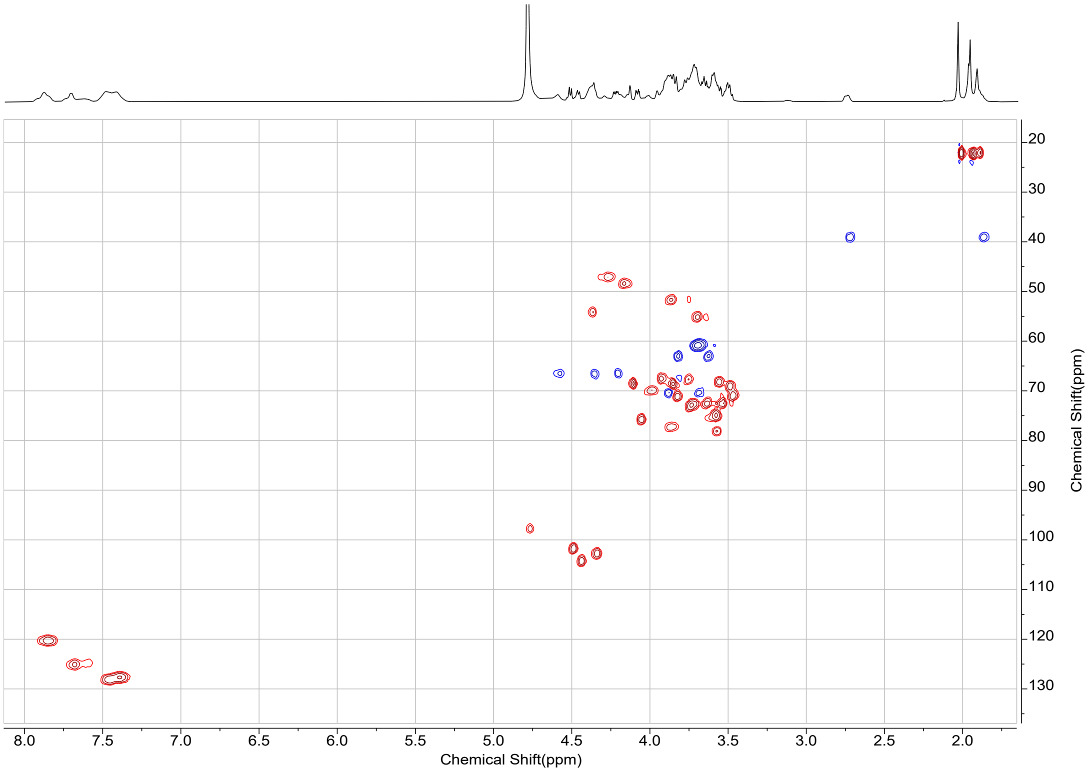

HSQC spectra of Compound **30**

Compound 31

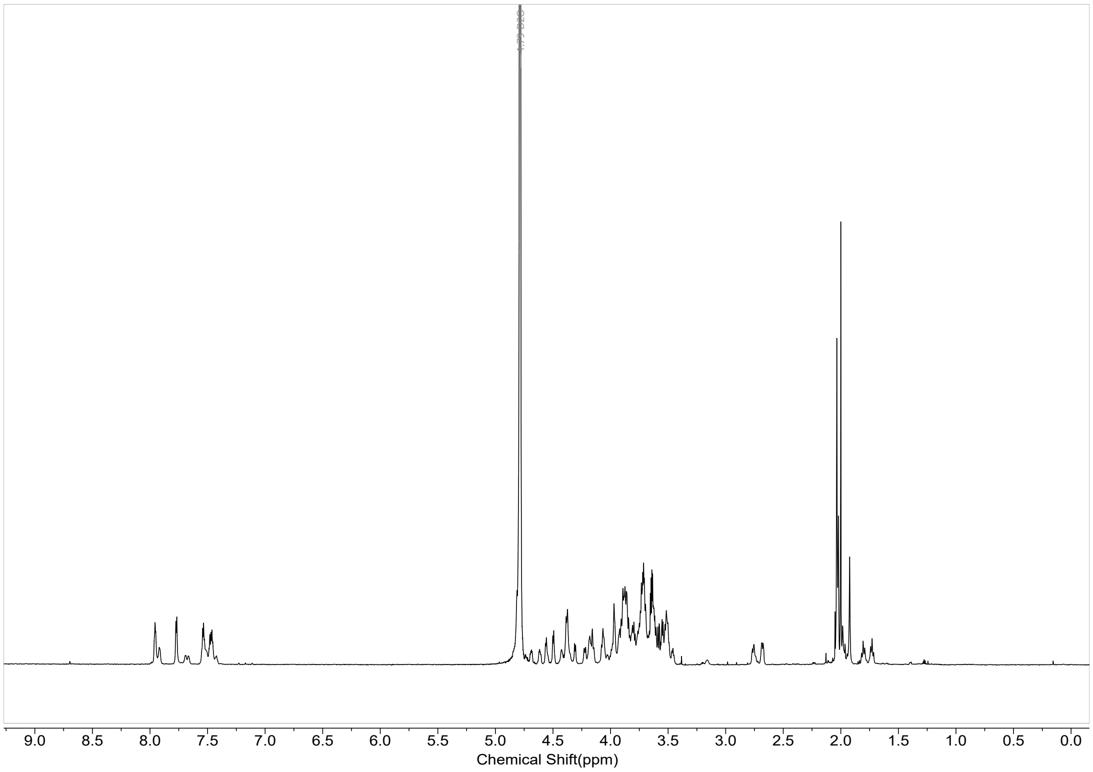

^{1}H NMR of Compound **31**

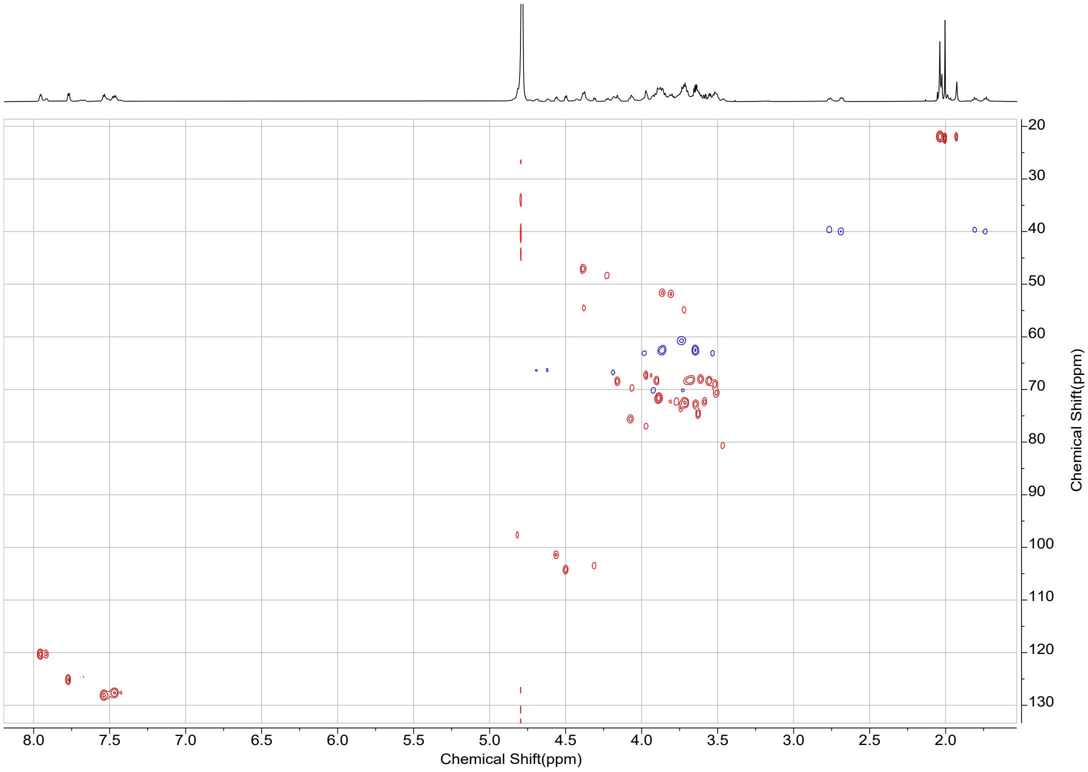

HSQC spectra of Compound **31**

Compound 32

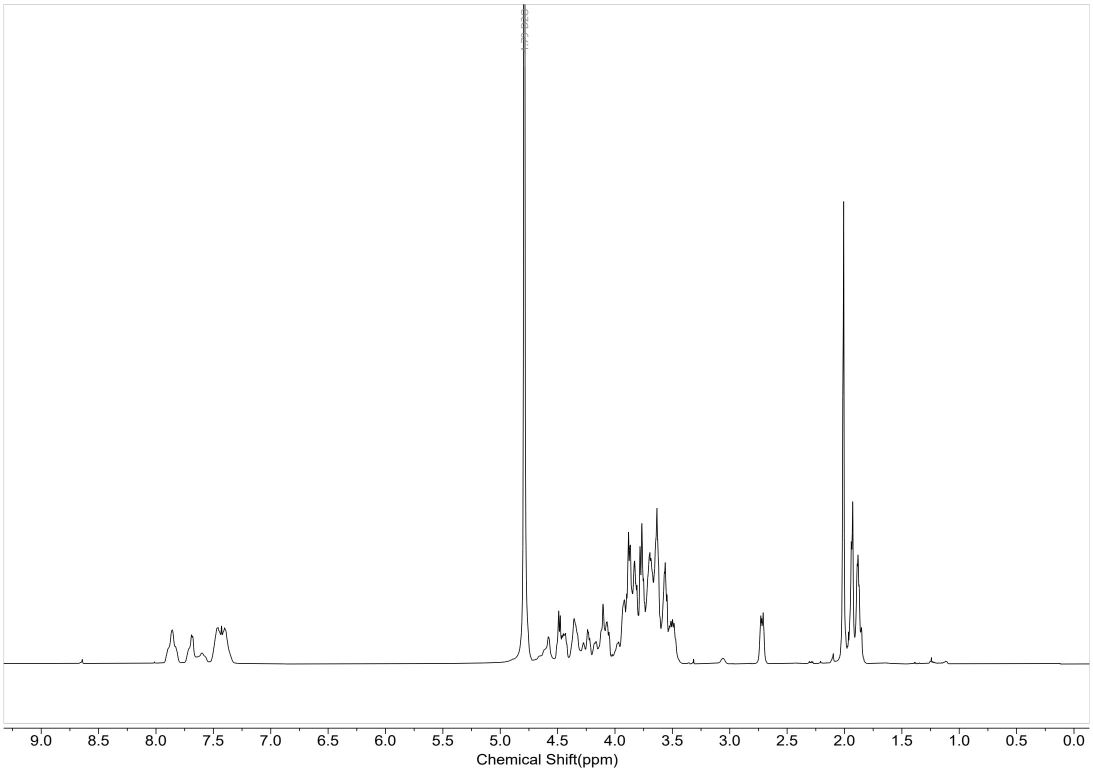

^{1}H NMR of Compound **32**

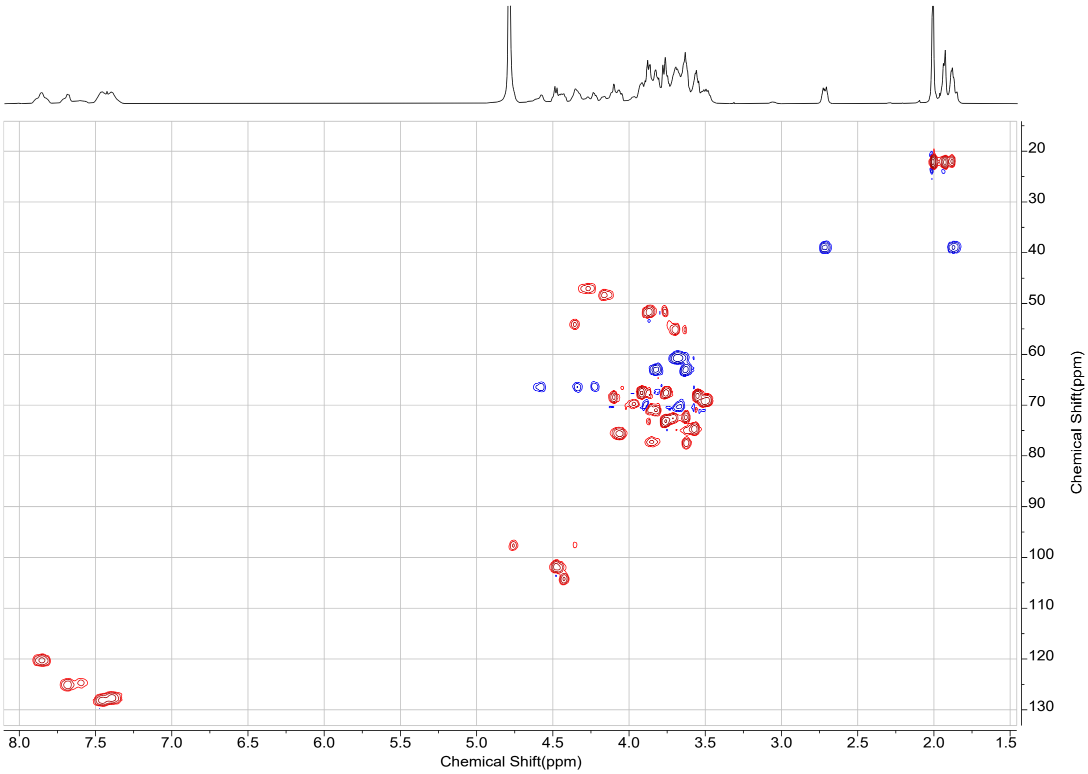

HSQC spectra of Compound **32**

Compound 33

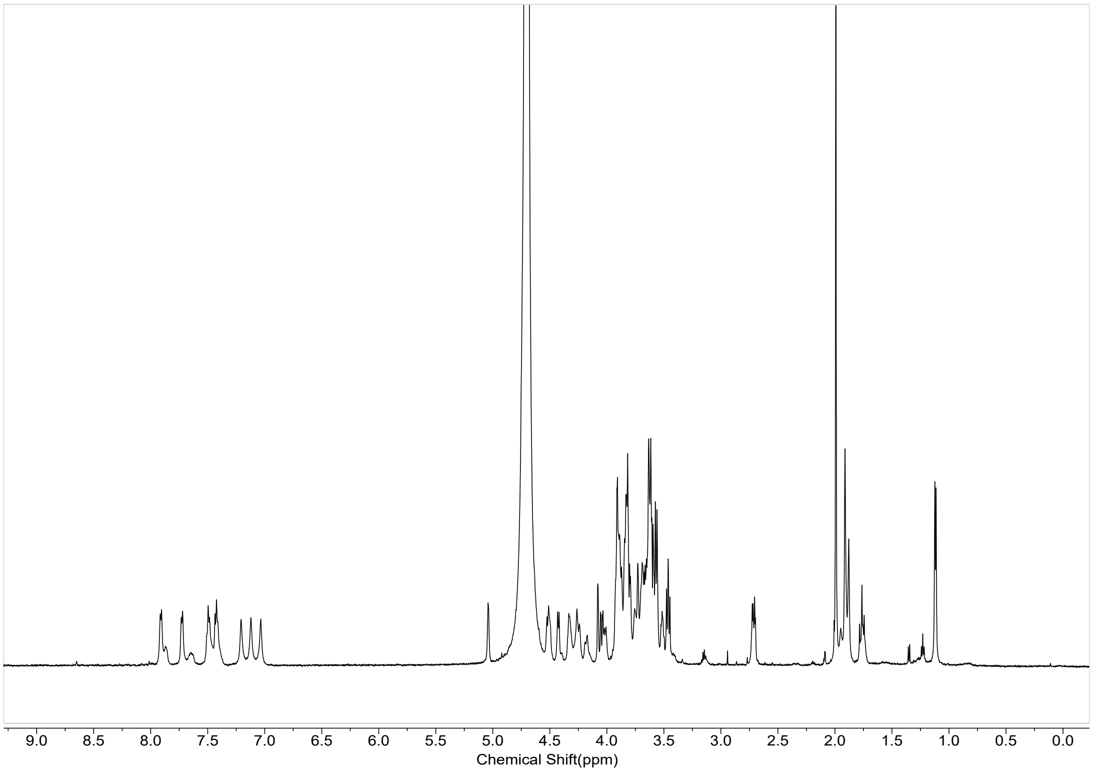

^{1}H NMR of Compound **33**

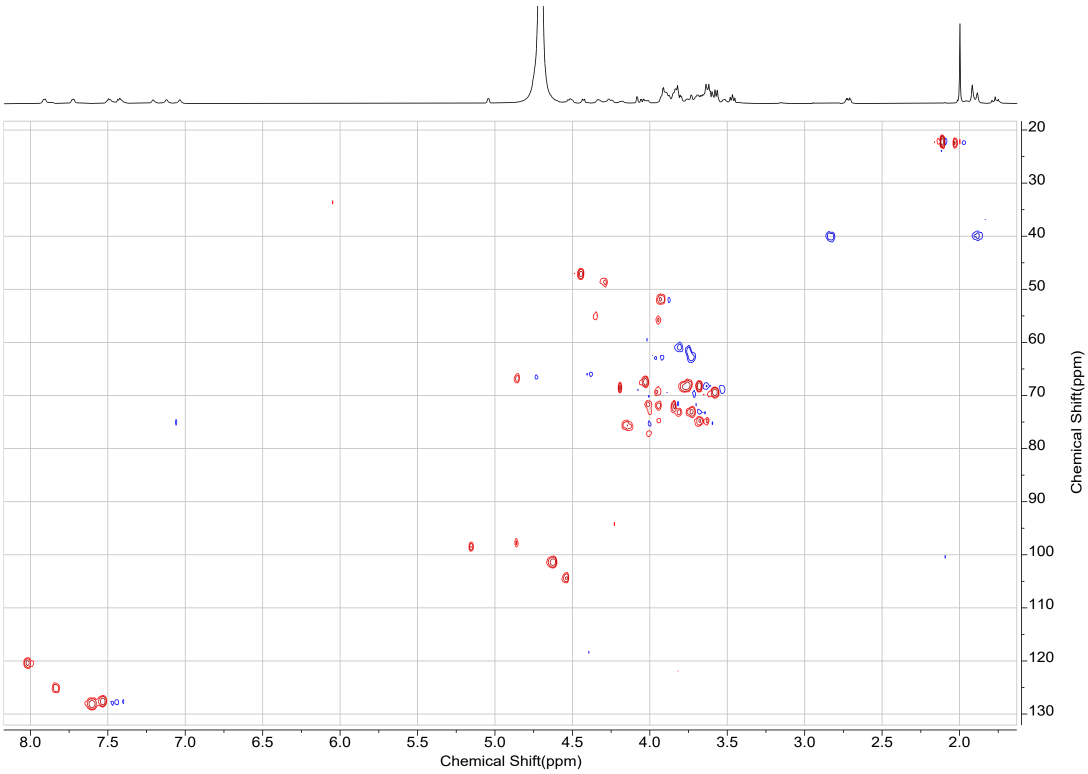

HSQC spectra of Compound **33**

Compound 34

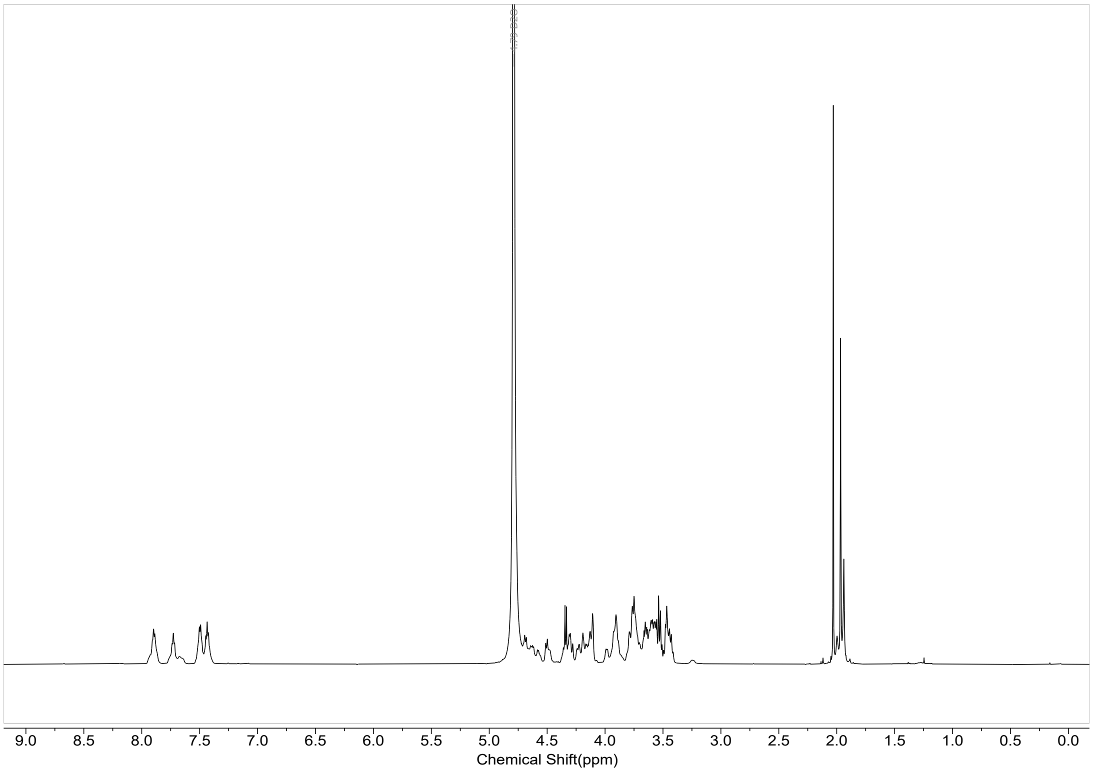

^{1}H NMR of Compound **34**

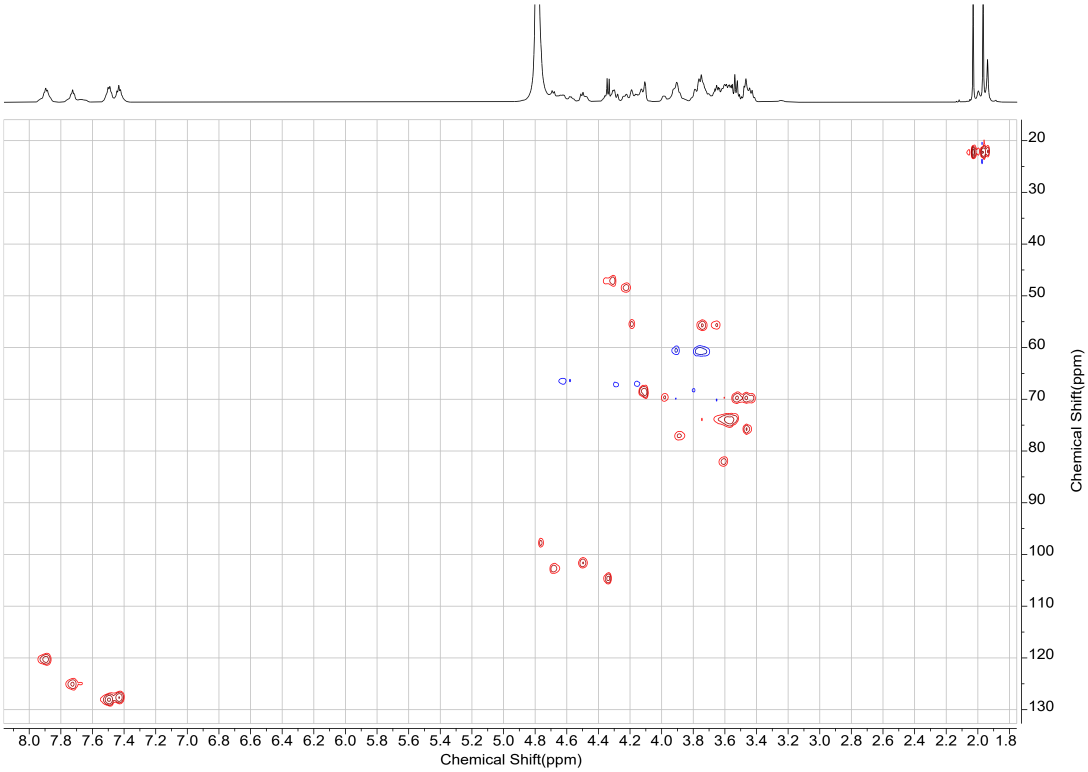

HSQC spectra of Compound **34**

Compound 35

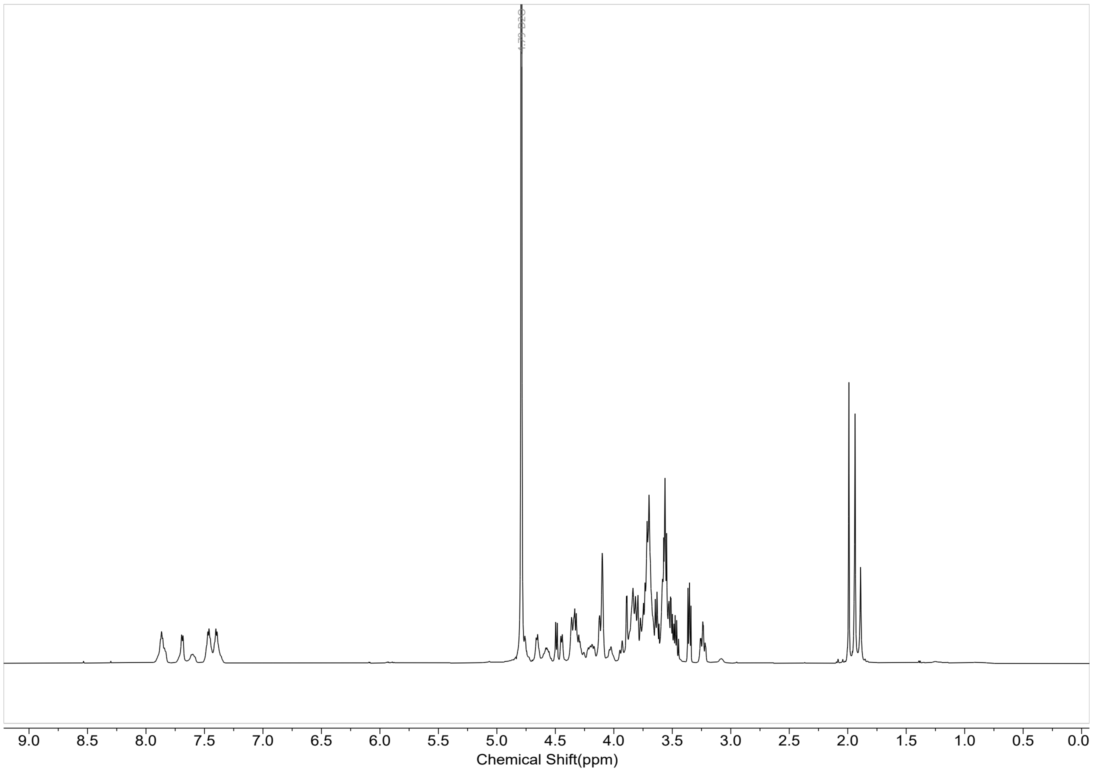

^{1}H NMR of Compound **35**

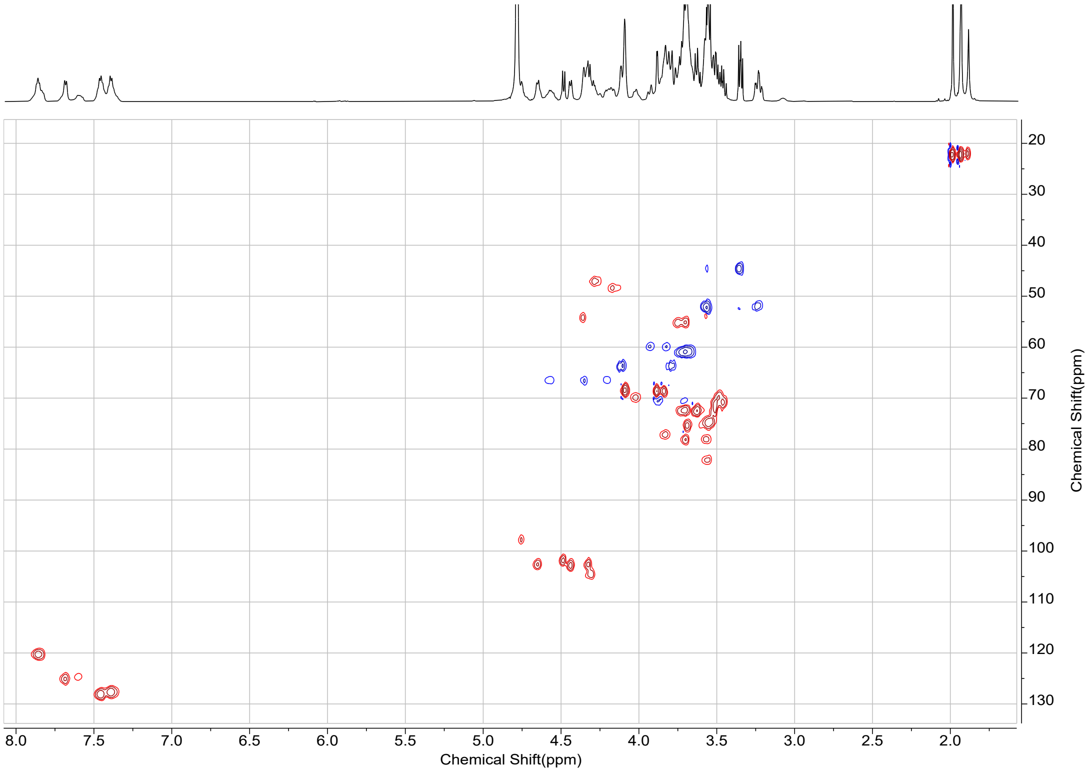

HSQC spectra of Compound **35**

Compound 36

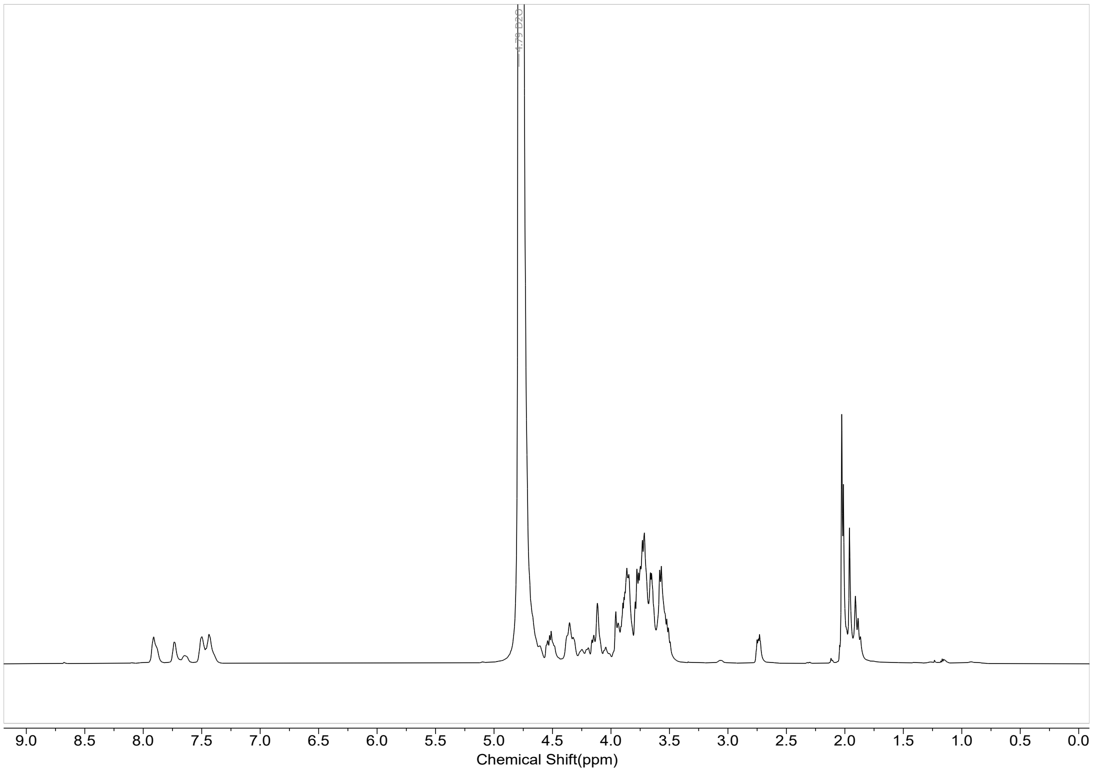

^{1}H NMR of Compound **36**

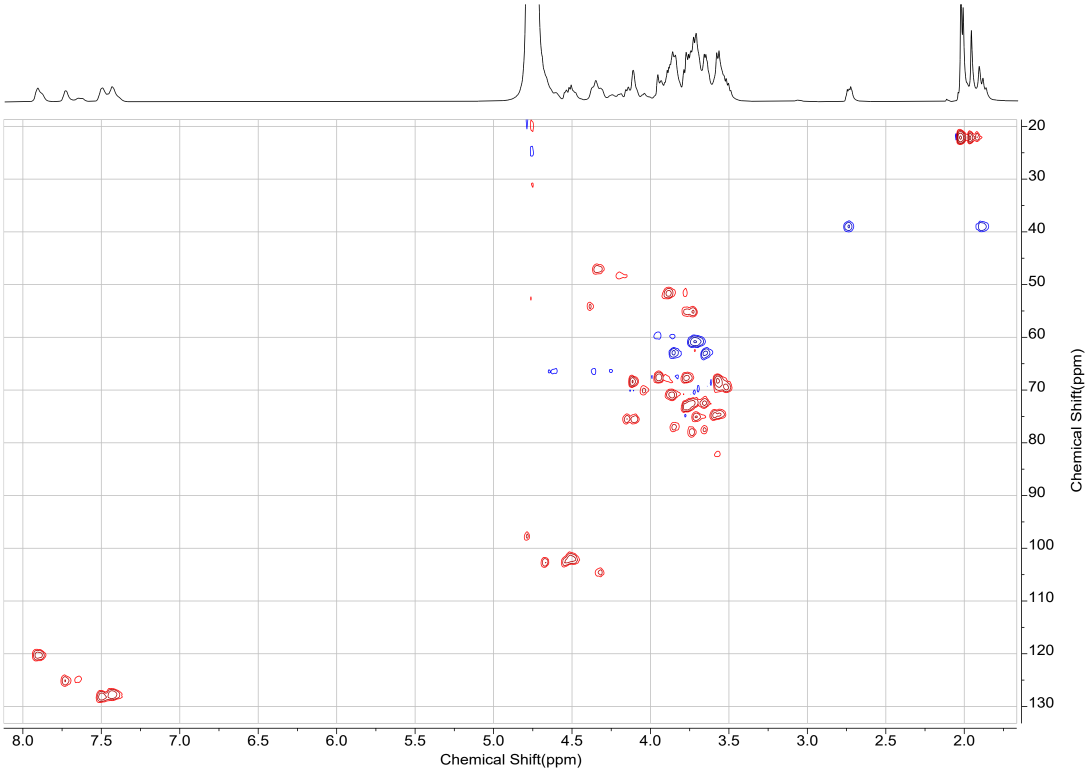

HSQC spectra of Compound **36**

Compound 37

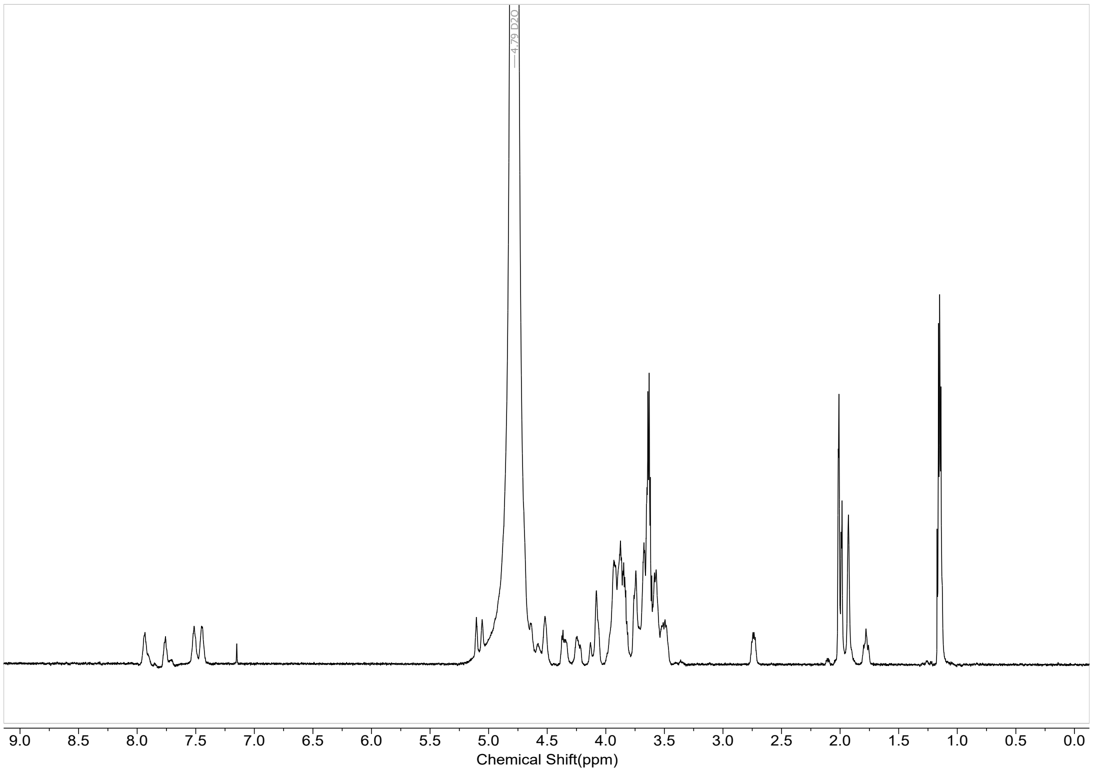

^{1}H NMR of Compound **37**

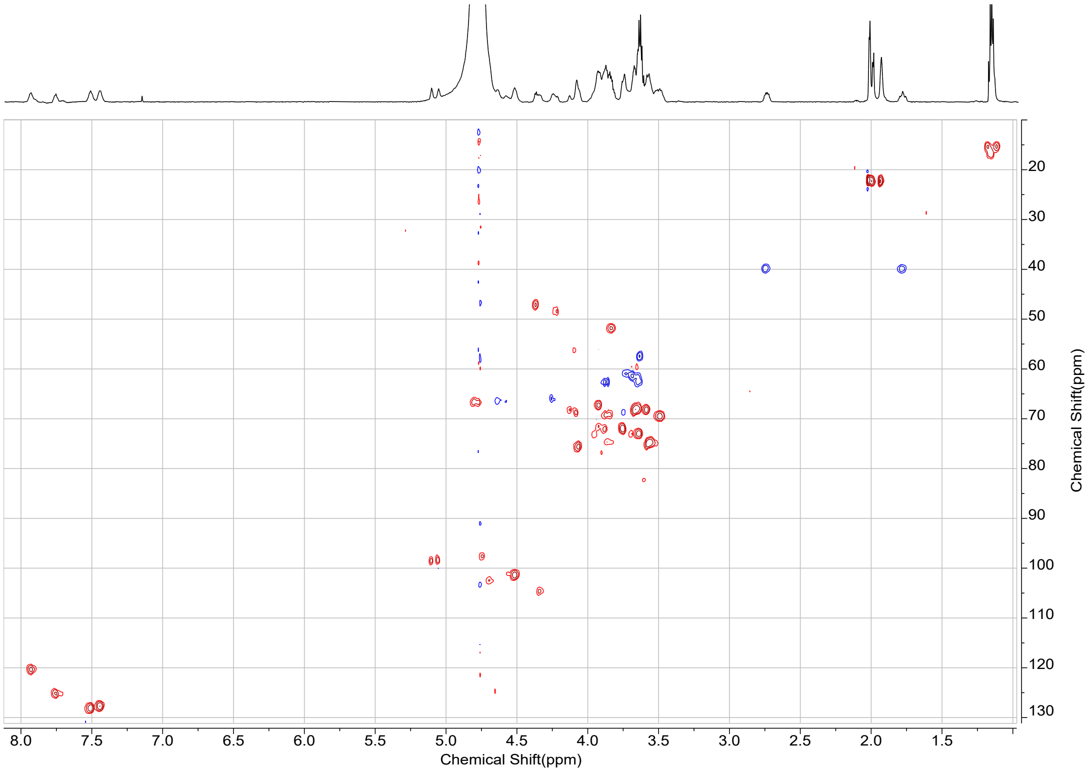

HSQC spectra of Compound **37**

Compound 38

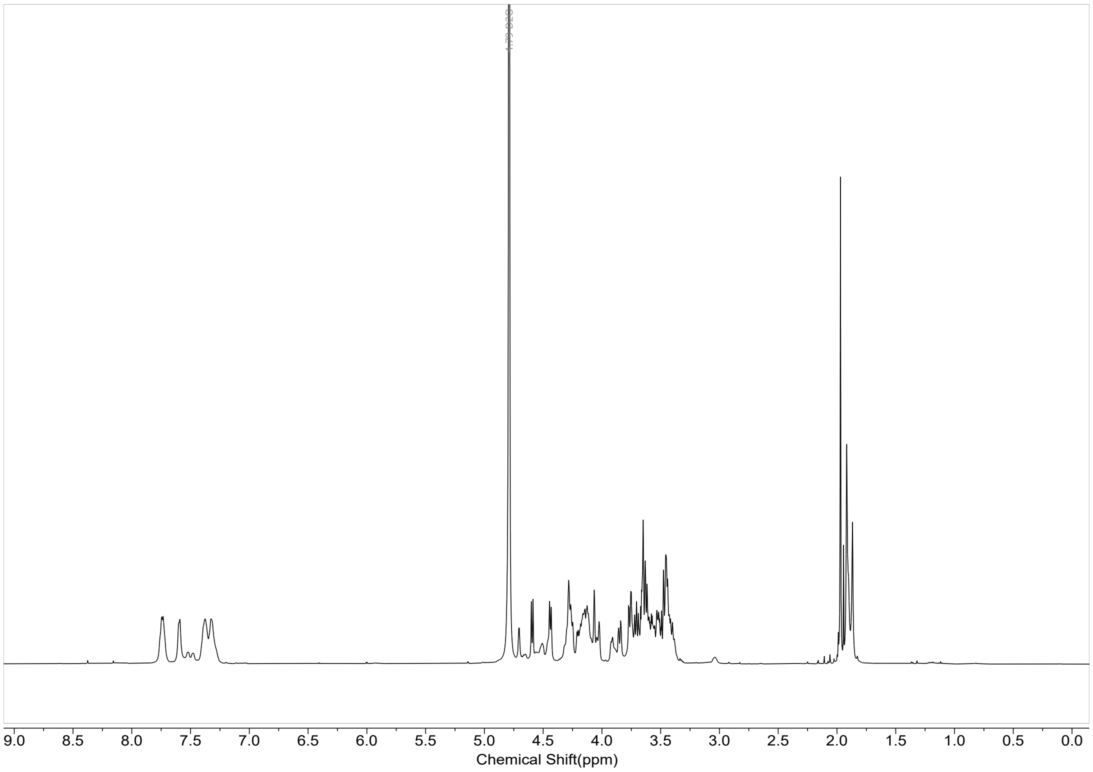

^{1}H NMR of Compound **38**

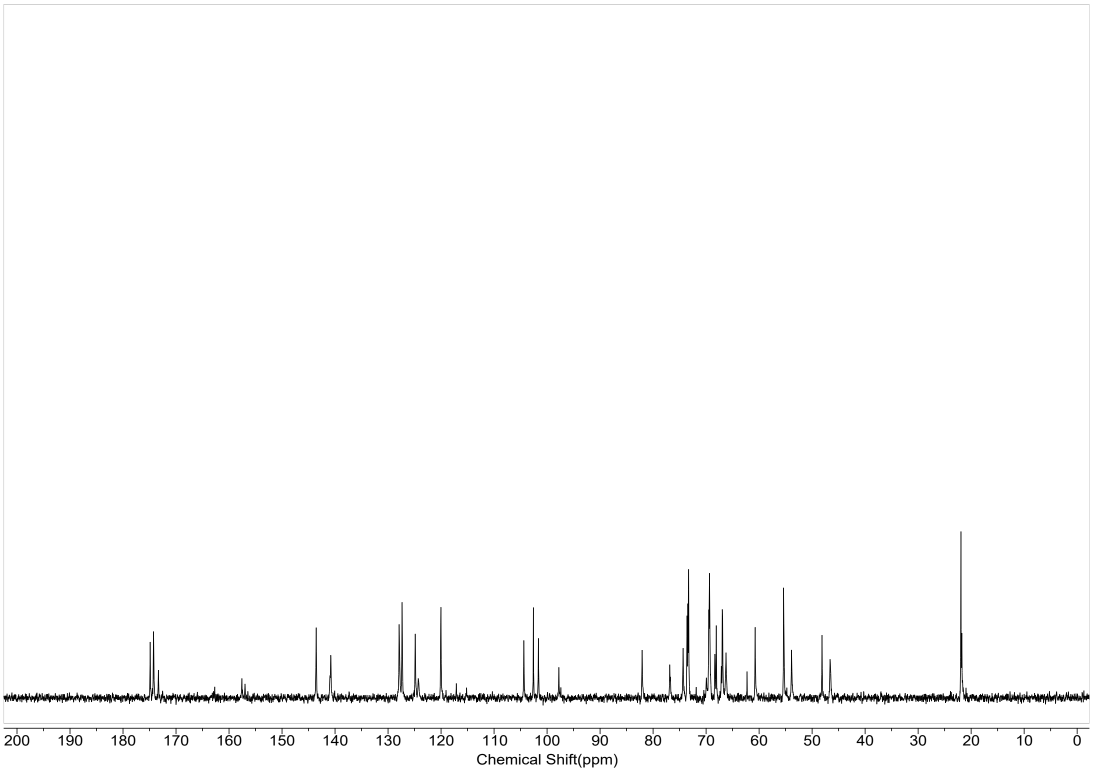

^{13}C NMR of Compound **38**

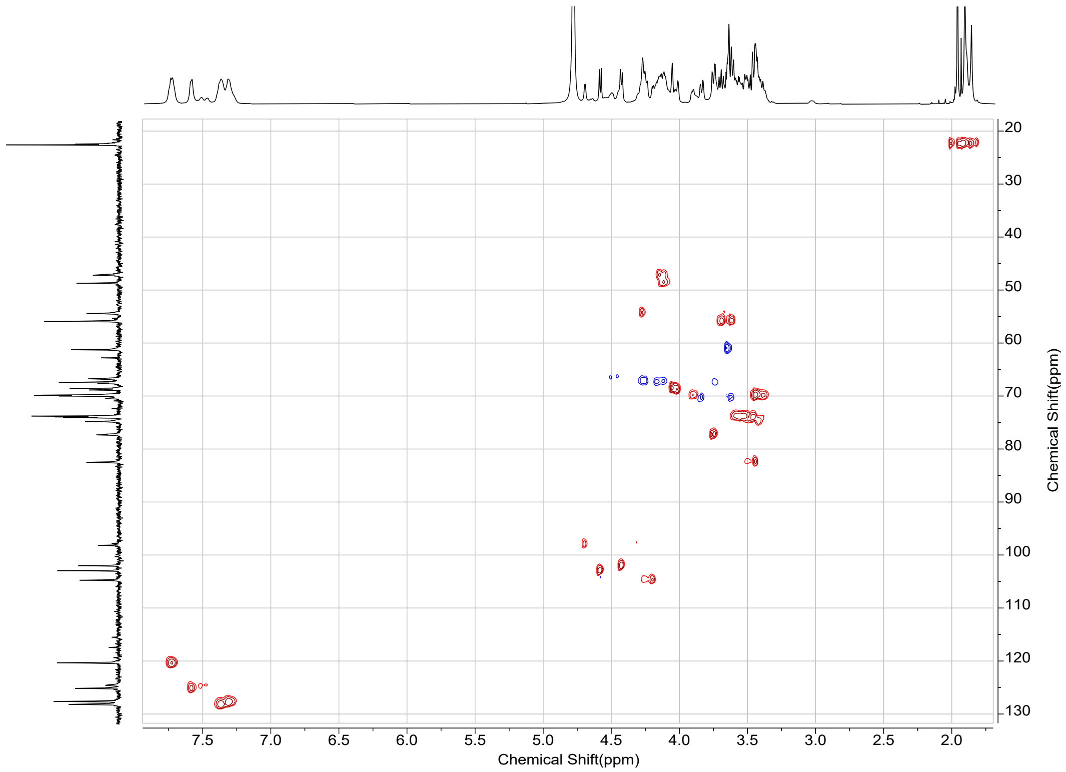

HSQC spectra of Compound **38**

Compound 39

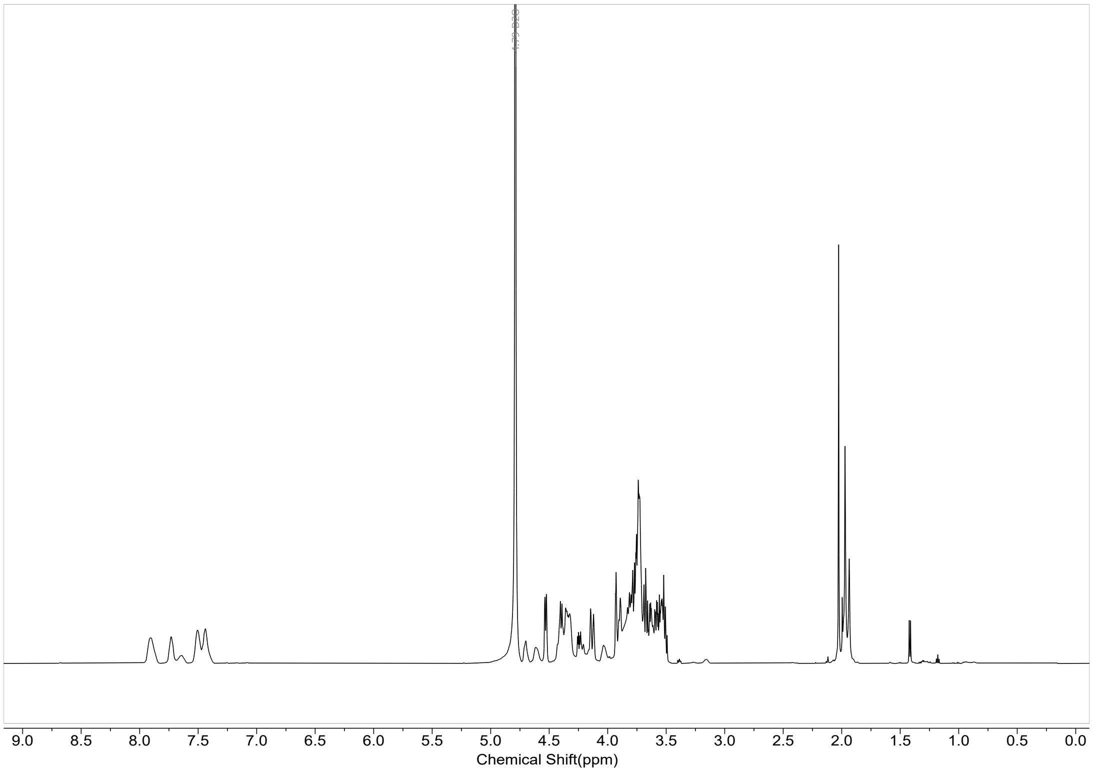

^{1}H NMR of Compound **39**

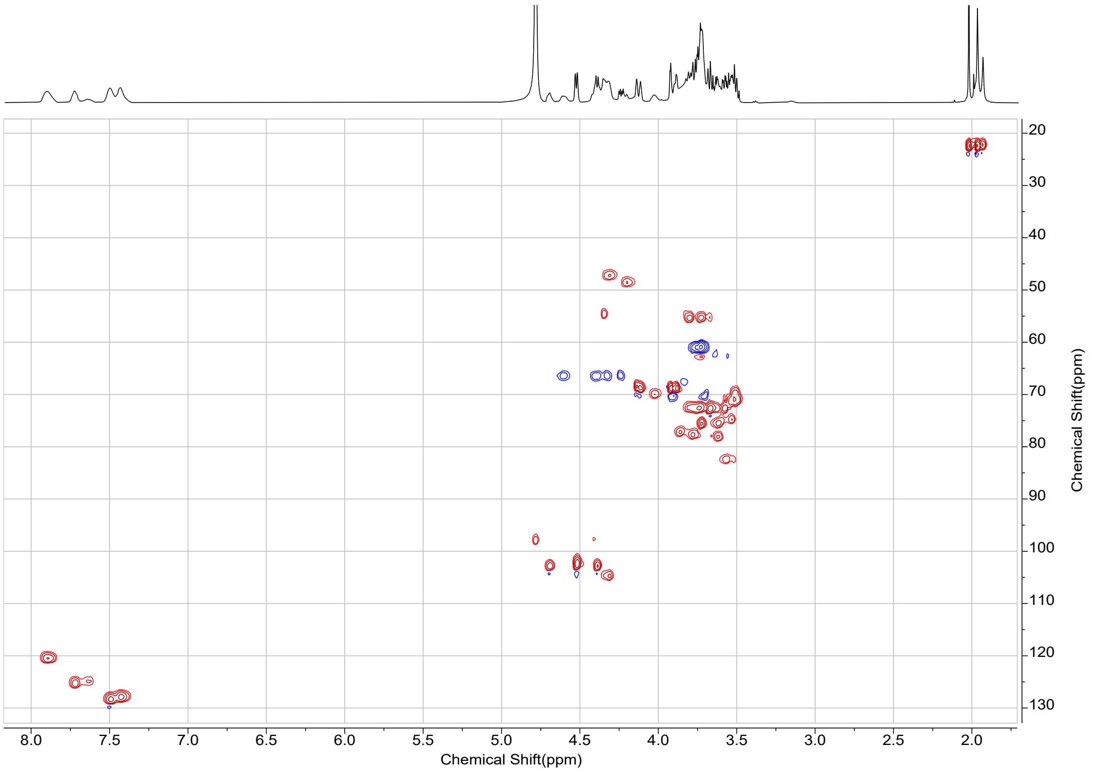

HSQC spectra of Compound **39**

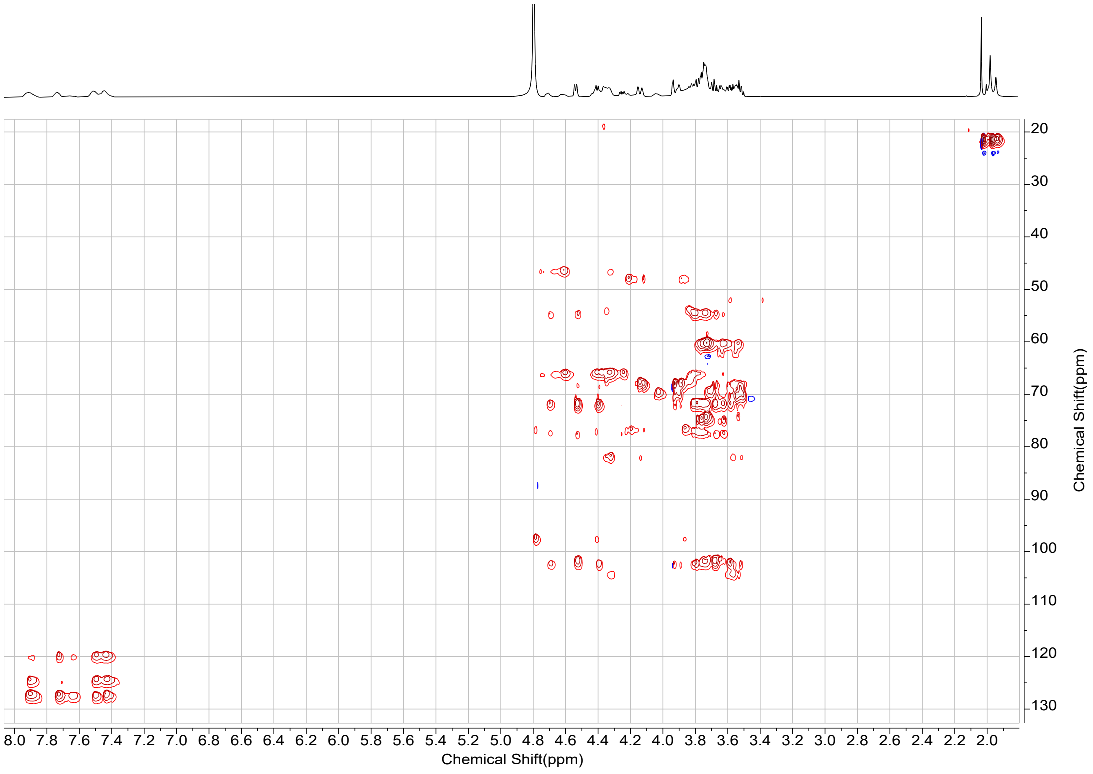

HSQC-TOCSY spectra of Compound **39**

Compound 40

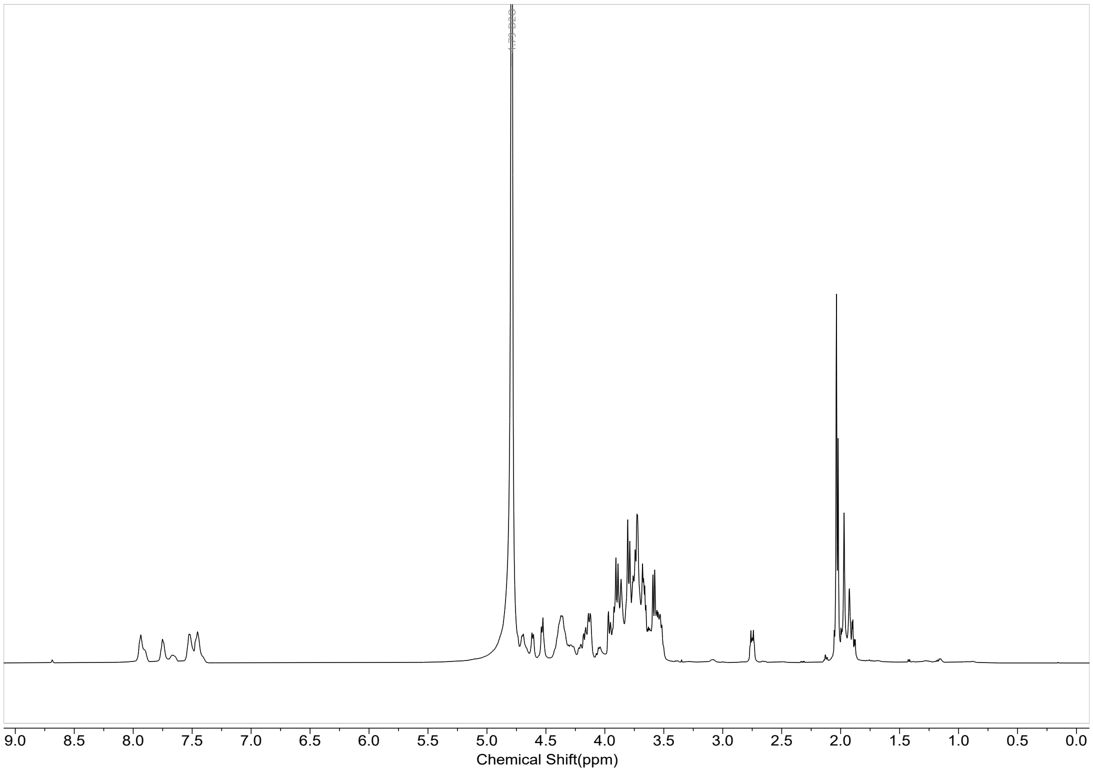

^{1}H NMR of Compound **40**

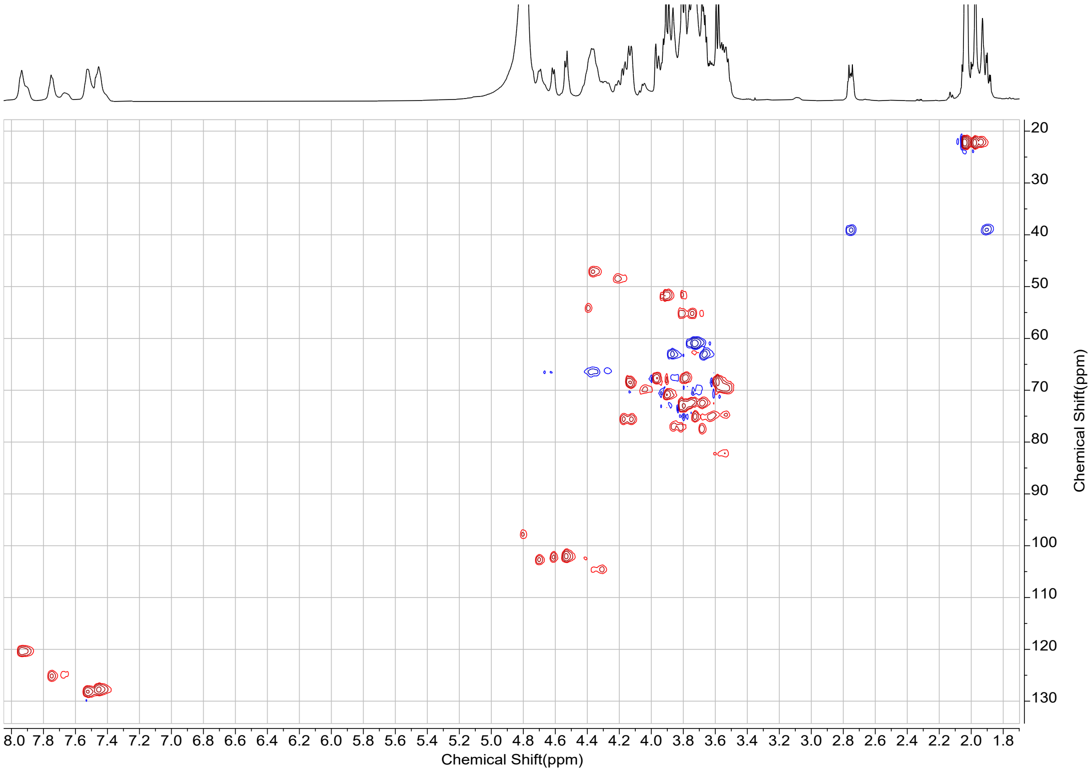

HSQC spectra of Compound **40**

Compound 41

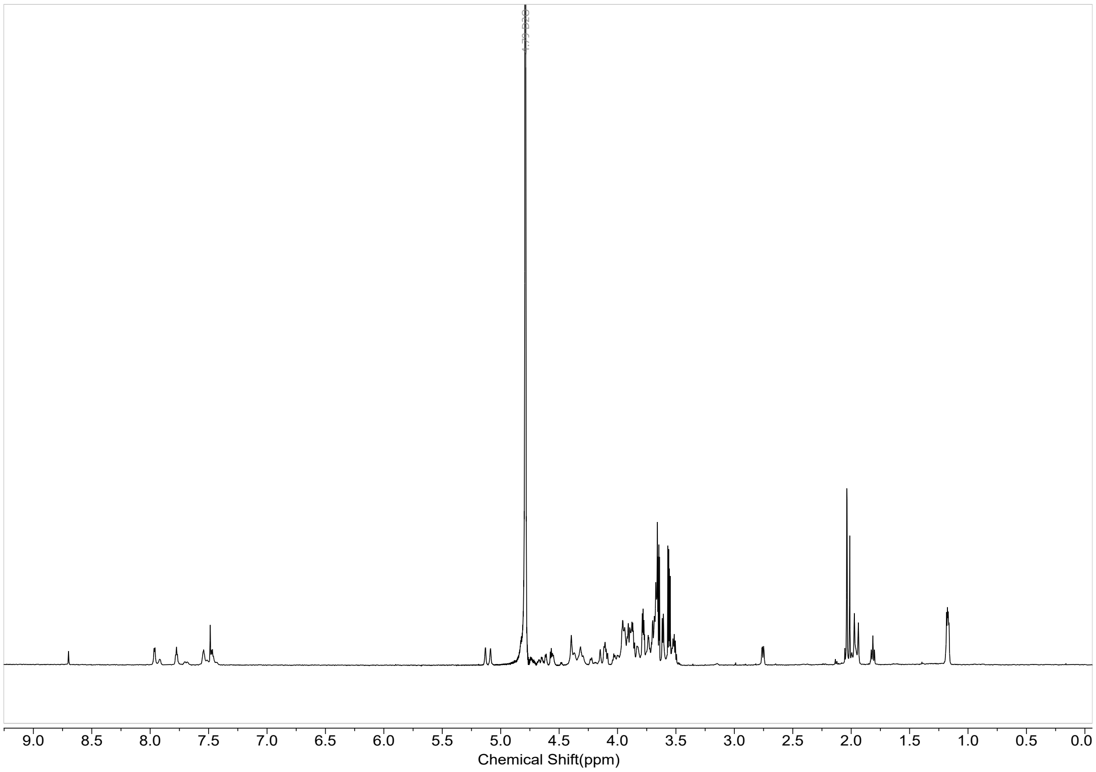

^{1}H NMR of Compound **41**

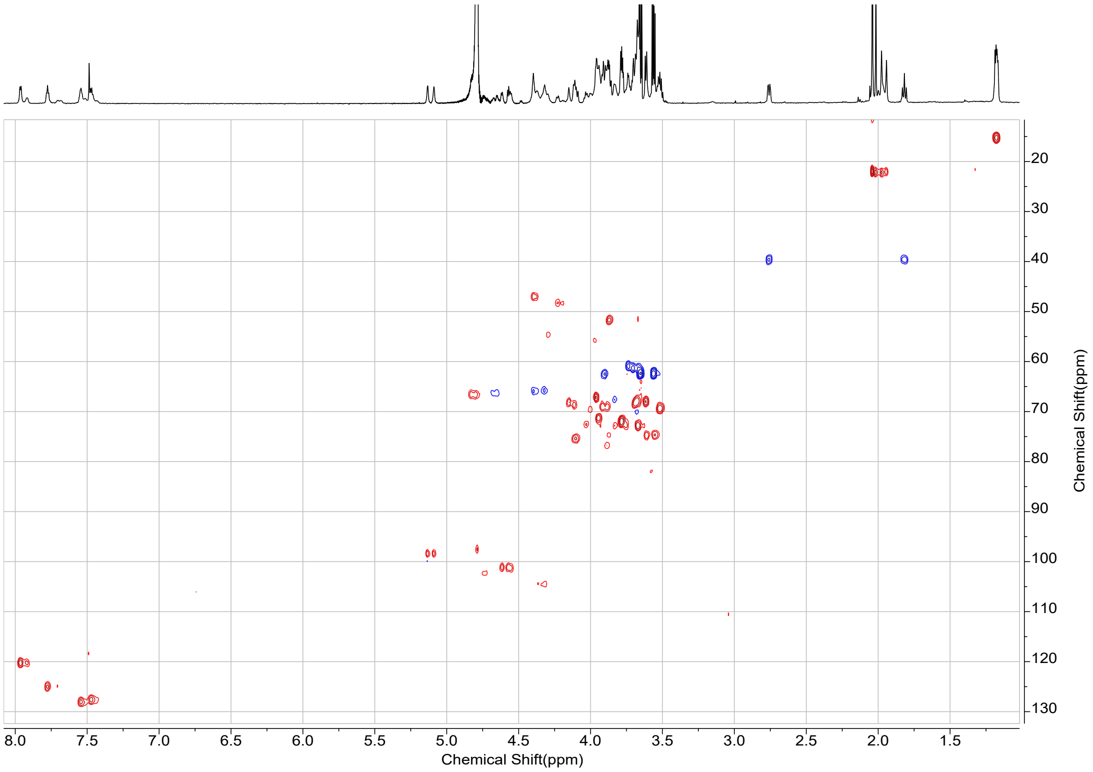

HSQC spectra of Compound **41**

Compound 42

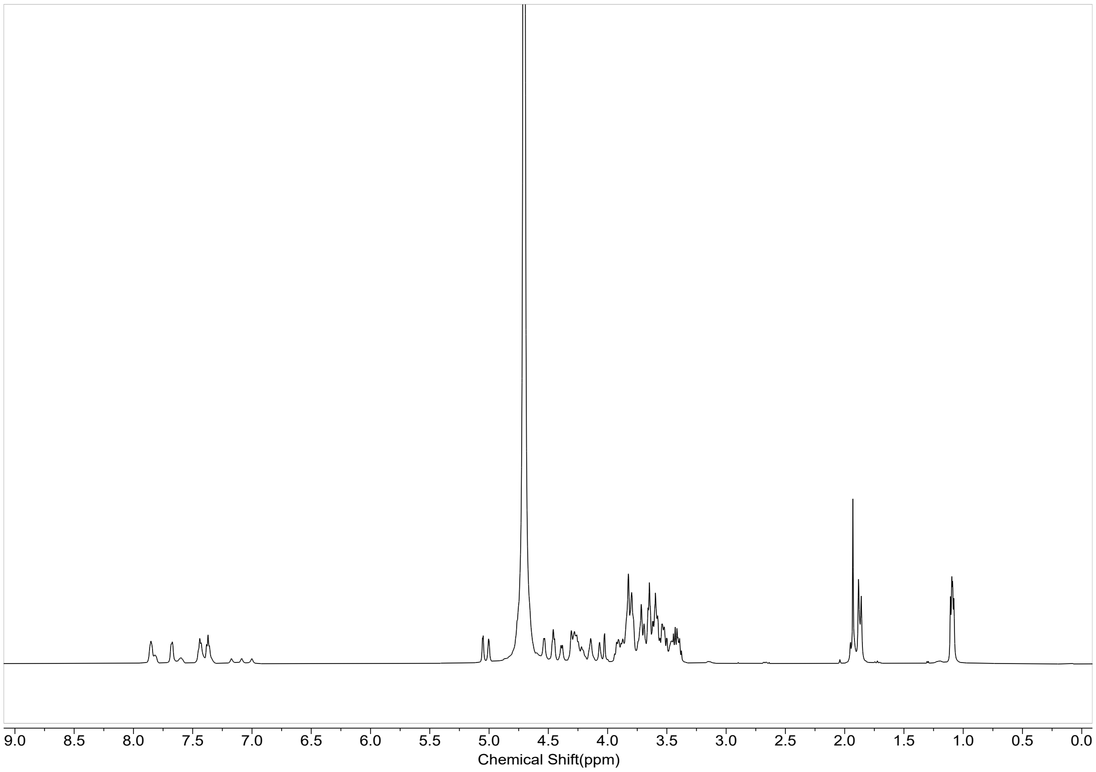

^{1}H NMR of Compound **42**

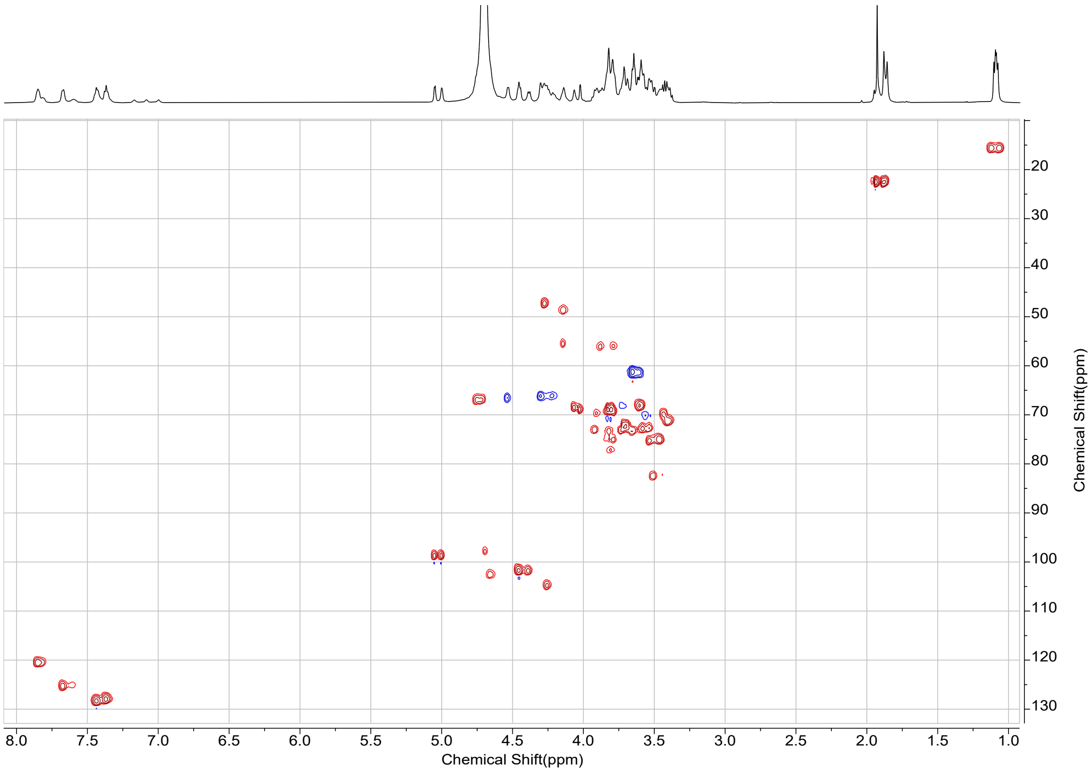

HSQC spectra of Compound **42**

Compound 43

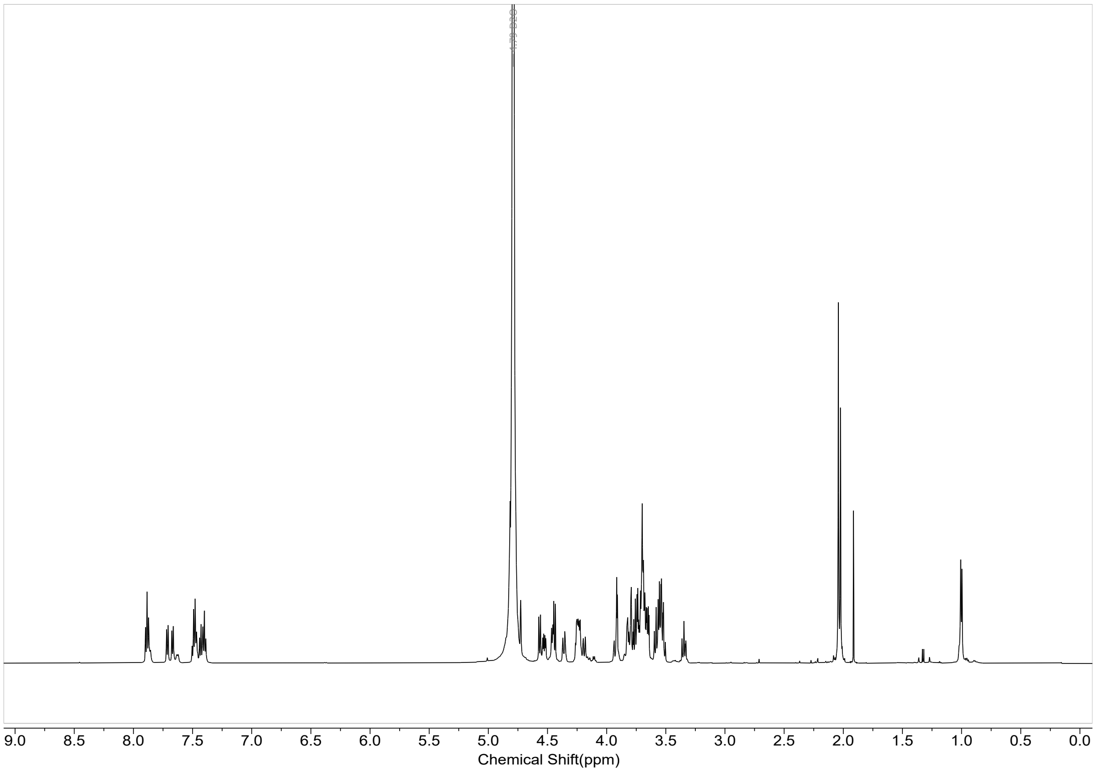

^{1}H NMR of Compound **43**

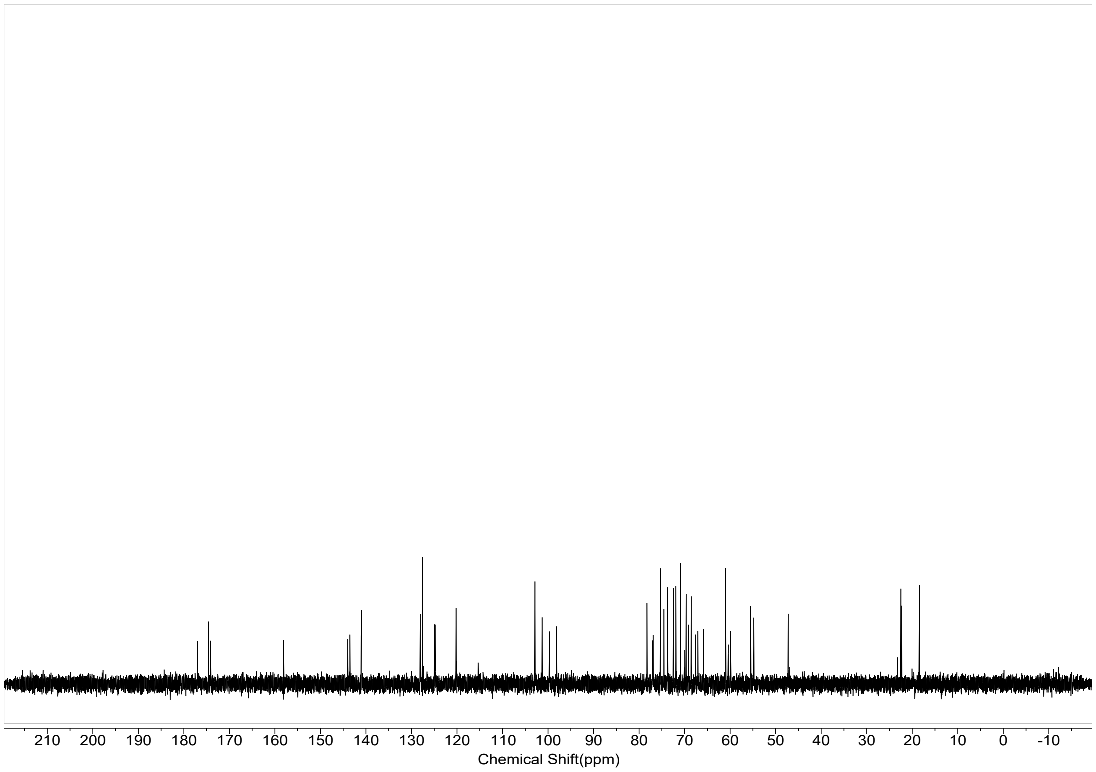

^{13}C NMR of Compound **43**

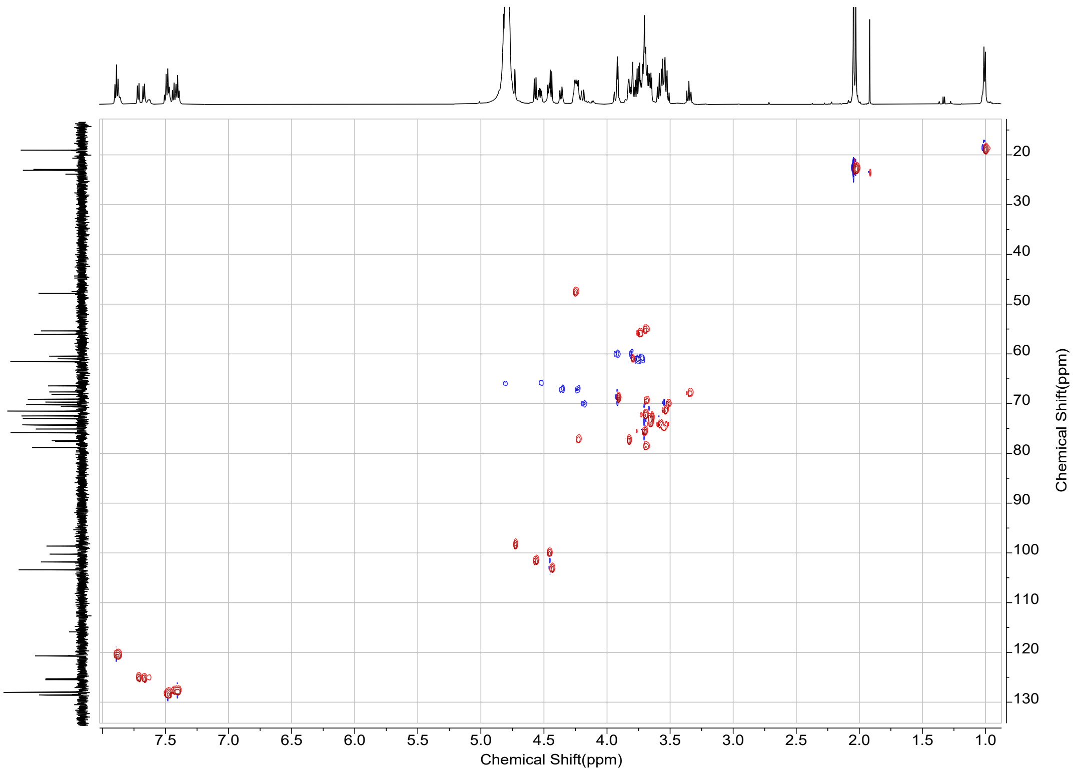

HSQC spectra of Compound **43**

Compound 44

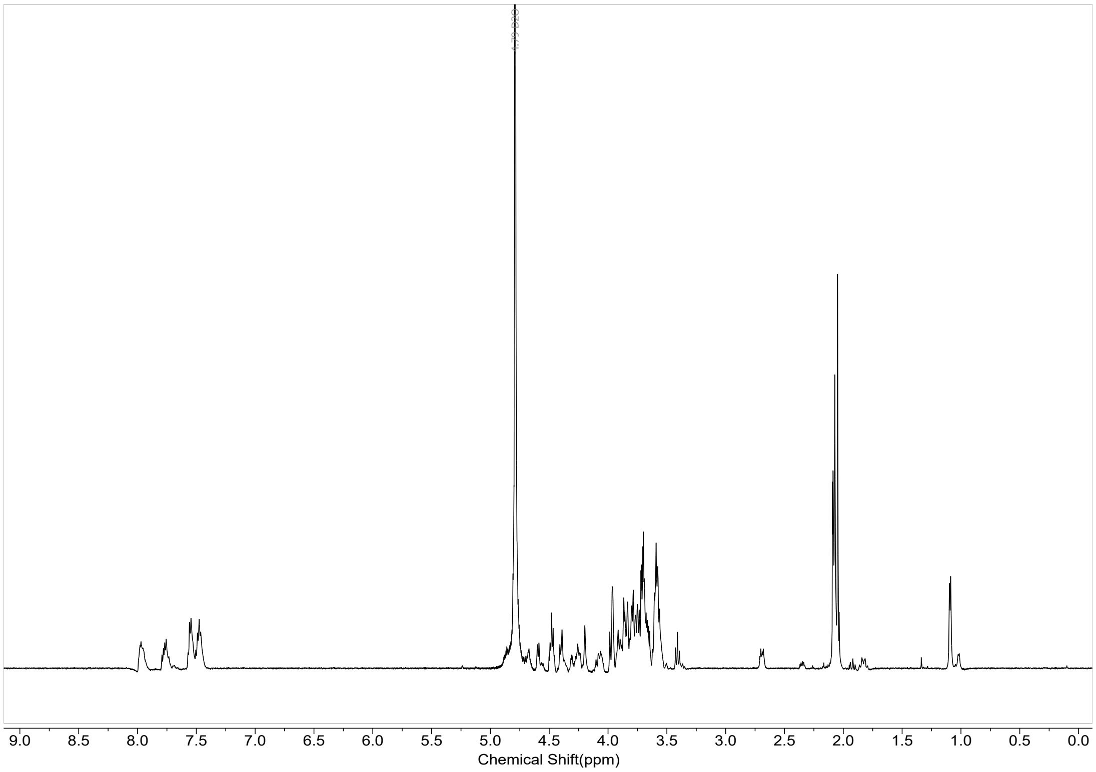

^{1}H NMR of Compound **44**

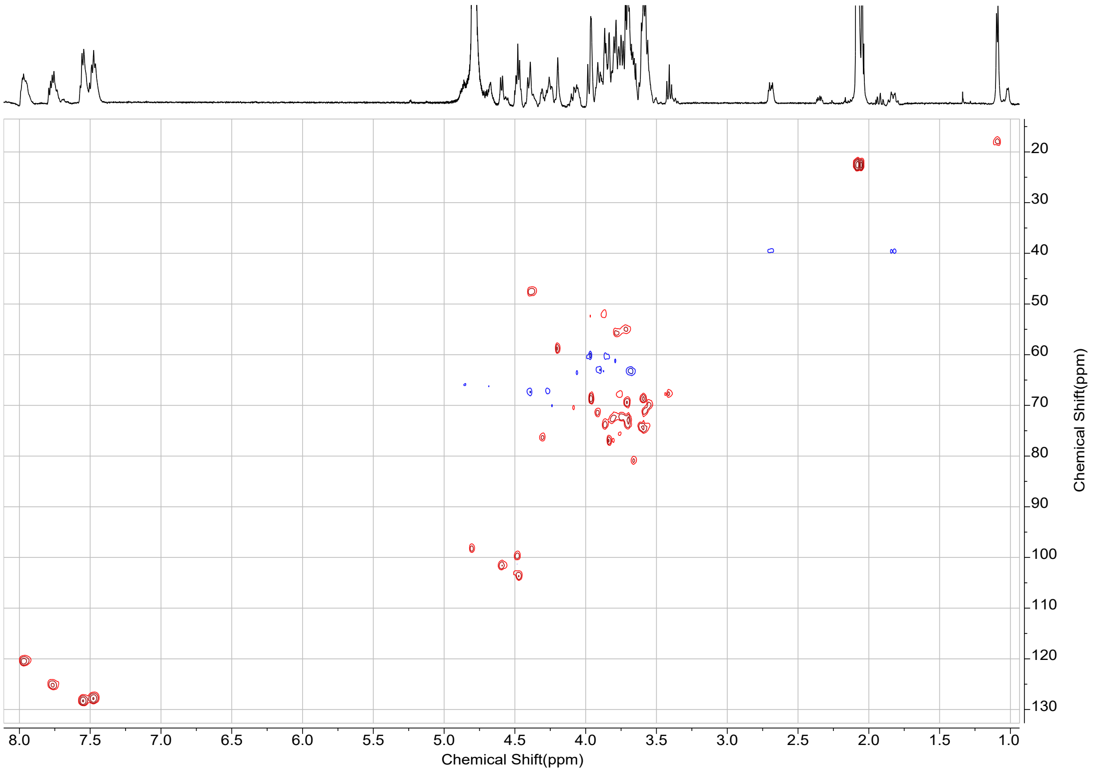

HSQC spectra of Compound **44**

Compound 45

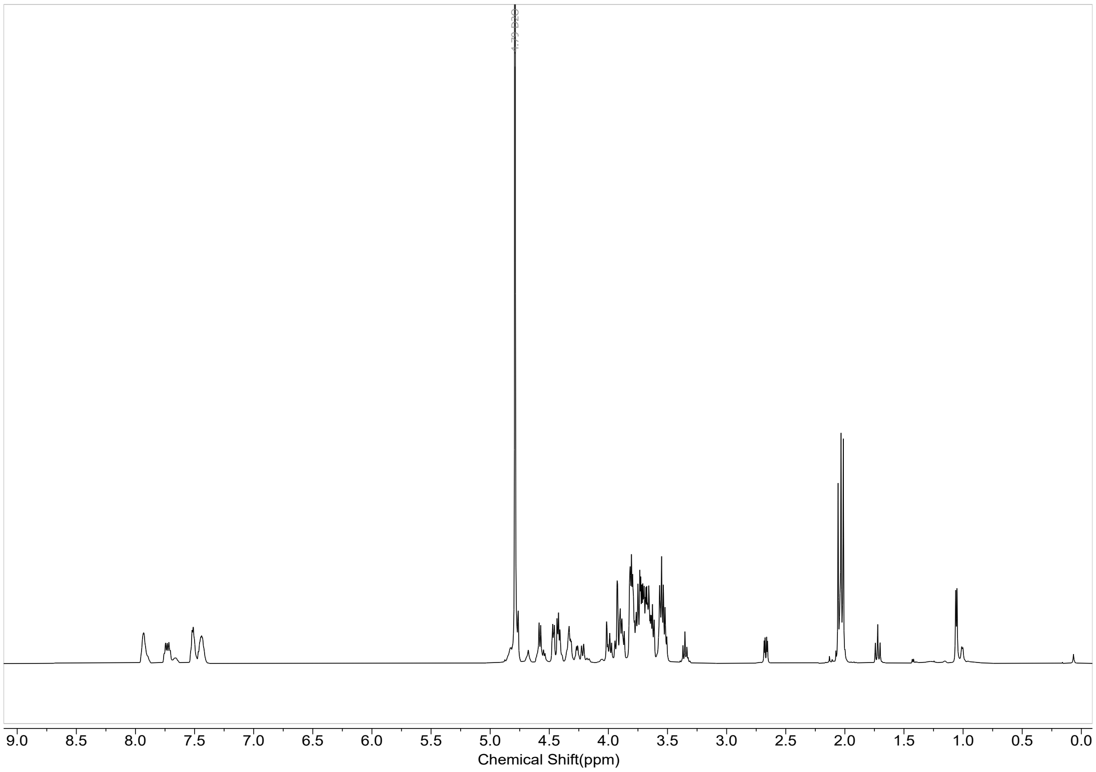

^{1}H NMR of Compound **45**

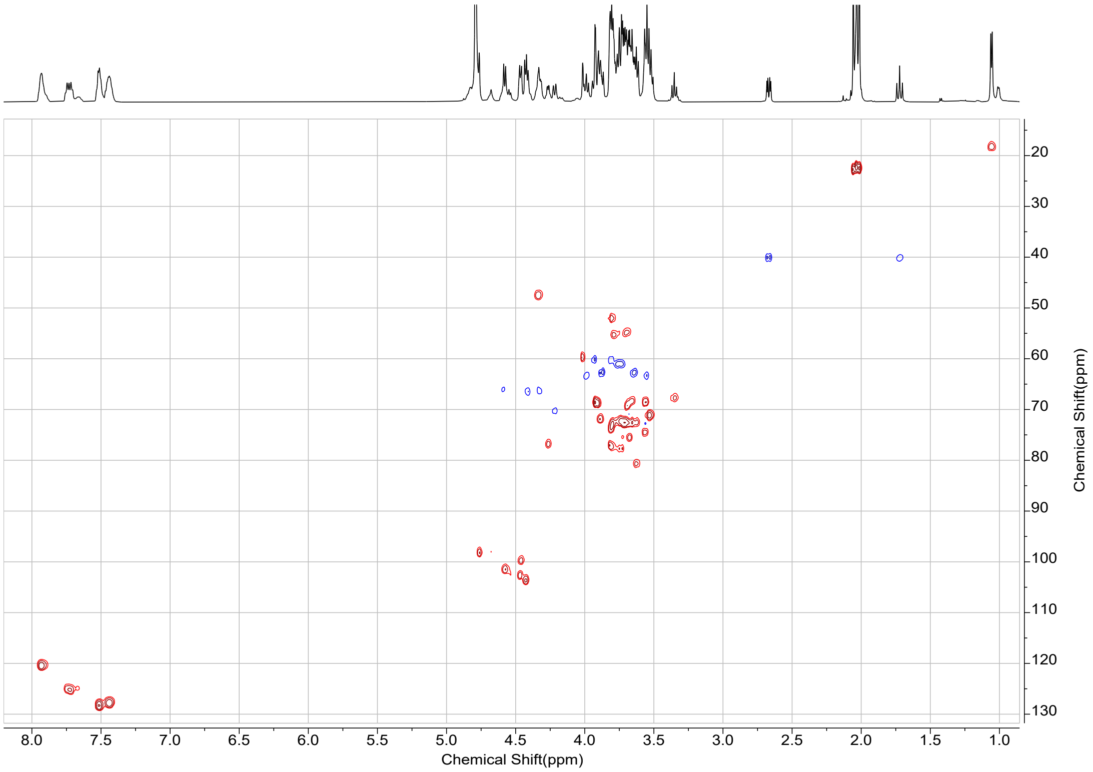

HSQC spectra of Compound **45**

Compound 46

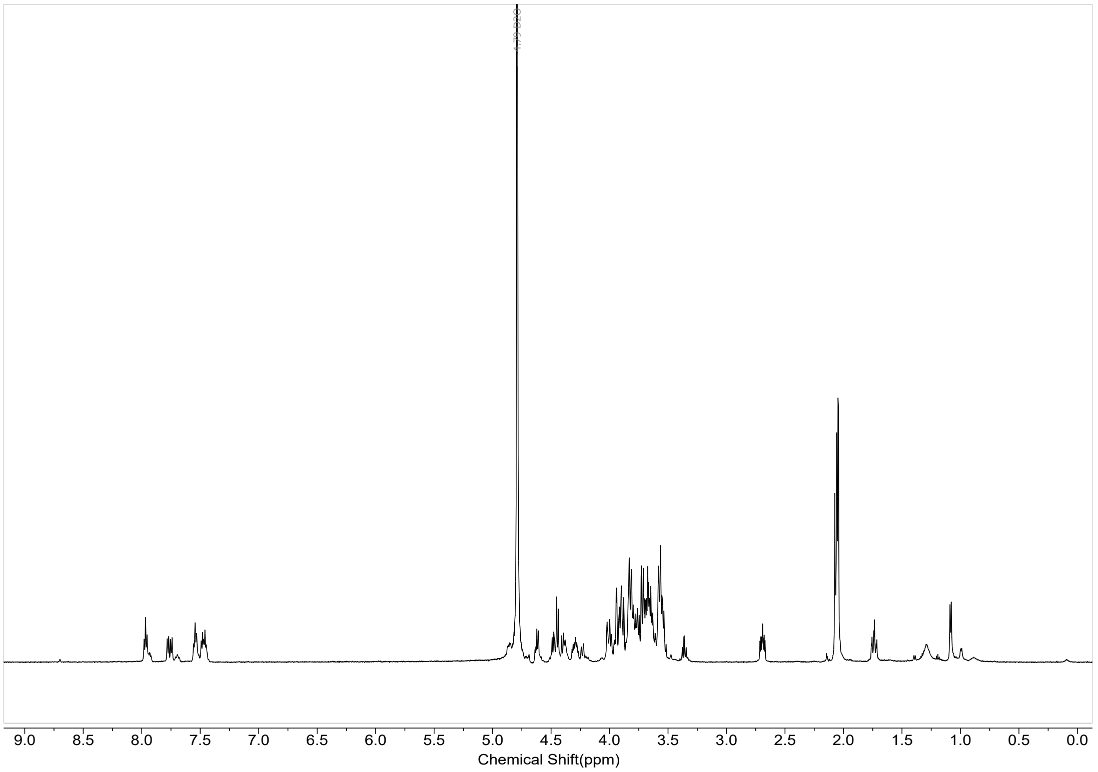

^{1}H NMR of Compound **46**

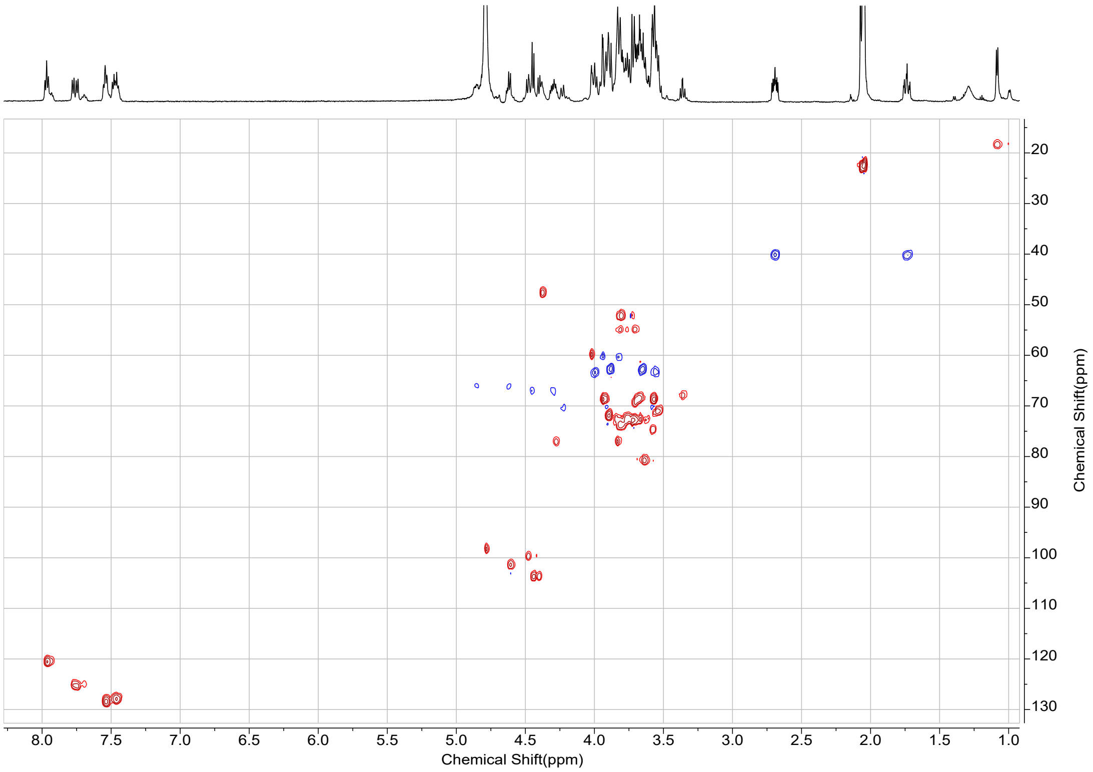

HSQC spectra of Compound **46**

Compound 47

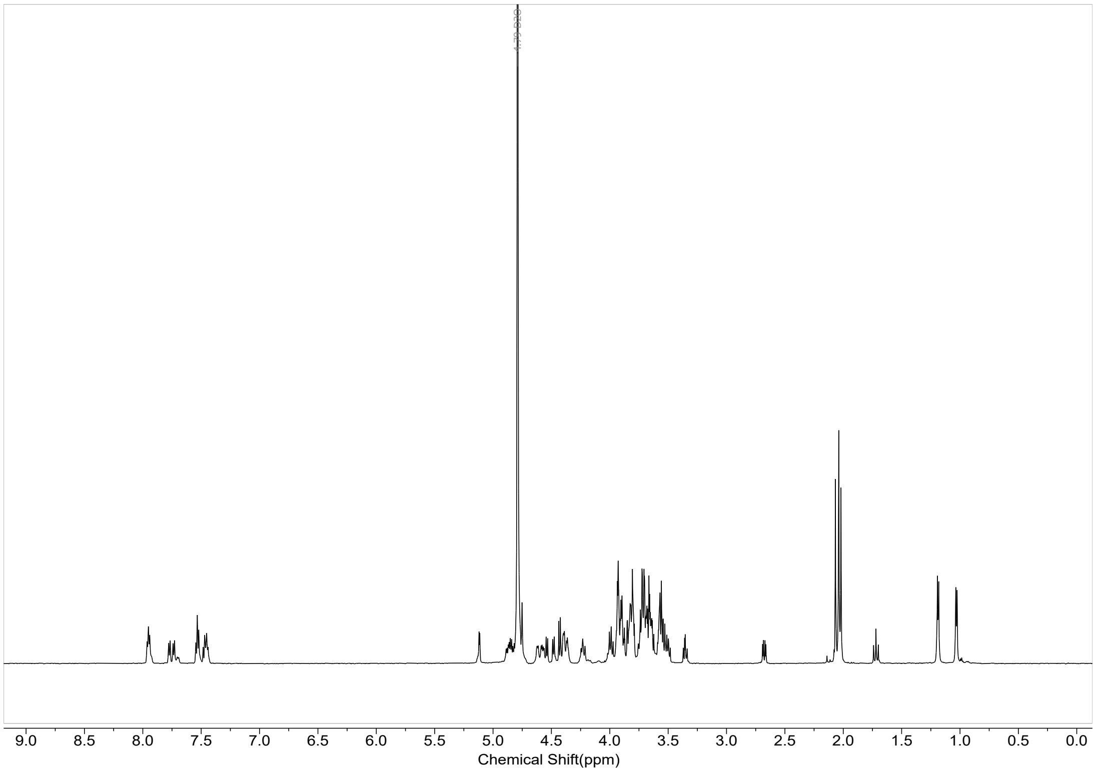

^{1}H NMR of Compound **47**

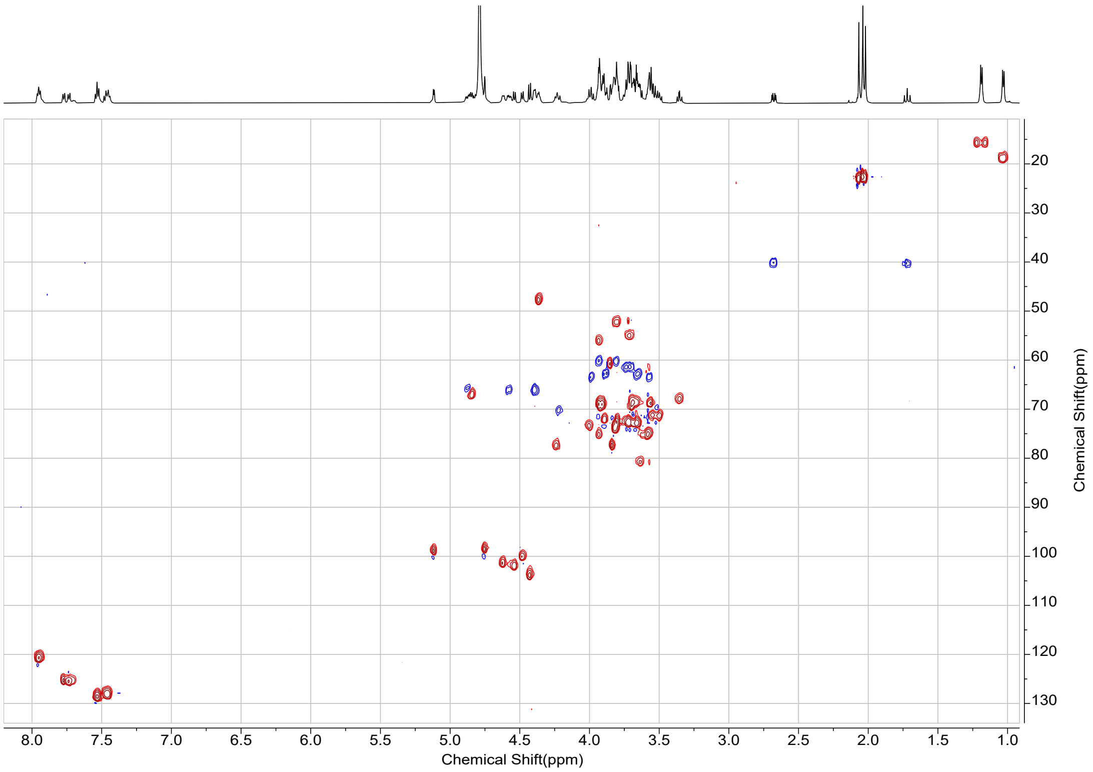

HSQC spectra of Compound **47**

Compound 48

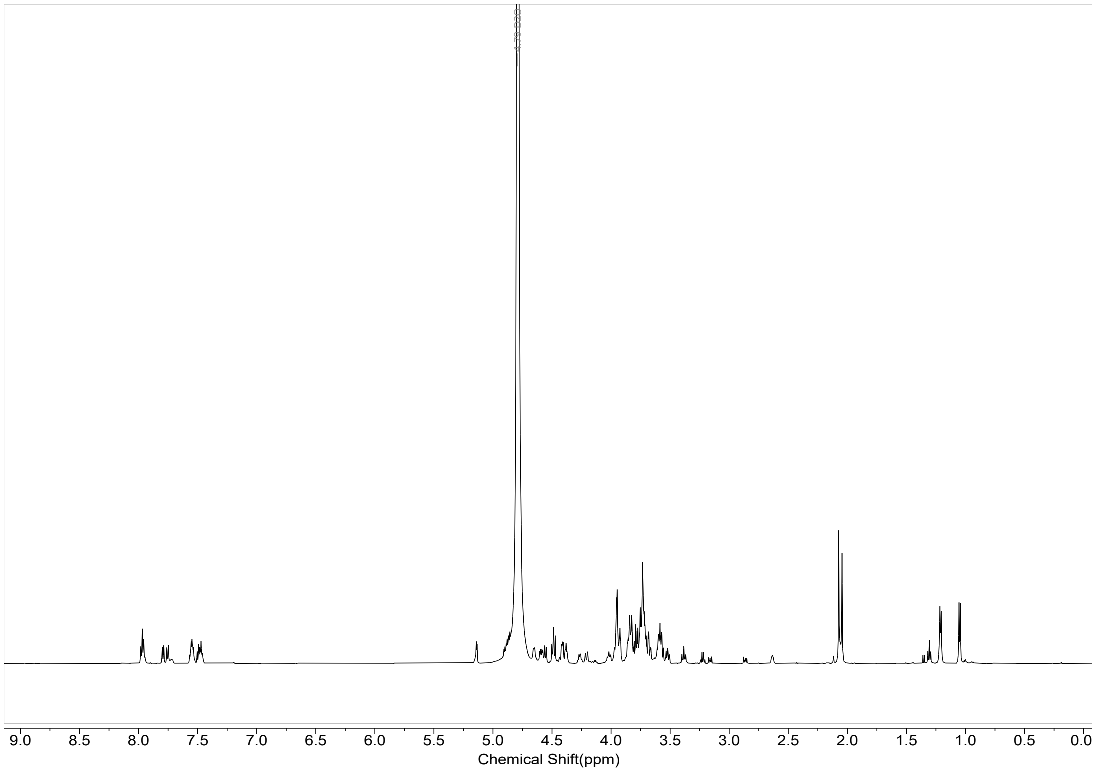

^{1}H NMR of Compound **48**

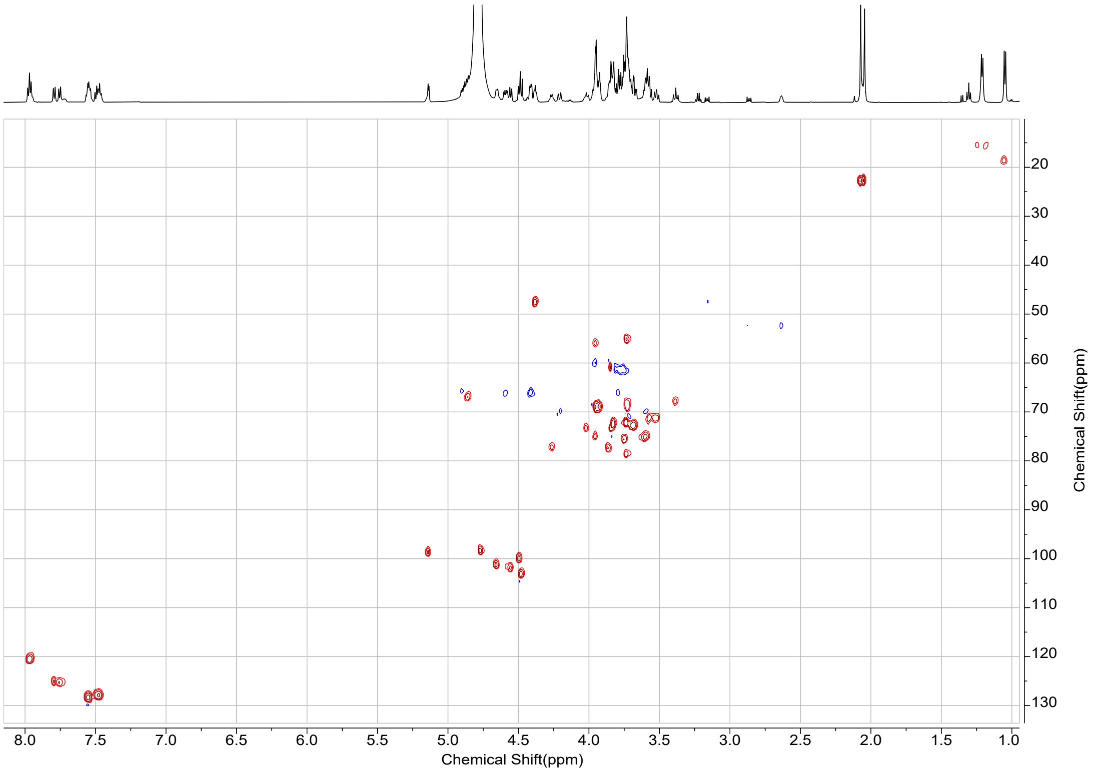

HSQC spectra of Compound **48**

VI. References

1. Peng, W.; Pranskevich, J.; Nycholat, C.; Gilbert, M.; Wakarchuk, W.; Paulson, J. C.; Razi, N., Helicobacter pylori beta1,3-N-acetylglucosaminyltransferase for versatile synthesis of type 1 and type 2 poly-LacNAcs on N-linked, O-linked and I-antigen glycans. *Glycobiology* **2012,** *22* (11), 1453-64.
2. Lau, K.; Thon, V.; Yu, H.; Ding, L.; Chen, Y.; Muthana, M. M.; Wong, D.; Huang, R.; Chen, X., Highly efficient chemoenzymatic synthesis of beta1-4-linked galactosides with promiscuous bacterial beta1-4-galactosyltransferases. *Chem Commun (Camb)* **2010,** *46* (33), 6066-8.
3. Lin, S. W.; Yuan, T. M.; Li, J. R.; Lin, C. H., Carboxyl terminus of Helicobacter pylori alpha1,3-fucosyltransferase determines the structure and stability. *Biochemistry* **2006,** *45* (26), 8108-16.
4. Liu, Y.; Wen, L.; Li, L.; Gadi, M. R.; Guan, W.; Huang, K.; Xiao, Z.; Wei, M.; Ma, C.; Zhang, Q.; Yu, H.; Chen, X.; Wang, P. G.; Fang, J., A General Chemoenzymatic Strategy for the Synthesis of Glycosphingolipids. *European J Org Chem* **2016,** *2016* (25), 4315-4320.
5. Yu, H.; Li, Y.; Wu, Z.; Li, L.; Zeng, J.; Zhao, C.; Wu, Y.; Tasnima, N.; Wang, J.; Liu, H.; Gadi, M. R.; Guan, W.; Wang, P. G.; Chen, X., H. pylori α1-3/4-fucosyltransferase (Hp3/4FT)-catalyzed one-pot multienzyme (OPME) synthesis of Lewis antigens and human milk fucosides. *Chem Commun (Camb)* **2017,** *53* (80), 11012-11015.
6. Gadi, M. R.; Chen, C.; Bao, S.; Wang, S.; Guo, Y.; Han, J.; Xiao, W.; Li, L., Convergent chemoenzymatic synthesis of O-GalNAc rare cores 5, 7, 8 and their sialylated forms. *Chem Sci* **2023,** *14* (7), 1837-1843.
7. Prudden, A. R.; Liu, L.; Capicciotti, C. J.; Wolfert, M. A.; Wang, S.; Gao, Z.; Meng, L.; Moremen, K. W.; Boons, G.-J., Synthesis of asymmetrical multiantennary human milk oligosaccharides. *Proceedings of the National Academy of Sciences* **2017,** *114* (27), 6954-6959.
8. Moremen, K. W.; Ramiah, A.; Stuart, M.; Steel, J.; Meng, L.; Forouhar, F.; Moniz, H. A.; Gahlay, G.; Gao, Z.; Chapla, D.; Wang, S.; Yang, J.-Y.; Prabhakar, P. K.; Johnson, R.; Rosa, M. d.; Geisler, C.; Nairn, A. V.; Seetharaman, J.; Wu, S.-C.; Tong, L.; Gilbert, H. J.; LaBaer, J.; Jarvis, D. L., Expression system for structural and functional studies of human glycosylation enzymes. *Nature Chemical Biology* **2018,** *14* (2), 156-162.
9. Wang, S.; Chen, C.; Gadi, M. R.; Saikam, V.; Liu, D.; Zhu, H.; Bollag, R.; Liu, K.; Chen, X.; Wang, F.; Wang, P. G.; Ling, P.; Guan, W.; Li, L., Chemoenzymatic modular assembly of O-GalNAc glycans for functional glycomics. *Nat Commun* **2021,** *12* (1), 3573.
10. Wang, S.; Zhang, Q.; Chen, C.; Guo, Y.; Gadi, M. R.; Yu, J.; Westerlind, U.; Liu, Y.; Cao, X.; Wang, P. G.; Li, L., Facile Chemoenzymatic Synthesis of O-Mannosyl Glycans. *Angew Chem Int Ed Engl* **2018,** *57* (30), 9268-9273.
11. Bao, S.; Shen, T.; Chen, C.; Han, J.; Tajadura-Ortega, V.; Shabahang, M.; Du, Z.; Feizi, T.; Chai, W.; Li, L., Orthogonal-Group-Controlled Site-Selective I-Branching of Poly-N-acetyllactosamine Chains Reveals Unique Binding Specificities of Proteins towards I-Antigens. *Angew Chem Int Ed Engl* **2025,** *64* (11), e202420676.
